# Indole-3-Acetamido-Polyamines as Antimicrobial Agents and Antibiotic Adjuvants

**DOI:** 10.3390/biom13081226

**Published:** 2023-08-07

**Authors:** Kenneth Sue, Melissa M. Cadelis, Evangelene S. Gill, Florent Rouvier, Marie-Lise Bourguet-Kondracki, Jean Michel Brunel, Brent R. Copp

**Affiliations:** 1 School of Chemical Sciences, The University of Auckland, Private Bag 92019, Auckland 1142, New Zealand; 2 School of Medical Sciences, The University of Auckland, Private Bag 92019, Auckland 1142, New Zealand; 3 Membranes et Cibles Thérapeutiques (MCT), L’Institut National de la Santé et de la Recherche Médicale (INSERM), Aix-Marseille Universite, 27 bd Jean Moulin, 13385 Marseille, France; 4 Laboratoire Molécules de Communication et Adaptation des Micro-Organismes, UMR 7245 CNRS, Muséum National d’Histoire Naturelle, 57 Rue Cuvier (C.P. 54), 75005 Paris, France

**Keywords:** indole-3-acetamide, potentiator, antimicrobial, polyamine, antibiotics, antifungal agents, structure-activity relationships

## Abstract

The widespread incidence of antimicrobial resistance necessitates the discovery of new classes of antimicrobials as well as adjuvant molecules that can restore the action of ineffective antibiotics. Herein, we report the synthesis of a new class of indole-3-acetamido-polyamine conjugates that were evaluated for antimicrobial activities against a panel of bacteria and two fungi, and for the ability to enhance the action of doxycycline against *Pseudomonas aeruginosa* and erythromycin against *Escherichia coli*. Compounds **14b**, **15b**, **17c**, **18a**, **18b**, **18d**, **19b**, **19e**, **20c** and **20d** exhibited strong growth inhibition of methicillin-resistant *Staphylococcus aureus* (MRSA) and *Cryptococcus neoformans*, with minimum inhibitory concentrations (MIC) typically less than 0.2 µM. Four analogues, including a 5-bromo **15c** and three 5-methoxyls **16d**–**f,** also exhibited intrinsic activity towards *E. coli*. Antibiotic kill curve analysis of **15c** identified it to be a bactericide. While only one derivative was found to (weakly) enhance the action of erythromycin against *E. coli*, three examples, including **15c**, were found to be strong enhancers of the antibiotic action of doxycycline against *P. aeruginosa*. Collectively, these results highlight the promising potential of α,ω-disubstituted indole-3-acetamido polyamine conjugates as antimicrobials and antibiotic adjuvants.

## 1. Introduction

**Figure 1:**
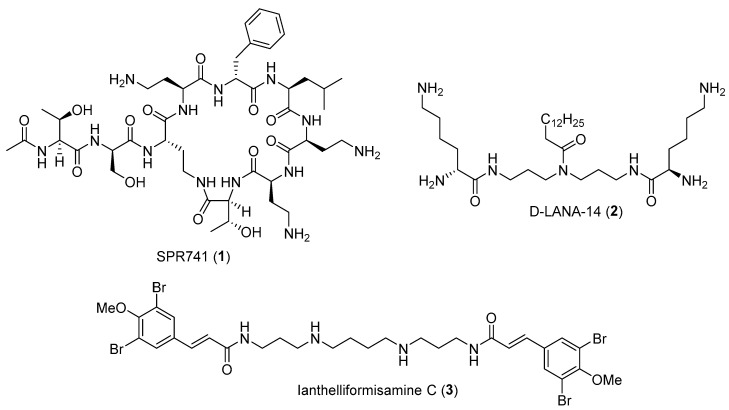
The structures of antibiotic adjuvants SPR741 (**1**), D-LANA-14 (**2**) and ianthelliformisamine C (**3**).

Antimicrobial drug resistance is a growing global health threat that requires urgent attention [1]. One approach to overcoming such a threat is to improve the effectiveness of existing antibiotics by the use of antibiotic adjuvants [2,3,4,5,6]. The only clinically approved examples of a small-molecule antibiotic adjuvant are the β-lactamase inhibitors, which inhibit a dominant mechanism of resistance towards β-lactam antibiotics [7]. As reviewed recently, the search for new chemical classes that can act as antibiotic adjuvants has revealed a number of different scaffolds [5,6]. Of interest has been the identification of molecules that perturb bacterial membranes, facilitating antibiotic entry into bacteria. Examples of such molecules include SPR741 (**1**) [8], D-LANA-14 (**2**) [9] and ianthelliformisamine C (**3**) [10] (Figure 1), all of which have been reported to enhance the action of legacy antibiotics towards resistant Gram-negative bacteria.

**Figure 2:**
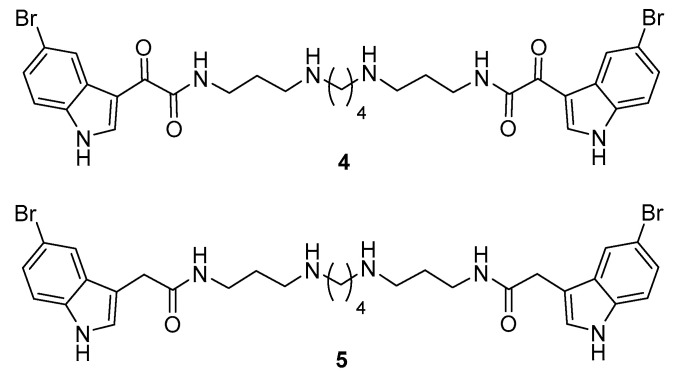
The structures of indolglyoxyl potentiator **4** and indole-3-acetamide analogue **5**.

Our search for new examples of antibiotic enhancers led to the identification of indole-3-glyoxyl-spermine **4** (Figure 2) as being able to enhance the action of doxycycline towards the Gram-negative bacteria *Pseudomonas aeruginosa*, *Escherichia coli* and *Klebsiella pneumoniae* [11]. Synthesis and biological evaluation of a larger library of indole-3-glyoxylamido-polyamines gave mixed results, identifying several analogues with increased intrinsic antimicrobial activities, but none with improved antibiotic enhancement properties [12].

The majority of analogues prepared in the latter study also exhibited cytotoxicity towards human embryonic kidney cells (HEK293)and/or hemolytic activity towards human red blood cells, limiting any potential utility. As the indole-3-glyoxylamide moiety is present in a diverse array of cytotoxic compounds, including tubulin-targeting agents [13,14], we sought another indole-based capping group that could replace it. Cadelis et al. showed that a 5-bromoindole-3-acetamide derivative of spermine, **5** (Figure 2) exhibited strong enhancement of the antibiotic action of doxycycline towards *P. aeruginosa* (minimum inhibitory concentration (MIC) 6.25 µM), *E. coli* (MIC 3.125 µM) and *K. pneumoniae* (MIC 6.25 µM), with negligible cytotoxicity or hemolytic properties (Table 1) [15]. In another study, Cadelis et al. reported that 5- or 7-substituted indole capping acid-polyamine conjugates showed improved antibiotic enhancement properties with reduced cytotoxicity/hemolytic activities [16]. Taking these data together, a new series reported herein incorporated 5- and 7-substituted indole-3-acetic acid capping groups attached to a range of different length polyamines (PA). All compounds prepared were evaluated for intrinsic antimicrobial activity, using a panel of Gram-positive and Gram-negative bacteria and two fungal species, the ability to enhance the antibiotic action of doxycycline towards *P. aeruginosa* and erythromycin against *E. coli*, and for cytotoxicity and red blood cell hemolytic properties.

## 2. Materials and Methods

### 2.1. Chemistry: General remarks

Infrared spectra were recorded on a Perkin-Elmer spectrometer 100 Fourier Transform infrared spectrometer equipped with a universal ATR accessory. Mass spectra were acquired on a Bruker micrOTOF Q II spectrometer. ^1^H and ^13^C NMR spectra were recorded at 298 K on a Bruker AVANCE 400 spectrometer using standard pulse sequences. Proto-deutero solvent signals were used as internal references (CD_3_OD: δ_H_ 3.31, δ_C_ 49.00). For ^1^H NMR, the data are quoted as position (δ), relative integral, multiplicity (s = singlet, d = doublet, t = triplet, q = quartet, dt = doublet of triplets, td = triplet of doublets, tt = triplet of triplets, ddd = doublet of doublet of doublets, m = multiplet, br = broad), coupling constant (*J*, Hz) and assignment to the atom. The ^13^C NMR data are quoted as position (δ), coupling constant (*J*_CF_, Hz) and assignment to the atom. Flash column chromatography was carried out using Davisil silica gel (40–60 μm) or Merck LiChroprep RP-8 (40–63 µm). Thin layer chromatography was conducted on Merck DC Kieselgel 60 RP-18 F254S plates. All solvents used were of analytical grade or better and/or purified according to standard procedures. Chemical reagents used were purchased from standard chemical suppliers and used as purchased. The indole-3-acetic acids utilized in this study (**6**–**12**) were all commercially available, while protected polyamines di-*tert*-butyl butane-1,4-diylbis((3-aminopropyl)carbamate) (**13a**), di-*tert*-butyl hexane-1,6-diylbis((3-aminopropyl)carbamate) (**13b**), di-*tert*-butyl heptane-1,7-diylbis((3-aminopropyl)carbamate) (**13c**), di-*tert*-butyl octane-1,8-diylbis((3-aminopropyl)carbamate) (**13d**), di-*tert*-butyl decane-1,10-diylbis((3-aminopropyl)carbamate) (**13e**) and di-*tert*-butyl dodecane-1,12-diylbis((3-aminopropyl)carbamate) (**13f**) [17,18,19,20] and target conjugates **14a**, **15a** and **16a** [15] were synthesized using previously reported protocols.

#### 2.1.1. General Procedure A—Coupling of Indole-3-Acetic Acids with Boc-Protected Polyamine

To a solution of indole-3-acetic acid (2.2 equiv.) in either CH_2_Cl_2_ (2 mL) or DMF (1 mL) was added EDC**·**HCl (2.6 equiv.), HOBt (2.6 equiv.) and DIPEA (6 equiv.) at 0 °C, and the mixture was stirred for 30 min. Boc-protected polyamine (1.0 equiv.) was added and the mixture allowed to come to room temperature and stirred for a further 24 h under N_2_. The reaction mixture was poured into CH_2_Cl_2_ (20 mL) and washed with sat. aq. NaHCO_3_ (2 × 30 mL) followed by H_2_O (2 × 30 mL), then dried under reduced pressure and purified with silica gel flash column chromatography (0–3% MeOH/CH_2_Cl_2_) to afford the desired product.

#### 2.1.2. General Procedure B—Boc Deprotection

A solution of *tert*-butyl-carbamate derivative in CH_2_Cl_2_ (2 mL) and TFA (0.2 mL) was stirred at room temperature under N_2_ for 2 h followed by solvent removal under reduced pressure. The crude product was purified using C_8_ reversed-phase flash column chromatography eluting with 0–50% MeOH/H_2_O (0.05% TFA) to afford the desired polyamine as a di-TFA salt.

### 2.2. Synthesis of Compounds

#### 2.2.1. *N*^1^,*N*^6^-Bis(3-(2-(1H-indol-3-yl)acetamido)propyl)hexane-1,6-diaminium 2,2,2-trifluoroacetate (**14b**)

Following general procedure A, indole-3-acetic acid (**6**) (0.050 g, 0.285 mmol) was reacted with EDC**·**HCl (0.065 g, 0.337 mmol), HOBt (0.046 g, 0.337 mmol), DIPEA (0.14 mL, 0.778 mmol) and di-*tert*-butyl hexane-1,6-diylbis((3-aminopropyl)carbamate) (**13b**) (0.056 g, 0.130 mmol) to afford di-*tert*-butyl hexane-1,6-diylbis((3-(2-(1*H*-indol-3-yl)acetamido)propyl)carbamate) (0.022 g, 23%) as a clear colorless oil. Following general procedure B, a sub-sample of this product (0.011 g, 0.015 mmol) was reacted with TFA in CH_2_Cl_2_ to afford, after chromatography, the di-TFA salt **14b** (0.011 g, 96%) as a white gum. R*_f_* (RP-18, 10% *aq* HCl:MeOH 1:3) 0.63; IR (ATR) ν_max_ 3262, 3059, 2932, 2857, 1668, 1619, 1541, 1471, 1435, 1330, 1198, 1178, 1129, 834, 799, 747, 720 cm^−1^; ^1^H NMR (CD_3_OD, 400 MHz) δ 7.57 (2H, d, *J* = 7.8 Hz, H-4), 7.37 (2H, dt, *J* = 8.0, 1.0 Hz, H-7), 7.21 (2H, s, H-2), 7.11 (2H, td, *J* = 11.0, 0.9 Hz, H-6), 7.05 (2H, ddd, *J* = 15.3, 7.0, 0.9 Hz, H-5), 3.69 (4H, s, H_2_-8), 3.30 (4H, obscured by solvent, H_2_-11), 2.77 (4H, t, *J* = 7.0 Hz, H_2_-13), 2.70 (4H, t, *J* = 6.3 Hz, H_2_-15), 1.79 (4H, tt, *J* = 6.6, 6.6 Hz, H_2_-12), 1.53 (4H, tt, *J* = 3.6, 3.6 Hz, H_2_-16), 1.30–1.27 (4H, m, H_2_-17); ^13^C NMR (CD_3_OD, 100 MHz) δ 176.6 (C-9), 138.3 (C-7a), 128.4 (C-3a), 125.2 (C-2), 122.7 (C-6), 120.1 (C-5), 119.3 (C-4), 112.6 (C-7), 109.4 (C-3), 48.3 (C-15, obscured by solvent), 45.9 (C-13), 36.6 (C-11), 34.0 (C-8), 27.7 (C-12), 26.9 (C-17), 26.8 (C-16); (+)-HRESIMS [M + H]^+^ *m/z* 545.3596 (calcd for C_32_H_45_N_6_O_2_, 545.3599).

#### 2.2.2. *N*^1^,*N*^7^-Bis(3-(2-(1*H*-indol-3-yl)acetamido)propyl)heptane-1,7-diaminium 2,2,2-trifluoroacetate (**14c**)

Following general procedure A, indole-3-acetic acid (**6**) (0.050 g, 0.285 mmol) was reacted with EDC**·**HCl (0.065 g, 0.337 mmol), HOBt (0.046 g, 0.337 mmol), DIPEA (0.14 mL, 0.778 mmol) and di-*tert*-butyl heptane-1,7-diylbis((3-aminopropyl)carbamate) (**13c**) (0.058 g, 0.130 mmol) to afford di-*tert*-butyl octane-1,8-diylbis((3-(2-(1*H*-indol-3-yl)acetamido)propyl)carbamate) (0.044 g, 45%) as a clear colorless oil. Following general procedure B, a sub-sample of this product (0.022 g, 0.029 mmol) was reacted with TFA in CH_2_Cl_2_ to afford, after chromatography, the di-TFA salt **14c** (0.018 g, 79%) as a brown oil. R*_f_* (RP-18, 10% *aq* HCl:MeOH 1:3) 0.60; IR (ATR) ν_max_ 3262, 3059, 2932, 2857, 1668, 1619, 1541, 1471, 1435, 1330, 1199, 1178, 1128, 835, 799, 747, 720 cm^−1^; ^1^H NMR (CD_3_OD, 400 MHz) δ 7.57 (2H, d, *J* = 7.9 Hz, H-4), 7.37 (2H, dt, *J* = 8.3, 1.0 Hz, H-7), 7.21 (2H, s, H-2), 7.12 (2H, ddd, *J* = 15.3, 6.8, 0.9 Hz, H-6), 7.03 (2H, ddd, *J* = 15.0, 7.0, 0.9 Hz, H-5), 3.69 (4H, s, H_2_-8), 3.30 (4H, obscured by solvent, H_2_-11), 2.77 (4H, t, *J* = 7.1 Hz, H_2_-13), 2.73 (4H, t, *J* = 7.8 Hz, H_2_-15), 1.79 (4H, tt, *J* = 6.8, 6.5 Hz, H_2_-12), 1.58–1.52 (4H, m, H_2_-16), 1.34–1.29 (6H, m, H_2_-17 and H_2_-18); ^13^C NMR (CD_3_OD, 100 MHz) δ 176.5 (C-9), 138.2 (C-7a), 128.4 (C-3a), 125.2 (C-2), 122.7 (C-6), 120.1 (C-5), 119.3 (C-4), 112.6 (C-7), 109.4 (C-3), 48.7 (C-15, obscured by solvent), 45.9 (C-13), 36.7 (C-11), 34.0 (C-8), 29.5 (C-18), 27.6 (C-12), 27.2 (C-17), 27.0 (C-16); (+)-HRESIMS [M + H]^+^ *m/z* 559.3755 (calcd for C_33_H_47_N_6_O_2_, 559.3755).

#### 2.2.3. *N*^1^,*N*^8^-Bis(3-(2-(1*H*-indol-3-yl)acetamido)propyl)octane-1,8-diaminium 2,2,2-trifluoroacetate (**14d**)

Following general procedure A, indole-3-acetic acid (**6**) (0.050 g, 0.285 mmol) was reacted with EDC**·**HCl (0.065 g, 0.337 mmol), HOBt (0.046 g, 0.337 mmol), DIPEA (0.14 mL, 0.778 mmol) and di-*tert*-butyl octane-1,8-diylbis((3-aminopropyl)carbamate) (**13d**) (0.060 g, 0.130 mmol) to afford di-*tert*-butyl octane-1,8-diylbis((3-(2-(1*H*-indol-3-yl)acetamido)propyl)carbamate) (0.022 g, 22%) as a clear colorless oil. Following general procedure B, a sub-sample of this product (0.011 g, 0.014 mmol) was reacted with TFA in CH_2_Cl_2_ to afford, after chromatography, the di-TFA salt **14d** (0.10 g, 88%) as a brown oil. R*_f_* (RP-18, 10% *aq* HCl:MeOH 1:3) 0.57; IR (ATR) ν_max_ 3266, 3063, 2933, 2857, 1668, 1620, 1470, 1435, 1330, 1199, 1178, 1128, 835, 799, 747, 720 cm^−1^; ^1^H NMR (CD_3_OD, 400 MHz) δ 7.57 (2H, d, *J* = 7.9 Hz, H-4), 7.37 (2H, dt, *J* = 7.8, 1.0 Hz, H-7), 7.21 (2H, s, H-2), 7.12 (2H, ddd, *J* = 8.2, 7.0, 1.1 Hz, H-6), 7.03 (2H, ddd, *J* = 7.8, 6.9, 0.8 Hz, H-5), 3.69 (4H, s, H_2_-8), 3.30 (4H, obscured by solvent, H_2_-11), 2.78 (4H, t, *J* = 7.1 Hz, H_2_-13), 2.73 (4H, t, *J* = 7.8 Hz, H_2_-15), 1.79 (4H, tt, *J* = 6.8, 6.7 Hz, H_2_-12), 1.59–1.52 (4H, m, H_2_-16), 1.34–1.31 (8H, m, H_2_-17 and H_2_-18); ^13^C NMR (CD_3_OD, 100 MHz) δ 176.4 (C-9), 138.2 (C-7a), 128.4 (C-3a), 125.2 (C-2), 122.7 (C-6), 120.1 (C-5), 119.3 (C-4), 112.6 (C-7), 109.4 (C-3), 48.5 (C-15, obscured by solvent), 46.0 (C-13), 36.7 (C-11), 34.0 (C-8), 29.8 (C-18), 27.6 (C-12), 27.3 (C-17), 27.2 (C-16); (+)-HRESIMS [M + H]^+^ *m/z* 573.3911 (calcd for C_34_H_49_N_6_O_2_, 573.3912).

#### 2.2.4. *N*^1^,*N*^10^-Bis(3-(2-(1*H*-indol-3-yl)acetamido)propyl)decane-1,10-diaminium 2,2,2-trifluoroacetate (**14e**)

Following general procedure A, indole-3-acetic acid (**6**) (0.050 g, 0.285 mmol) was reacted with EDC**·**HCl (0.065 g, 0.337 mmol), HOBt (0.046 g, 0.337 mmol), DIPEA (0.14 mL, 0.778 mmol) and di-*tert*-butyl decane-1,10-diylbis((3-aminopropyl)carbamate) (**13e**) (0.063 g, 0.130 mmol) to yield di-*tert*-butyl decane-1,10-diylbis((3-(2-(1*H*-indol-3-yl)acetamido)propyl)carbamate) (0.082 g, 79%) as a clear colorless oil. Following general procedure B, a sub-sample of this product (0.048 g, 0.060 mmol) was reacted with TFA in CH_2_Cl_2_ to afford, after chromatography, the di-TFA salt **14e** (0.043 g, 86%) as a pink-brown oil. R*_f_* (RP-18, 10% *aq* HCl:MeOH 1:3) 0.35; IR (ATR) ν_max_ 3267, 3062, 2933, 2857, 1668, 1621, 1471, 1434, 1330, 1199, 1177, 1128, 834, 799, 748, 720 cm^−1^; ^1^H NMR (CD_3_OD, 400 MHz) δ 7.57 (2H, d, *J* = 7.9 Hz, H-4), 7.37 (2H, d, *J* = 8.0 Hz, H-7), 7.21 (2H, s, H-2), 7.12 (2H, td, *J* = 7.5, 1.0 Hz, H-6), 7.03 (2H, ddd, *J* = 7.9, 7.1, 0.9 Hz, H-5), 3.68 (4H, s, H_2_-8), 3.28 (4H, t, *J* = 6.7 Hz, H_2_-11), 2.77 (4H, t, *J* = 7.5 Hz, H_2_-13), 2.71 (4H, t, *J* = 7.8 Hz, H_2_-15), 1.79 (4H, tt, *J* = 6.9, 6.9 Hz, H_2_-12), 1.54 (4H, tt, *J* = 7.5, 7.5 Hz, H_2_-16), 1.38–1.29 (12H, m, H_2_-17, H_2_-18 and H_2_-19); ^13^C NMR (CD_3_OD, 100 MHz) δ 176.4 (C-9), 138.2 (C-7a), 128.4 (C-3a), 125.2 (C-2), 122.7 (C-6), 120.0 (C-5), 119.3 (C-4), 112.6 (C-7), 109.4 (C-3), 48.9 (C-15), 46.0 (C-13), 36.7 (C-11), 34.0 (C-8), 30.3 (C-19), 30.1 (C-18), 27.6 (C-12), 27.4 (C-17), 27.1 (C-16); (+)-HRESIMS [M + H]^+^ *m/z* 601.4224 (calcd for C_36_H_53_N_6_O_2_, 601.4225).

#### 2.2.5. *N*^1^,*N*^12^-Bis(3-(2-(1*H*-indol-3-yl)acetamido)propyl)dodecane-1,12-diaminium 2,2,2-trifluoroacetate (**14f**)

Following general procedure A, indole-3-acetic acid (**6**) (0.050 g, 0.285 mmol) was reacted with EDC**·**HCl (0.065 g, 0.337 mmol), HOBt (0.046 g, 0.337 mmol), DIPEA (0.14 mL, 0.778 mmol) and di-*tert*-butyl dodecane-1,12-diylbis((3-aminopropyl)carbamate) (**13f**) (0.067 g, 0.130 mmol) to afford di-*tert*-butyl dodecane-1,12-diylbis((3-(2-(1*H*-indol-3-yl)acetamido)propyl)carbamate) (0.050 g, 46%) as a pale yellow oil. Following general procedure B, a sub-sample of this product (0.030 g, 0.036 mmol) was reacted with TFA in CH_2_Cl_2_ to afford, after chromatography, the di-TFA salt **14f** (0.023 g, 74%) as a brown oil. R*_f_* (RP-18, 10% *aq* HCl:MeOH 1:3) 0.30; IR (ATR) ν_max_ 3267, 3064, 2927, 2854, 1668, 1621, 1538, 1471, 1435, 1333, 1199, 1178, 1128, 835, 799, 750, 720 cm^−1^; ^1^H NMR (CD_3_OD, 400 MHz) δ 7.57 (2H, d, *J* = 7.8 Hz, H-4), 7.37 (2H, d, *J* = 8.3 Hz, H-7), 7.21 (2H, s, H-2), 7.12 (2H, td, *J* = 7.5, 1.0 Hz, H-6), 7.03 (2H, td, *J* = 7.5, 1.2 Hz, H-5), 3.68 (4H, s, H_2_-8), 3.28 (4H, t, *J* = 6.4 Hz, H_2_-11), 2.75 (4H, t, *J* = 7.2 Hz, H_2_-13), 2.69 (4H, t, *J* = 7.9 Hz, H_2_-15), 1.78 (4H, tt, *J* = 6.8, 6.8 Hz, H_2_-12), 1.53 (4H, tt, *J* = 7.4, 7.4 Hz, H_2_-16), 1.34–1.28 (16H, m, H_2_-17, H_2_-18, H_2_-19 and H_2_-20); ^13^C NMR (CD_3_OD, 100 MHz) δ 176.3 (C-9), 138.2 (C-7a), 128.4 (C-3a), 125.2 (C-2), 122.7 (C-6), 120.1 (C-5), 119.3 (C-4), 112.6 (C-7), 109.4 (C-3), 48.9 (C-15), 46.0 (C-13), 36.7 (C-11), 34.0 (C-8), 30.6 (C-20), 30.5 (C-19), 30.1 (C-18), 27.6 (C-12), 27.4 (C-17), 27.1 (C-16); (+)-HRESIMS [M + H]^+^ *m/z* 629.4537 (calcd for C_38_H_57_N_6_O_2_, 629.4538).

#### 2.2.6. *N*^1^,*N*^6^-Bis(3-(2-(5-bromo-1*H*-indol-3-yl)acetamido)propyl)hexane-1,6-diaminium 2,2,2-trifluoroacetate (**15b**)

Following general procedure A, 5-bromoindole-3-acetic acid (**7**) (0.050 g, 0.197 mmol) was reacted with EDC**·**HCl (0.045 g, 0.233 mmol), HOBt (0.031 g, 0.233 mmol), DIPEA (0.09 mL, 0.537 mmol) and di-*tert*-butyl hexane-1,6-diylbis((3-aminopropyl)carbamate) (**13b**) (0.039 g, 0.0894 mmol) to afford di-*tert*-butyl hexane-1,6-diylbis((3-(2-(5-bromo-1*H*-indol-3-yl)acetamido)propyl)carbamate) (0.064 g, 79%) as a clear colorless oil. Following general procedure B, a sub-sample of this product (0.028 g, 0.031 mmol) was reacted with TFA in CH_2_Cl_2_ to afford, after chromatography, the di-TFA salt **15b** (0.027 g, 94%) as an orange oil. R*_f_* (RP-18, 10% *aq* HCl:MeOH 1:3) 0.35; IR (ATR) ν_max_ 3264, 2941, 2852, 1668, 1554, 1471, 1199, 1178, 1128, 884, 834, 798, 720 cm^−1^; ^1^H NMR (CD_3_OD, 400 MHz) δ 7.75 (2H, d, *J* = 1.8 Hz, H-4), 7.30 (2H, d, *J* = 8.2 Hz, H-7), 7.24 (2H, s, H-2), 7.21 (2H, dd, *J* = 8.6, 1.9 Hz, H-6), 3.66 (4H, s, H_2_-8), 3.31 (4H, obscured by solvent, H_2_-11), 2.82 (4H, t, *J* = 6.9 Hz, H_2_-13), 2.79 (4H, t, *J* = 7.6 Hz. H_2_-15), 1.82 (4H, tt, *J* = 6.7, 6.6 Hz, H_2_-12), 1.62–1.55 (4H, m, H_2_-16), 1.33 (4H, tt, *J* = 3.7, 3.7 Hz, H_2_-17); ^13^C NMR (CD_3_OD, 100 MHz) δ 176.0 (C-9), 136.8 (C-7a), 130.3 (C-3a), 126.7 (C-2), 125.4 (C-6), 122.1 (C-4), 114.3 (C-7), 113.2 (C-5), 109.4 (C-3), 49.1 (C-15), 46.0 (C-13), 36.7 (C-11), 33.8 (C-8), 29.6 (C-18), 27.7 (C-12), 26.94 (C-17), 26.88 (C-16); (+)-HRESIMS [M + H]^+^ *m/z* 701.1800 (calcd for C_32_H_43_^79^Br_2_N_6_O_2_, 701.1809), 703.1784 (calcd for C_32_H_43_^79^Br^81^BrN_6_O_2_, 703.1791), 705.1772 (calcd for C_32_H_43_^81^Br_2_N_6_O_2_, 705.1778).

#### 2.2.7. *N*^1^,*N*^7^-Bis(3-(2-(5-bromo-1*H*-indol-3-yl)acetamido)propyl)heptane-1,7-diaminium 2,2,2-trifluoroacetate (**15c**)

Following general procedure A, 5-bromoindole-3-acetic acid (**7**) (0.050 g, 0.197 mmol) was reacted with EDC**·**HCl (0.045 g, 0.233 mmol), HOBt (0.031 g, 0.233 mmol), DIPEA (0.09 mL, 0.537 mmol) and di-*tert*-butyl heptane-1,7-diylbis((3-aminopropyl)carbamate) (**13c**) (0.040 g, 0.0894 mmol) to afford di-*tert*-butyl heptane-1,7-diylbis((3-(2-(1*H*-indol-3-yl)acetamido)propyl)carbamate) (0.060 g, 73%) as a clear colorless oil. Following general procedure B, a sub-sample of this product (0.031 g, 0.034 mmol) was reacted with TFA in CH_2_Cl_2_ to afford, after chromatography, the di-TFA salt **15c** (0.030 g, 94%) as a yellow oil. R*_f_* (RP-18, 10% *aq* HCl:MeOH 1:3) 0.35; IR (ATR) ν_max_ 2940, 2860, 1671, 1467, 1178, 1129, 884, 835, 798, 720 cm^−1^; ^1^H NMR (CD_3_OD, 400 MHz) δ 7.75 (2H, d, *J* = 1.9 Hz, H-4), 7.30 (2H, d, *J* = 8.8 Hz, H-7), 7.24 (2H, s, H-2), 7.21 (2H, dd, *J* = 8.6, 1.9 Hz, H-6), 3.65 (4H, s, H_2_-8), 3.31 (4H, obscured by solvent, H_2_-11), 2.82 (4H, t, *J* = 6.6 Hz, H_2_-13), 2.78 (4H, t, *J* = 7.5 Hz, H_2_-15), 1.81 (4H, tt, *J* = 6.8, 6.7 Hz, H_2_-12), 1.58 (4H, tt, *J* = 7.7, 7.4 Hz, H_2_-16), 1.38–1.30 (6H, m, H_2_-17 and H_2_-18); ^13^C NMR (CD_3_OD, 100 MHz) δ 176.0 (C-9), 136.8 (C-7a), 130.3 (C-3a), 126.7 (C-2), 125.4 (C-6), 122.1 (C-4), 114.3 (C-7), 113.2 (C-5), 109.4 (C-3), 49.1 (C-15), 46.0 (C-13), 36.7 (C-11), 33.8 (C-8), 29.6 (C-18), 27.7 (C-12), 27.2 (C-17), 27.1 (C-16); (+)-HRESIMS [M + H]^+^ *m/z* 715.1945 (calcd for C_33_H_45_^79^Br_2_N_6_O_2_, 715.1965), 717.1906 (calcd for C_33_H_45_^79^Br^81^BrN_6_O_2_, 717.1948), 719.1872 (calcd for C_33_H_45_^81^Br_2_N_6_O_2_, 719.1935).

#### 2.2.8. *N*^1^,*N*^8^-Bis(3-(2-(5-bromo-1*H*-indol-3-yl)acetamido)propyl)octane-1,8-diaminium 2,2,2-trifluoroacetate (**15d**)

Following general procedure A, 5-bromoindole-3-acetic acid (**7**) (0.050 g, 0.197 mmol) was reacted with EDC**·**HCl (0.045 g, 0.233 mmol), HOBt (0.031 g, 0.233 mmol), DIPEA (0.09 mL, 0.537 mmol) and di-*tert*-butyl octane-1,8-diylbis((3-aminopropyl)carbamate) (**13d**) (0.036 g, 0.089 mmol) to afford di-*tert*-butyl octane-1,8-diylbis((3-(2-(5-bromo-1*H*-indol-3-yl)acetamido)propyl)carbamate) (0.052 g, 62%) as a clear colorless oil. Following general procedure B, a sub-sample of this product (0.023 g, 0.025 mmol) was reacted with TFA in CH_2_Cl_2_ to afford, after chromatography, the di-TFA salt **15d** (0.015 g, 63%) as a yellow oil. R*_f_* (RP-18, 10% *aq* HCl:MeOH 1:3) 0.33; IR (ATR) ν_max_ 2939, 2858, 1671, 1467, 1199, 1178, 1128, 884, 834, 798, 721 cm^−1^; ^1^H NMR (CD_3_OD, 400 MHz) δ 7.75 (2H, d, *J* = 1.8 Hz, H-4), 7.30 (2H, d, *J* = 8.3 Hz, H-7), 7.24 (2H, s, H-2), 7.21 (2H, dd, *J* = 7.2, 2.0 Hz, H-6), 3.65 (4H, s, H_2_-8), 3.33–3.28 (4H, obscured by solvent, H_2_-11), 2.84–2.76 (8H, m, H_2_-13 and H_2_-15), 1.81 (4H, tt, *J* = 6.7, 6.5 Hz, H_2_-12), 1.58 (4H, tt, *J* = 6.8, 6.7 Hz, H_2_-16), 1.39–1.31 (8H, m, H_2_-17 and H_2_-18); ^13^C NMR (CD_3_OD, 100 MHz) δ 176.0 (C-9), 136.8 (C-7a), 130.2 (C-3a), 126.7 (C-2), 125.4 (C-6), 122.1 (C-4), 114.2 (C-7), 113.2 (C-5), 109.4 (C-3), 48.7 (C-15, obscured by solvent), 46.0 (C-13), 36.7 (C-11), 33.8 (C-8), 29.9 (C-18), 27.7 (C-12), 27.4 (C-17), 27.2 (C-16); (+)-HRESIMS [M + H]^+^ *m/z* 729.2135 (calcd for C_34_H_47_^79^Br_2_N_6_O_2_, 729.2122), 731.2149 (calcd for C_34_H_47_^79^Br^81^BrN_6_O_2_, 731.2104), 733.2109 (calcd for C_34_H_47_^81^Br_2_N_6_O_2_, 733.2092).

#### 2.2.9. *N*^1^,*N*^10^-Bis(3-(2-(5-bromo-1*H*-indol-3-yl)acetamido)propyl)decane-1,10-diaminium 2,2,2-trifluoroacetate (**15e**)

Following general procedure A, 5-bromoindole-3-acetic acid (**7**) (0.050 g, 0.197 mmol) was reacted with EDC**·**HCl (0.045 g, 0.233 mmol), HOBt (0.031 g, 0.233 mmol), DIPEA (0.09 mL, 0.537 mmol) and di-*tert*-butyl decane-1,10-diylbis((3-aminopropyl)carbamate) (**13e**) (0.044 g, 0.0894 mmol) to yield di-*tert*-butyl decane-1,10-diylbis((3-(2-(5-bromo-1*H*-indol-3-yl)acetamido)propyl)carbamate) (0.019 g, 22%) as a clear colorless oil. Following general procedure B, a sub-sample of this product (0.012 g, 0.013 mmol) was reacted with TFA in CH_2_Cl_2_ to afford, after chromatography, the di-TFA salt **15e** (0.005 g, 40%) as a yellow oil. R*_f_* (RP-18, 10% *aq* HCl:MeOH 1:3) 0.28; IR (ATR) ν_max_ 3280, 2928, 2855, 1671, 1556, 1457, 1376, 1289, 1199, 1177, 1130, 1044, 883, 834, 798, 749, 720 cm^−1^; ^1^H NMR (CD_3_OD, 400 MHz) δ 7.75 (2H, d, *J* = 1.8 Hz, H-4), 7.30 (2H, d, *J* = 8.7 Hz, H-7), 7.24 (2H, s, H-2), 7.21 (2H, dd, *J* = 8.5, 1.9 Hz, H-6), 3.65 (4H, s, H_2_-8), 3.31–3.27 (4H, m, H_2_-11), 2.83–2.76 (8H, m, H_2_-13 and H_2_-15), 1.81 (4H, tt, *J* = 6.8, 6.8 Hz, H_2_-12), 1.59–1.56 (4H, m, H_2_-16), 1.38–1.30 (12H, m, H_2_-17, H_2_-18 and H_2_-19); ^13^C NMR (CD_3_OD, 100 MHz) δ 176.0 (C-9), 136.9 (C-7a), 130.2 (C-3a), 126.7 (C-2), 125.4 (C-6), 122.1 (C-4), 114.3 (C-7), 113.2 (C-5), 109.4 (C-3), 49.1 (C-15), 46.0 (C-13), 36.7 (C-11), 33.8 (C-8), 30.4 (C-19), 30.2 (C-18), 27.7 (C-12), 27.5 (C-17), 27.3 (C-16); (+)-HRESIMS [M + H]^+^ *m/z* 757.2427 (calcd for C_36_H_51_^79^Br_2_N_6_O_2_, 757.2435), 759.2409 (calcd for C_36_H_51_^79^Br^81^BrN_6_O_2_, 759.2418), 761.2390 (calcd for C_36_H_51_^81^Br_2_N_6_O_2_, 761.2406).

#### 2.2.10. *N*^1^,*N*^12^-Bis(3-(2-(5-bromo-1*H*-indol-3-yl)acetamido)propyl)dodecane-1,12-diaminium 2,2,2-trifluoroacetate (**15f**)

Following general procedure A, 5-bromoindole-3-acetic acid (**7**) (0.050 g, 0.197 mmol) was reacted with EDC**·**HCl (0.045 g, 0.233 mmol), HOBt (0.031 g, 0.233 mmol), DIPEA (0.09 mL, 0.537 mmol) and di-*tert*-butyl dodecane-1,12-diylbis((3-aminopropyl)carbamate) (**13f**) (0.046 g, 0.0894 mmol) to afford di-*tert*-butyl dodecane-1,12-diylbis((3-(2-(5-bromo-1*H*-indol-3-yl)acetamido)propyl)carbamate) (0.029 g, 33%) as a clear colorless oil. Following general procedure B, a sub-sample of this product (0.022 g, 0.018 mmol) was treated with TFA/CH_2_Cl_2_. The crude product was purified with C_8_ reversed-phase flash column chromatography (50% MeOH/H_2_O (0.05% TFA)) affording the di-TFA salt **15f** (0.018 g, 97%) as an orange oil. R*_f_* (RP-18, 10% *aq* HCl:MeOH 1:3) 0.25; IR (ATR) ν_max_ 3268, 2928, 2855, 1671, 1656, 1457, 1376, 1289, 1200, 1178, 1130, 1044, 883, 834, 798, 749, 721 cm^−1^; ^1^H NMR (CD_3_OD, 400 MHz) δ 7.75 (2H, d, *J* = 1.8 Hz, H-4), 7.30 (2H, d, *J* = 8.4 Hz, H-7), 7.24 (2H, s, H-2), 7.21 (2H, dd, *J* = 8.6, 1.9 Hz, H-6), 3.65 (4H, s, H_2_-8), 3.31 (4H, obscured by solvent, H_2_-11), 2.83–2.76 (8H, m, H_2_-13 and H_2_-15), 1.80 (4H, tt, *J* = 6.8, 6.7 Hz, H_2_-12), 1.57 (4H, tt, *J* = 7.0, 6.9 Hz, H_2_-16), 1.36–1.29 (16H, m, H_2_-17, H_2_-18, H_2_-19 and H_2_-20); ^13^C NMR (CD_3_OD, 100 MHz) δ 176.0 (C-9), 136.9 (C-7a), 130.2 (C-3a), 126.7 (C-2), 125.4 (C-6), 122.1 (C-4), 114.2 (C-7), 113.2 (C-5), 109.4 (C-3), 49.1 (C-15), 46.0 (C-13), 36.7 (C-11), 33.8 (C-8), 30.6 (C-20), 30.5 (C-19), 30.2 (C-18), 27.7 (C-12), 27.5 (C-17), 27.3 (C-16); (+)-HRESIMS [M + H]^+^ *m/z* 785.2732 (calcd for C_38_H_55_^79^Br_2_N_6_O_2_, 785.2748), 787.2702 (calcd for C_38_H_55_^79^Br^81^BrN_6_O_2_, 787.2731), 789.2703 (calcd for C_38_H_55_^81^Br_2_N_6_O_2_, 789.2720).

#### 2.2.11. *N*^1^,*N*^6^-Bis(3-(2-(5-methoxy-1*H*-indol-3-yl)acetamido)propyl)hexane-1,6-diaminium 2,2,2-trifluoroacetate (**16b**)

Following general procedure A, 5-methoxyindole-3-acetic acid (**8**) (0.052 g, 0.256 mmol) was reacted with EDC**·**HCl (0.058 g, 0.302 mmol), HOBt (0.041 g, 0.302 mmol), DIPEA (0.12 mL, 0.69 mmol) and di-*tert*-butyl hexane-1,6-diylbis((3-aminopropyl)carbamate) (**13b**) (0.050 g, 0.116 mmol) to afford di-*tert*-butyl hexane-1,6-diylbis((3-(2-(5-methoxy-1*H*-indol-3-yl)acetamido)propyl)carbamate) (0.046 g, 51%) as a colorless oil. Following general procedure B, a sub-sample of this product (0.031 g, 0.039 mmol) was reacted with TFA in CH_2_Cl_2_ to afford, after chromatography, the di-TFA salt **16b** (0.024 g, 75%) as a dark purple gum. R*_f_* (RP-18, 10% *aq* HCl:MeOH 3:7) 0.65; IR (ATR) ν_max_ 3289, 2944, 1675, 1488, 1202, 1134, 1059, 835, 800, 722 cm^−1^; ^1^H NMR (CD_3_OD, 400 MHz) δ 7.26 (2H, d, *J* = 8.9 Hz, H-7), 7.17 (2H, s, H-2), 7.08 (2H, d, *J* = 2.4 Hz, H-4), 6.79 (2H, dd, *J* = 8.8, 2.4 Hz, H-6), 3.81 (6H, s, OMe), 3.65 (4H, s, H_2_-8), 3.28 (4H, t, *J* = 6.8 Hz, H_2_-11), 2.78 (4H, t, *J* = 7.2 Hz, H_2_-13), 2.70 (4H, t, *J* = 7.8 Hz, H_2_-15), 1.80 (4H, tt, *J* = 7.2, 6.8 Hz, H_2_-12), 1.57–1.48 (4H, m, H_2_-16), 1.30–1.24 (4H, m, H_2_-17); ^13^C NMR (CD_3_OD, 100 MHz) δ 176.4 (C-9), 155.2 (C-5), 133.4 (C-7a), 128.8 (C-3a), 125.9 (C-2), 113.2 (C-7), 112.8 (C-6), 109.3 (C-3), 101.6 (C-4), 56.4 (OMe), 48.7 (C-15), 45.9 (C-13), 36.7 (C-11), 34.0 (C-8), 27.6 (C-12), 26.9 (C-16/C-17), 26.8 (C-16/C-17); (+)-HRESIMS [M + Na]^+^ *m/z* 627.3643 (calcd C_34_H_48_N_6_O_4_Na, 627.3629).

#### 2.2.12. *N*^1^,*N*^7^-Bis(3-(2-(5-methoxy-1*H*-indol-3-yl)acetamido)propyl)heptane-1,7-diaminium 2,2,2-trifluoroacetate (**16c**)

Following general procedure A, 5-methoxyindole-3-acetic acid (**8**) (0.051 g, 0.248 mmol) was reacted with EDC**·**HCl (0.056 g, 0.293 mmol), HOBt (0.040 g, 0.293 mmol), DIPEA (0.12 mL, 0.677 mmol) and di-*tert*-butyl heptane-1,7-diylbis((3-aminopropyl)carbamate) (**13c**) (0.050 g, 0.113 mmol) to afford di-*tert*-butyl heptane-1,7-diylbis((3-(2-(5-methoxy-1*H*-indol-3-yl)acetamido)propyl)carbamate) (0.048 g, 52%) as a colorless oil. Following general procedure B, a sub-sample of this product (0.033 g, 0.040 mmol) was deprotected to afford the di-TFA salt **16c** (0.033 g, 97%) as a dark purple gum. R*_f_* (RP-18, 10% *aq* HCl:MeOH 3:7) 0.65; IR (ATR) ν_max_ 3283, 2941, 1675, 1486, 1202,1180, 1134, 1059, 1027, 835, 800, 721 cm^−1^; ^1^H NMR (CD_3_OD, 400 MHz) δ 7.26 (2H, d, *J* = 8.9 Hz, H-7), 7.17 (2H, s, H-2), 7.08 (2H, d, *J* = 2.4 Hz, H-4), 6.79 (2H, dd, *J* = 8.8, 2.4 Hz, H-6), 3.81 (6H, s, OMe), 3.65 (4H, s, H_2_-8), 3.29 (4H, t, *J* = 6.3 Hz, H_2_-11), 2.77 (4H, t, *J* = 7.2 Hz, H_2_-13), 2.70 (4H, t, *J* = 7.7 Hz, H_2_-15), 1.80 (4H, tt, *J* = 7.2, 6.3 Hz, H_2_-12), 1.58–1.49 (4H, m, H_2_-16), 1.32–1.26 (6H, m, H_2_-17, H_2_-18); ^13^C NMR (CD_3_OD, 100 MHz) δ 176.4 (C-9), 155.2 (C-5), 133.4 (C-7a), 128.7 (C-3a), 125.9 (C-2), 113.2 (C-7), 112.8 (C-6), 109.2 (C-3), 101.6 (C-4), 56.4 (OMe), 49.0 (C-15), 46.0 (C-13), 36.7 (C-11), 34.1 (C-8), 29.4 (C-18), 27.6 (C-12), 27.1 (C-16/C-17), 27.0 (C-16/C-17); (+)-HRESIMS [M + H]^+^ *m/z* 619.3965 (calcd for C_35_H_51_N_6_O_4_, 619.3966).

#### 2.2.13. *N*^1^,*N*^8^-Bis(3-(2-(5-methoxy-1*H*-indol-3-yl)acetamido)propyl)octane-1,8-diaminium 2,2,2-trifluoroacetate (**16d**)

Following general procedure A, 5-methoxyindole-3-acetic acid (**8**) (0.049 g, 0.239 mmol) was reacted with EDC**·**HCl (0.054 g, 0.283 mmol), HOBt (0.038 g, 0.283 mmol), DIPEA (0.11 mL, 0.654 mmol) and di-*tert*-butyl octane-1,8-diylbis((3-aminopropyl)carbamate) (**13d**) (0.050 g, 0.110 mmol), to afford di-*tert*-butyl octane-1,8-diylbis((3-(2-(5-methoxy-1*H*-indol-3-yl)acetamido)propyl)carbamate (0.069 g, 59%) as a colorless oil. Following general procedure B, a sub-sample of this product (0.054 g, 0.065 mmol) was reacted with TFA in CH_2_Cl_2_ to afford, after chromatography, the di-TFA salt **16d** (0.054 g, 97%) as a dark purple gum. R*_f_* (RP-18, 10% *aq* HCl:MeOH 3:7) 0.65; IR (ATR) ν_max_ 3288, 2939, 2859, 1675, 1489, 1202, 1180, 1134, 1059, 1028, 834, 800, 721 cm^−1^; ^1^H NMR (CD_3_OD, 400 MHz) δ 7.26 (2H, d, *J* = 8.9 Hz, H-7), 7.17 (2H, s, H-2), 7.07 (2H, d, *J* = 2.4 Hz, H-4), 6.79 (2H, dd, *J* = 8.8, 2.4 Hz, H-6), 3.80 (6H, s, OMe), 3.64 (4H, s, H_2_-8), 3.28 (4H, t, *J* = 6.4 Hz, H_2_-11), 2.76 (4H, t, *J* = 7.3 Hz, H_2_-13), 2.68 (4H, t, *J* = 7.8 Hz, H_2_-15), 1.79 (4H, tt, *J* = 7.3, 6.4 Hz, H_2_-12), 1.57–1.49 (4H, m, H_2_-16), 1.32–1.25 (8H, m, H_2_-17, H_2_-18); ^13^C NMR (CD_3_OD, 100 MHz) δ 176.4 (C-9), 155.2 (C-5), 133.4 (C-7a), 128.7 (C-3a), 125.9 (C-2), 113.2 (C-7), 112.7 (C-6), 109.2 (C-3), 101.6 (C-4), 56.4 (OMe), 48.9 (C-15), 46.0 (C-13), 36.7 (C-11), 34.1 (C-8), 29.8 (C-18), 27.6 (C-12), 27.3 (C-16/C-17), 27.1 (C-16/C-17); (+)-HRESIMS [M + H]^+^ *m/z* 633.4125 (calcd for C_36_H_53_N_6_O_4_, 633.4123).

#### 2.2.14. *N*^1^,*N*^10^-Bis(3-(2-(5-methoxy-1*H*-indol-3-yl)acetamido)propyl)decane-1,10-diaminium 2,2,2-trifluoroacetate (**16e**)

Following general procedure A, 5-methoxyindole-3-acetic acid (**8**) (0.050 g, 0.244 mmol) was reacted with EDC**·**HCl (0.055 g, 0.288 mmol), HOBt (0.039 g, 0.288 mmol), DIPEA (0.12 mL, 0.665 mmol) and di-*tert*-butyl decane-1,10-diylbis((3-aminopropyl)carbamate) (**13e**) (0.054 g, 0.111 mmol) to yield di-*tert*-butyl decane-1,10-diylbis((3-(2-(5-methoxy-1*H*-indol-3-yl)acetamido)propyl)carbamate) (0.045 g, 47%) as a pale yellow oil. Following general procedure B, a sub-sample of this product (0.027 g, 0.031 mmol) was reacted with TFA in CH_2_Cl_2_ to afford, after chromatography, the di-TFA salt **16e** (0.020 g, 72%) as a brown oil. R*_f_* (RP-18, 10% *aq* HCl:MeOH 1:3) 0.50; IR (ATR) ν_max_ 3283, 2935, 2857, 1672, 1488, 1440, 1303, 1201, 1181, 1134, 1059, 1027, 917, 836, 801, 722 cm^−1^; ^1^H NMR (CD_3_OD, 400 MHz) δ 7.26 (2H, d, *J* = 8.8 Hz, H-7), 7.18 (2H, s, H-2), 7.08 (2H, d, *J* = 2.4 Hz, H-4), 6.79 (2H, dd, *J* = 8.8, 2.4 Hz, H-6), 3.82 (6H, s, OMe), 3.64 (4H, s, H_2_-8), 3.29 (4H, t, *J* = 6.8 Hz, H_2_-11), 2.77 (4H, t, *J* = 7.2 Hz, H_2_-13), 2.70 (4H, t, *J* = 7.8 Hz, H_2_-15), 1.79 (4H, tt, *J* = 6.8, 6.8 Hz, H_2_-12), 1.57–1.51 (4H, m, H_2_-16), 1.38–1.28 (12H, m, H_2_-17, H_2_-18 and H_2_-19); ^13^C NMR (CD_3_OD, 100 MHz) δ 176.4 (C-9), 155.3 (C-5), 133.4 (C-7a), 128.7 (C-3a), 125.8 (C-2), 113.2 (C-7), 112.7 (C-6), 109.2 (C-3), 101.6 (C-4), 56.4 (OMe), 48.8 (C-15), 46.0 (C-13), 36.7 (C-11), 34.1 (C-8), 30.3 (C-19), 30.1 (C-18), 27.6 (C-12), 27.4 (C-17), 27.2 (C-16); (+)-HRESIMS [M + H]^+^ *m/z* 661.4433 (calcd for C_38_H_57_N_6_O_4_, 661.4436).

#### 2.2.15. *N*^1^,*N*^12^-Bis(3-(2-(5-methoxy-1*H*-indol-3-yl)acetamido)propyl)dodecane-1,12-diaminium 2,2,2-trifluoroacetate (**16f**)

Following general procedure A, 5-methoxyindole-3-acetic acid (**8**) (0.050 g, 0.244 mmol) was reacted with EDC**·**HCl (0.055 g, 0.288 mmol), HOBt (0.039 g, 0.288 mmol), DIPEA (0.12 mL, 0.665 mmol) and di-*tert*-butyl dodecane-1,12-diylbis((3-aminopropyl)carbamate) (**13f**) (0.057 g, 0.111 mmol) to afford di-*tert*-butyl dodecane-1,12-diylbis((3-(2-(5-methoxy-1*H*-indol-3-yl)acetamido)propyl)carbamate) (0.048 g, 49%) as a clear colorless oil. Following general procedure B, a sub-sample of this product (0.040 g, 0.045 mmol) was reacted with TFA in CH_2_Cl_2_ to afford, after chromatography, the di-TFA salt **16f** (0.007 g, 17%) as a yellow oil. R*_f_* (RP-18, 10% *aq* HCl:MeOH 1:3) 0.47; IR (ATR) ν_max_ 3286, 2936, 2857, 1672, 1488, 1440, 1303, 1201, 1181, 1134, 1059, 1027, 918, 836, 801, 722 cm^−1^; ^1^H NMR (CD_3_OD, 400 MHz) δ 7.26 (2H, d, *J* = 8.8 Hz, H-7), 7.18 (2H, s, H-2), 7.08 (2H, d, *J* = 2.4 Hz, H-4), 6.79 (2H, dd, *J* = 8.8, 2.4 Hz, H-6), 3.82 (6H, s, OMe), 3.65 (4H, s, H_2_-8), 3.30–3.28 (4H, m, H_2_-11), 2.77 (4H, t, *J* = 7.2 Hz, H_2_-13), 2.70 (4H, t, *J* = 7.9 Hz, H_2_-15), 1.79 (4H, tt, *J* = 6.7, 6.7 Hz, H_2_-12), 1.57–1.50 (4H, m, H_2_-16), 1.38–1.28 (16H, m, H_2_-17, H_2_-18, H_2_-19 and H_2_-20); ^13^C NMR (CD_3_OD, 100 MHz) δ 176.5 (C-9), 155.3 (C-5), 133.4 (C-7a), 128.7 (C-3a), 125.9 (C-2), 113.2 (C-7), 112.7 (C-6), 109.4 (C-3), 101.7 (C-4), 56.4 (OMe), 48.8 (C-15), 46.0 (C-13), 36.7 (C-11), 34.1 (C-8), 30.6 (C-20), 30.5 (C-19), 30.2 (C-18), 27.7 (C-12), 27.5 (C-17), 27.2 (C-16); (+)-HRESIMS [M + H]^+^ *m/z* 689.4744 (calcd for C_40_H_61_N_6_O_4_, 689.4749).

#### 2.2.16. *N*^1^,*N*^4^-Bis(3-(2-(5-methyl-1*H*-indol-3-yl)acetamido)propyl)butane-1,4-diaminium 2,2,2-trifluoroacetate (**17a**)

Following general procedure A, 5-methylindole-3-acetic acid (**9**) (0.050 g, 0.264 mmol) was reacted with EDC**·**HCl (0.060 g, 0.312 mmol), HOBt (0.042 g, 0.312 mmol), DIPEA (0.13 mL, 0.721 mmol) and di-*tert*-butyl butane-1,4-diylbis((3-aminopropyl)carbamate) (**13a**) (0.048 g, 0.120 mmol) to afford di-*tert*-butyl butane-1,4-diylbis((3-(2-(5-methyl-1*H*-indol-3-yl)acetamido)propyl)carbamate) (0.068 g, 76%) as a clear colorless oil. Following general procedure B, a sub-sample of this product (0.010 g, 0.013 mmol) was reacted with TFA in CH_2_Cl_2_ to afford, after chromatography, the di-TFA salt **17a** (0.009 g, 87%) as a yellow oil. R*_f_* (RP-18, 10% *aq* HCl:MeOH 1:3) 0.80; IR (ATR) ν_max_ 3281, 3033, 2923, 2853, 1670, 1556, 1471, 1431, 1199, 1177, 1127, 834, 798, 720 cm^−1^; ^1^H NMR (CD_3_OD, 400 MHz) δ 7.34 (2H, s, H-4), 7.25 (2H, d, *J* = 8.4 Hz, H-7), 7.14 (2H, s, H-2), 6.96 (2H, dd, *J* = 8.2, 1.5 Hz, H-6), 3.67 (4H, s, H_2_-8), 3.31 (4H, obscured by solvent, H_2_-11), 2.81 (4H, t, *J* = 7.0 Hz, H_2_-13), 2.72 (4H, t, *J* = 6.6 Hz, H_2_-15), 2.41 (6H, s, Me), 1.81 (4H, tt, *J* = 6.6, 6.6 Hz, H_2_-12), 1.54 (4H, tt, *J* = 3.6, 3.6 Hz, H_2_-16); ^13^C NMR (CD_3_OD, 100 MHz) δ 176.7 (C-9), 136.5 (C-7a), 129.2 (C-5), 128.7 (C-3a), 125.3 (C-2), 124.4 (C-6), 118.9 (C-4), 112.3 (C-7), 108.8 (C-3), 47.9 (C-15), 45.9 (C-13), 36.6 (C-11), 33.9 (C-8), 27.7 (C-12), 24.0 (C-16), 21.7 (Me); (+)-HRESIMS [M + H]^+^ *m/z* 545.3600 (calcd for C_32_H_45_N_6_O_2_, 545.3599).

#### 2.2.17. *N*^1^,*N*^6^-Bis(3-(2-(5-methyl-1*H*-indol-3-yl)acetamido)propyl)hexane-1,6-diaminium 2,2,2-trifluoroacetate (**17b**)

Following general procedure A, 5-methylindole-3-acetic acid (**9**) (0.050 g, 0.264 mmol) was reacted with EDC**·**HCl (0.060 g, 0.312 mmol), HOBt (0.042 g, 0.312 mmol), DIPEA (0.13 mL, 0.721 mmol) and di-*tert*-butyl hexane-1,6-diylbis((3-aminopropyl)carbamate) (**13b**) (0.052 g, 0.120 mmol) to yield di-*tert*-butyl hexane-1,6-diylbis((3-(2-(5-methyl-1*H*-indol-3-yl)acetamido)propyl)carbamate) (0.068 g, 73%) as a clear colorless oil. Following general procedure B, a sub-sample of this product (0.045 g, 0.085 mmol) was reacted with TFA in CH_2_Cl_2_ to afford, after chromatography, the di-TFA salt **17b** (0.024 g, 51%) as a brown oil. R*_f_* (RP-18, 10% *aq* HCl:MeOH 1:3) 0.73; IR (ATR) ν_max_ 3282, 3033, 2923, 2853, 1670, 1556, 1470, 1432, 1199, 1178, 1127, 834, 798, 720 cm^−1^; ^1^H NMR (CD_3_OD, 400 MHz) δ 7.35 (2H, s, H-4), 7.25 (2H, d, *J* = 8.4 Hz, H-7), 7.15 (2H, s, H-2), 6.95 (2H, dd, *J* = 8.3, 1.4 Hz, H-6), 3.67 (4H, s, H_2_-8), 3.29 (4H, t, *J* = 6.4 Hz, H_2_-11), 2.79 (4H, t, *J* = 7.1 Hz, H_2_-13), 2.72 (4H, t, *J* = 7.8 Hz, H_2_-15), 2.41 (6H, s, Me), 1.80 (4H, tt, *J* = 6.7, 6.7 Hz, H_2_-12), 1.57–1.49 (4H, m, H_2_-16), 1.28 (4H, tt, *J* = 3.6, 3.6 Hz, H_2_-17); ^13^C NMR (CD_3_OD, 100 MHz) δ 176.5 (C-9), 136.5 (C-7a), 129.2 (C-5), 128.6 (C-3a), 125.2 (C-2), 124.3 (C-6), 118.9 (C-4), 112.3 (C-7), 108.9 (C-3), 48.5 (C-15, obscured by solvent), 45.9 (C-13), 36.7 (C-11), 33.9 (C-8), 27.6 (C-12), 26.82 (C-17), 26.77 (C-16), 21.7 (Me); (+)-HRESIMS [M + H]^+^ *m/z* 573.3903 (calcd for C_34_H_49_N_6_O_2_, 573.3912).

#### 2.2.18. *N*^1^,*N*^7^-Bis(3-(2-(5-methyl-1*H*-indol-3-yl)acetamido)propyl)heptane-1,7-diaminium 2,2,2-trifluoroacetate (**17c**)

Following general procedure A, 5-methylindole-3-acetic acid (**9**) (0.050 g, 0.264 mmol) was reacted with EDC**·**HCl (0.060 g, 0.312 mmol), HOBt (0.042 g, 0.312 mmol), DIPEA (0.13 mL, 0.721 mmol) and di-*tert*-butyl heptane-1,7-diylbis((3-aminopropyl)carbamate) (**13c**) (0.053 g, 0.120 mmol) to afford di-*tert*-butyl heptane-1,7-diylbis((3-(2-(5-methyl-1*H*-indol-3-yl)acetamido)propyl)carbamate) (0.059 g, 62%) as a clear colorless oil. Following general procedure B, a sub-sample of this product (0.039 g, 0.050 mmol) was reacted with TFA in CH_2_Cl_2_ to afford, after chromatography, the di-TFA salt **17c** (0.038 g, 94%) as a dark brown oil. R*_f_* (RP-18, 10% *aq* HCl:MeOH 1:3) 0.73; IR (ATR) ν_max_ 3285, 3036, 2924, 2853, 1670, 1556, 1469, 1431, 1199, 1177, 1127, 834, 798, 720 cm^−1^; ^1^H NMR (CD_3_OD, 400 MHz) δ 7.38 (2H, s, H-4), 7.25 (2H, d, *J* = 8.3 Hz, H-7), 7.15 (2H, s, H-2), 6.95 (2H, dd, *J* = 8.3, 1.3 Hz, H-6), 3.65 (4H, s, H_2_-8), 3.28 (4H, t, *J* = 6.4 Hz, H_2_-11), 2.78 (4H, t, *J* = 7.2 Hz, H_2_-13), 2.71 (4H, t, *J* = 7.2 Hz, H_2_-15), 2.41 (6H, s, Me), 1.79 (4H, tt, *J* = 6.7, 6.7 Hz, H_2_-12), 1.57–1.50 (4H, m, H_2_-16), 1.34–1.27 (6H, m, H_2_-17); ^13^C NMR (CD_3_OD, 100 MHz) δ 176.4 (C-9), 136.5 (C-7a), 129.1 (C-5), 128.6 (C-3a), 125.2 (C-2), 124.3 (C-6), 118.9 (C-4), 112.3 (C-7), 108.9 (C-3), 48.7 (C-15, obscured by solvent), 45.9 (C-13), 36.7 (C-11), 34.0 (C-8), 29.5 (C-18), 27.5 (C-12), 27.1 (C-17), 27.0 (C-16), 21.7 (Me); (+)-HRESIMS [M + H]^+^ *m/z* 587.4076 (calcd for C_35_H_51_N_6_O_2_, 587.4068).

#### 2.2.19. *N*^1^,*N*^8^-Bis(3-(2-(5-methyl-1*H*-indol-3-yl)acetamido)propyl)octane-1,8-diaminium 2,2,2-trifluoroacetate (**17d**)

Following general procedure A, 5-methylindole-3-acetic acid (**9**) (0.050 g, 0.264 mmol) was reacted with EDC**·**HCl (0.060 g, 0.312 mmol), HOBt (0.042 g, 0.312 mmol), DIPEA (0.13 mL, 0.721 mmol) and di-*tert*-butyl octane-1,8-diylbis((3-aminopropyl)carbamate) (**13d**) (0.55 g, 0.120 mmol) to yield di-*tert*-butyl octane-1,8-diylbis((3-(2-(5-methyl-1*H*-indol-3-yl)acetamido)propyl)carbamate) (0.082 g, 85%) as a clear colorless oil. Following general procedure B, a sub-sample of this product (0.041 g, 0.051 mmol) was reacted with TFA in CH_2_Cl_2_ to afford, after chromatography, the di-TFA salt **17d** (0.007 g, 17%) as a yellow oil. R*_f_* (RP-18, 10% *aq* HCl:MeOH 1:3) 0.70; IR (ATR) ν_max_ 3278, 2925, 2857, 1670, 1556, 1471, 1432, 1198, 1177, 1127, 834, 798, 720 cm^−1^; ^1^H NMR (CD_3_OD, 400 MHz) δ 7.35 (2H, s, H-4), 7.25 (2H, d, *J* = 8.3 Hz, H-7), 7.16 (2H, s, H-2), 6.96 (2H, dd, *J* = 8.3, 2.0 Hz, H-6), 3.66 (4H, s, H_2_-8), 3.29 (4H, t, *J* = 6.4 Hz, H_2_-11), 2.79 (4H, t, *J* = 7.1 Hz, H_2_-13), 2.72 (4H, t, *J* = 7.8 Hz, H_2_-15), 2.41 (6H, s, Me), 1.80 (4H, tt, *J* = 6.7, 6.5 Hz, H_2_-12), 1.58–1.51 (4H, m, H_2_-16), 1.37–1.30 (8H, m, H_2_-17 and H_2_-18); ^13^C NMR (CD_3_OD, 100 MHz) δ 176.6 (C-9), 136.6 (C-7a), 129.2 (C-5), 128.6 (C-3a), 125.2 (C-2), 124.3 (C-6), 118.9 (C-4), 112.3 (C-7), 108.9 (C-3), 48.9 (C-15), 45.9 (C-13), 36.7 (C-11), 34.0 (C-8), 29.9 (C-19), 27.6 (C-12), 27.3 (C-18), 27.2 (C-17), 27.1 (C-16), 21.7 (Me); (+)-HRESIMS [M + H]^+^ *m/z* 601.4225 (calcd for C_36_H_53_N_6_O_2_, 601.4225).

#### 2.2.20. *N*^1^,*N*^10^-Bis(3-(2-(5-methyl-1*H*-indol-3-yl)acetamido)propyl)decane-1,10-diaminium 2,2,2-trifluoroacetate (**17e**)

Following general procedure A, 5-methylindole-3-acetic acid (**9**) (0.050 g, 0.264 mmol) was reacted with EDC**·**HCl (0.060 g, 0.312 mmol), HOBt (0.042 g, 0.312 mmol), DIPEA (0.13 mL, 0.721 mmol) and di-*tert*-butyl decane-1,10-diylbis((3-aminopropyl)carbamate) (**13e**) (0.058 g, 0.120 mmol) to yield di-*tert*-butyl decane-1,10-diylbis((3-(2-(5-methyl-1*H*-indol-3-yl)acetamido)propyl)carbamate) (0.042 g, 42%) as a yellow oil. Following general procedure B, a sub-sample of this product (0.018 g, 0.022 mmol) was reacted with TFA in CH_2_Cl_2_ to afford, after chromatography, the di-TFA salt **17e** (0.014 g, 75%) as a pale yellow oil. R*_f_* (RP-18, 10% *aq* HCl:MeOH 1:3) 0.67; IR (ATR) ν_max_ 3279, 2925, 2855, 1670, 1556, 1431, 1199, 1177, 1127, 834, 798, 720 cm^−1^; ^1^H NMR (CD_3_OD, 400 MHz) δ 7.36 (2H, s, H-4), 7.25 (2H, d, *J* = 8.2 Hz, H-7), 7.16 (2H, s, H-2), 6.95 (2H, dd, *J* = 8.2, 1.4 Hz, H-6), 3.65 (4H, s, H_2_-8), 3.29 (4H, t, *J* = 6.5 Hz, H_2_-11), 2.78 (4H, t, *J* = 6.9 Hz, H_2_-13), 2.71 (4H, t, *J* = 7.8 Hz, H_2_-15), 2.41 (6H, s, Me), 1.79 (4H, tt, *J* = 6.8, 6.7 Hz, H_2_-12), 1.54 (4H, tt, *J* = 7.5, 7.5 Hz, H_2_-16), 1.38–1.26 (12H, m, H_2_-17, H_2_-18 and H_2_-19); ^13^C NMR (CD_3_OD, 100 MHz) δ 176.5 (C-9), 136.6 (C-7a), 129.1 (C-5), 128.6 (C-3a), 125.2 (C-2), 124.3 (C-6), 118.9 (C-4), 112.3 (C-7), 108.9 (C-3), 48.7 (C-15, obscured by solvent), 45.9 (C-13), 36.7 (C-11), 34.0 (C-8), 30.3 (C-19), 30.1 (C-18), 27.6 (C-12), 27.5 (C-17), 27.2 (C-16), 21.7 (Me); (+)-HRESIMS [M + H]^+^ *m/z* 629.4537 (calcd for C_38_H_57_N_6_O_2_, 629.4538).

#### 2.2.21. *N*^1^,*N*^12^-Bis(3-(2-(5-methyl-1*H*-indol-3-yl)acetamido)propyl)dodecane-1,12-diaminium 2,2,2-trifluoroacetate (**17f**)

Following general procedure A, 5-methylindole-3-acetic acid (**9**) (0.050 g, 0.264 mmol) was reacted with EDC**·**HCl (0.060 g, 0.312 mmol), HOBt (0.042 g, 0.312 mmol), DIPEA (0.13 mL, 0.721 mmol) and di-*tert*-butyl dodecane-1,12-diylbis((3-aminopropyl)carbamate) (**13f**) (0.062 g, 0.120 mmol) to afford di-*tert*-butyl dodecane-1,12-diylbis((3-(2-(5-methyl-1*H*-indol-3-yl)acetamido)propyl)carbamate) (0.082 g, 80%) as a pale yellow oil. Following general procedure B, a sub-sample of this product (0.041 g, 0.048 mmol) was reacted with TFA in CH_2_Cl_2_ to afford, after chromatography, the di-TFA salt **17f** (0.036 g, 85%) as a brown oil. R*_f_* (RP-18, 10% *aq* HCl:MeOH 1:3) 0.60; IR (ATR) ν_max_ 3282, 2925, 2855, 1670, 1655, 1471, 1431, 1199, 1177, 1127, 834, 798, 720 cm^−1^; ^1^H NMR (CD_3_OD, 400 MHz) δ 7.36 (2H, s, H-4), 7.23 (2H, d, *J* = 8.3 Hz, H-7), 7.16 (2H, s, H-2), 6.96 (2H, d, *J* = 8.3 Hz, H-6), 3.65 (4H, s, H_2_-8), 3.29 (4H, t, *J* = 6.6 Hz, H_2_-11), 2.78 (4H, t, *J* = 7.2 Hz, H_2_-13), 2.71 (4H, t, *J* = 7.9 Hz, H_2_-15), 2.41 (6H, s, Me), 1.79 (4H, tt, *J* = 6.7, 6.6 Hz, H_2_-12), 1.58–1.50 (4H, m, H_2_-16), 1.39–1.28 (16H, m, H_2_-17, H_2_-18, H_2_-19 and H_2_-20); ^13^C NMR (CD_3_OD, 100 MHz) δ 176.6 (C-9), 136.6 (C-7a), 129.1 (C-5), 128.6 (C-3a), 125.2 (C-2), 124.3 (C-6), 118.9 (C-4), 112.3 (C-7), 108.9 (C-3), 48.6 (C-15, obscured by solvent), 45.8 (C-13), 36.7 (C-11), 34.0 (C-8), 27.6 (C-12), 30.6 (C-20), 30.5 (C-19), 30.2 (C-18), 27.5 (C-17), 27.2 (C-16), 21.7 (Me); (+)-HRESIMS [M + H]^+^ *m/z* 657.4844 (calcd for C_40_H_61_N_6_O_2_, 657.4851).

#### 2.2.22. *N*^1^,*N*^4^-Bis(3-(2-(7-fluoro-1*H*-indol-3-yl)acetamido)propyl)butane-1,4-diaminium 2,2,2-trifluoroacetate (**18a**)

Following general procedure A, 7-fluoroindole-3-acetic acid (**10**) (0.040 g, 0.207 mmol) was reacted with EDC**·**HCl (0.047 g, 0.245 mmol), HOBt (0.033 g, 0.245 mmol), DIPEA (0.10 mL, 0.565 mmol) and di-*tert*-butyl butane-1,4-diylbis((3-aminopropyl)carbamate) (**13a**) (0.038 g, 0.094 mmol) to afford di-*tert*-butyl butane-1,4-diylbis((3-(2-(7-fluoro-1*H*-indol-3-yl)acetamido)propyl)carbamate) (0.047 g, 66%) as a pale yellow oil. Following general procedure B, a sub-sample of this product (0.020 g, 0.027 mmol) was reacted with TFA in CH_2_Cl_2_ to afford, after chromatography, the di-TFA salt **18a** (0.019 g, 92%) as a red oil. R*_f_* (RP-18, 10% *aq* HCl:MeOH 1:3) 0.83; IR (ATR) ν_max_ 3263, 3081, 2829, 1669, 1643, 1581, 1433, 1365, 1199, 1179, 1127, 1048, 969, 835, 798, 721 cm^−1^; ^1^H NMR (CD_3_OD, 400 MHz) δ 7.36 (2H, d, *J* = 7.9 Hz, H-4), 7.25 (2H, s, H-2), 6.98 (2H, td, *J* = 7.9, 4.7 Hz, H-5), 6.85 (2H, dd, *J* = 11.5, 7.4 Hz, H-6), 3.69 (4H, s, H_2_-8), 3.30 (4H, obscured by solvent, H_2_-11), 2.82 (4H, t, *J* = 7.1 Hz, H_2_-13), 2.78 (4H, t, *J* = 6.7 Hz, H_2_-15), 1.82 (4H, tt, *J* = 6.7, 6.7 Hz, H_2_-12), 1.59 (4H, tt, *J* = 3.8, 3.7 Hz, H_2_-16); ^13^C NMR (CD_3_OD, 100 MHz) δ 176.1 (C-9), 151.2 (d, ^1^*J*_CF_ = 243.2 Hz, C-7), 132.5 (d, ^3^*J*_CF_ = 5.8 Hz, C-3a), 126.2 (C-2), 126.1 (d, ^2^*J*_CF_ = 15.8 Hz, C-7a), 120.4 (d, ^3^*J*_CF_ = 6.2 Hz, C-5), 115.5 (d, ^4^*J*_CF_ = 3.2 Hz, C-4), 110.5 (d, ^4^*J*_CF_ = 1.7 Hz, C-3), 107.3 (d, ^2^*J*_CF_ = 16.4 Hz, C-6), 48.0 (C-15), 46.1 (C-13), 36.8 (C-11), 33.8 (C-9), 27.7 (C-12), 24.1 (C-16); (+)-HRESIMS [M + H]^+^ *m/z* 553.3096 (calcd for C_30_H_39_F_2_N_6_O_2_, 553.3097).

#### 2.2.23. *N*^1^,*N*^6^-Bis(3-(2-(7-fluoro-1*H*-indol-3-yl)acetamido)propyl)hexane-1,6-diaminium 2,2,2-trifluoroacetate (**18b**)

Following general procedure A, 7-fluoroindole-3-acetic acid (**10**) (0.040 g, 0.207 mmol) was reacted with EDC**·**HCl (0.047 g, 0.245 mmol), HOBt (0.033 g, 0.245 mmol), DIPEA (0.10 mL, 0.565 mmol) and di-*tert*-butyl hexane-1,6-diylbis((3-aminopropyl)carbamate) (**13b**) (0.041 g, 0.094 mmol) to afford di-*tert*-butyl hexane-1,6-diylbis((3-(2-(7-fluoro-1*H*-indol-3-yl)acetamido)propyl)carbamate) (0.048 g, 65%) as a pale yellow oil. Following general procedure B, a sub-sample of this product (0.024 g, 0.031 mmol) was reacted with TFA in CH_2_Cl_2_ to afford, after chromatography, the di-TFA salt **18b** (0.007 g, 28%) as a red-brown oil. R*_f_* (RP-18, 10% *aq* HCl:MeOH 1:3) 0.67; IR (ATR) ν_max_ 3266, 3082, 2829, 1669, 1643, 1581, 1436, 1365, 1198, 1179, 1127, 1048, 969, 835, 798, 721 cm^−1^; ^1^H NMR (CD_3_OD, 400 MHz) δ 7.37 (2H, d, *J* = 8.0 Hz, H-4), 7.25 (2H, s, H-2), 6.98 (2H, td, *J* = 7.9, 4.7 Hz, H-5), 6.88–6.83 (2H, m, H-6), 3.69 (4H, s, H_2_-8), 3.31 (4H, obscured by solvent, H_2_-11), 2.82 (4H, t, *J* = 7.1 Hz, H_2_-13), 2.76 (4H, t, *J* = 7.8 Hz, H_2_-15), 1.81 (4H, tt, *J* = 6.7, 6.7 Hz, H_2_-12), 1.56–1.52 (4H, m, H_2_-16), 1.35–1.26 (4H, m, H_2_-17); ^13^C NMR (CD_3_OD, 100 MHz) δ 176.2 (C-9), 151.2 (d, ^1^*J*_CF_ = 242.3 Hz, C-7), 132.5 (C-3a), 126.5 (C-2), 126.5 (C-2 and C-7a, obscured by solvent), 120.3 (d, ^3^*J*_CF_ = 6.1 Hz, C-5), 115.4 (d, ^4^*J*_CF_ = 3.1 Hz, C-4), 110.5 (C-3), 107.3 (d, ^2^*J*_CF_ = 16.6 Hz, C-6), 48.7 (C-15), 46.0 (C-13), 36.7 (C-11), 33.8 (C-8), 27.7 (C-12), 26.9 (C-16 and C-17); (+)-HRESIMS [M + H]^+^ *m/z* 581.3409 (calcd for C_32_H_43_F_2_N_6_O_2_, 581.3410).

#### 2.2.24. *N*^1^,*N*^7^-Bis(3-(2-(7-fluoro-1*H*-indol-3-yl)acetamido)propyl)heptane-1,7-diaminium 2,2,2-trifluoroacetate (**18c**)

Following general procedure A, 7-fluoroindole-3-acetic acid (**10**) (0.040 g, 0.207 mmol) was reacted with EDC**·**HCl (0.047 g, 0.245 mmol), HOBt (0.033 g, 0.245 mmol), DIPEA (0.10 mL, 0.565 mmol) and di-*tert*-butyl heptane-1,7-diylbis((3-aminopropyl)carbamate) (**13c**) (0.042 g, 0.094 mmol) to afford di-*tert*-butyl heptane-1,7-diylbis((3-(2-(7-fluoro-1*H*-indol-3-yl)acetamido)propyl)carbamate) (0.029 g, 39%) as a pale yellow oil. Following general procedure B, a sub-sample of this product (0.015 g, 0.019 mmol) was reacted with TFA in CH_2_Cl_2_ to afford, after chromatography, the di-TFA salt **18c** (0.008 g, 52%) as a red oil. R*_f_* (RP-18, 10% *aq* HCl:MeOH 1:3) 0.62; IR (ATR) ν_max_ 3264, 3083, 2831, 1669, 1644, 1581, 1433, 1365, 1198, 1180, 1126, 1048, 969, 835, 798, 721 cm^−1^; ^1^H NMR (CD_3_OD, 400 MHz) δ 7.37 (2H, d, *J* = 7.9 Hz, H-4), 7.25 (2H, s, H-2), 6.98 (2H, td, *J* = 7.9, 4.8 Hz, H-5), 6.85 (2H, dd, *J* = 11.1, 7.8 Hz, H-6), 3.69 (4H, s, H_2_-8), 3.30 (4H, obscured by solvent, H_2_-11), 2.81 (4H, t, *J* = 7.1 Hz, H_2_-13), 2.77 (4H, t, *J* = 7.8 Hz, H_2_-15), 1.81 (4H, tt, *J* = 6.8, 6.7 Hz, H_2_-12), 1.55 (4H, tt, *J* = 7.2, 7.2 Hz, H_2_-16), 1.35–1.28 (6H, m, H_2_-17 and H_2_-18); ^13^C NMR (CD_3_OD, 100 MHz) δ 176.1 (C-9), 151.2 (d, ^1^*J*_CF_ = 243.0 Hz, C-7), 132.5 (d, ^3^*J*_CF_ = 5.8 Hz, C-3a), 126.2 (C-2), 126.1 (C-2 and C-7a, obscured by solvent), 120.3 (d, ^3^*J*_CF_ = 6.2 Hz, C-5), 115.4 (d, ^4^*J*_CF_ = 3.2 Hz, C-4), 110.5 (d, ^4^*J*_CF_ = 1.9 Hz, C-3), 107.3 (d, ^2^*J*_CF_ = 16.7 Hz, C-6), 48.0 (C-15, obscured by solvent), 46.0 (C-13), 36.7 (C-11), 33.8 (C-8), 29.5 (C-18), 27.7 (C-12), 27.2 (C-17), 27.0 (C-16); (+)-HRESIMS [M + H]^+^ *m/z* 595.3552 (calcd for C_33_H_45_F_2_N_6_O_2_, 595.3567).

#### 2.2.25. *N*^1^,*N*^8^-Bis(3-(2-(7-fluoro-1*H*-indol-3-yl)acetamido)propyl)octane-1,8-diaminium 2,2,2-trifluoroacetate (**18d**)

Following general procedure A, 7-fluoroindole-3-acetic acid (**10**) (0.040 g, 0.207 mmol) was reacted with EDC**·**HCl (0.047 g, 0.245 mmol), HOBt (0.033 g, 0.245 mmol), DIPEA (0.10 mL, 0.565 mmol) and di-*tert*-butyl octane-1,8-diylbis((3-aminopropyl)carbamate) (**13d**) (0.043 g, 0.094 mmol) to afford di-*tert*-butyl octane-1,8-diylbis((3-(2-(7-fluoro-1*H*-indol-3-yl)acetamido)propyl)carbamate) (0.025 g, 33%) as a yellow oil. Following general procedure B, a sub-sample of this product (0.013 g, 0.016 mmol) was reacted with TFA in CH_2_Cl_2_ to afford, after chromatography, the di-TFA salt **18d** (0.005 g, 37%) as a red oil. R*_f_* (RP-18, 10% *aq* HCl:MeOH 1:3) 0.60; IR (ATR) ν_max_ 3267, 3082, 2830, 1669, 1645, 1531, 1433, 1365, 1198, 1179, 1126, 1048, 969, 835, 798, 720 cm^−1^; ^1^H NMR (CD_3_OD, 400 MHz) δ 7.37 (2H, d, *J* = 8.1 Hz, H-4), 7.25 (2H, s, H-2), 6.98 (2H, td, *J* = 7.9, 4.5 Hz, H-5), 6.85 (2H, dd, *J* = 11.2, 7.8 Hz, H-6), 3.69 (4H, s, H_2_-8), 3.30 (4H, obscured by solvent, H_2_-11), 2.81 (4H, t, *J* = 7.1 Hz, H_2_-13), 2.77 (4H, t, *J* = 7.8 Hz, H_2_-15), 1.81 (4H, tt, *J* = 6.7, 6.7 Hz, H_2_-12), 1.54 (4H, tt, *J* = 7.3, 7.3 Hz, H_2_-16), 1.35–1.29 (8H, m, H_2_-17 and H_2_-18); ^13^C NMR (CD_3_OD, 100 MHz) δ 176.1 (C-9), 151.3 (d, ^1^*J*_CF_ = 247.3 Hz, C-7), 132.4 (C-3a), 126.2 (C-2), 126.2 (C-2 and C-7a, obscured by solvent), 120.3 (d, ^3^*J*_CF_ = 6.6 Hz, C-5), 115.5 (C-4), 110.5 (C-3), 107.3 (d, ^2^*J*_CF_ = 16.1 Hz, C-6), 48.3 (C-15, obscured by solvent), 46.0 (C-13), 36.7 (C-11), 33.8 (C-8), 29.9 (C-18), 27.7 (C-12), 27.4 (C-17), 27.2 (C-16); (+)-HRESIMS [M + H]^+^ *m/z* 609.3671 (calcd for C_34_H_47_F_2_N_6_O_2_, 609.3723).

#### 2.2.26. *N*^1^,*N*^10^-Bis(3-(2-(7-fluoro-1*H*-indol-3-yl)acetamido)propyl)decane-1,10-diaminium 2,2,2-trifluoroacetate (**18e**)

Following general procedure A, 7-fluoroindole-3-acetic acid (**10**) (0.040 g, 0.0207 mmol) was reacted with EDC**·**HCl (0.047 g, 0.245 mmol), HOBt (0.033 g, 0.245 mmol), DIPEA (0.10 mL, 0.565 mmol) and di-*tert*-butyl decane-1,10-diylbis((3-aminopropyl)carbamate) (**13e**) (0.046 g, 0.094 mmol) to afford di-*tert*-butyl decane-1,10-diylbis((3-(2-(7-fluoro-1*H*-indol-3-yl)acetamido)propyl)carbamate) (0.062 g, 79%) as a pale yellow oil. Following general procedure B, a sub-sample of this product (0.031 g, 0.031 mmol) was reacted with TFA in CH_2_Cl_2_ to yield, after chromatography, the di-TFA salt **18e** (0.016 g, 50%) as a yellow oil. R*_f_* (RP-18, 10% *aq* HCl:MeOH 1:3) 0.57; IR (ATR) ν_max_ 3266, 3082, 2829, 1669, 1645, 1581, 1436, 1365, 1199, 1180, 1127, 1048, 969, 835, 798, 721 cm^−1^; ^1^H NMR (CD_3_OD, 400 MHz) δ 7.37 (2H, d, *J* = 7.4 Hz, H-4), 7.26 (2H, s, H-2), 6.98 (2H, td, *J* = 7.9, 4.7 Hz, H-5), 6.85 (2H, dd, *J* = 11.5, 8.0 Hz, H-6), 3.69 (4H, s, H_2_-8), 3.30 (4H, obscured by solvent, H_2_-11), 2.80 (4H, t, *J* = 7.2 Hz, H_2_-13), 2.76 (4H, t, *J* = 7.8 Hz, H_2_-15), 1.80 (4H, tt, *J* = 6.8, 6.8 Hz, H_2_-12), 1.58–1.52 (4H, m, H_2_-16), 1.39–1.27 (12H, m, H_2_-17, H_2_-18 and H_2_-19); ^13^C NMR (CD_3_OD, 100 MHz) δ 176.0 (C-9), 151.2 (d, ^1^*J*_CF_ = 243.3 Hz, C-7), 132.5 (C-3a), 126.2 (C-2), 126.1 (C-2 and C-7a, obscured by solvent), 120.3 (d, ^3^*J*_CF_ = 6.0 Hz, C-5), 115.5 (d, ^4^*J*_CF_ = 3.4 Hz, C-4), 110.5 (C-3), 107.2 (d, ^2^*J*_CF_ = 16.5 Hz, C-6), 48.2 (C-15), 46.0 (C-13), 36.8 (C-11), 33.9 (C-8), 30.4 (C-19), 30.2 (C-18), 27.6 (C-12), 27.5 (C-17), 27.2 (C-16); (+)-HRESIMS [M + H]^+^ *m/z* 637.4040 (calcd for C_36_H_51_F_2_N_6_O_2_, 637.4036).

#### 2.2.27. *N*^1^,*N*^12^-Bis(3-(2-(7-fluoro-1*H*-indol-3-yl)acetamido)propyl)dodecane-1,12-diaminium 2,2,2-trifluoroacetate (**18f**)

Following general procedure A, 7-fluoroindole-3-acetic acid (**10**) (0.040 g, 0.0207 mmol) was reacted with EDC**·**HCl (0.047 g, 0.245 mmol), HOBt (0.033 g, 0.245 mmol), DIPEA (0.10 mL, 0.565 mmol) and di-*tert*-butyl dodecane-1,12-diylbis((3-aminopropyl)carbamate) (**13f**) (0.048 g, 0.094 mmol) to afford di-*tert*-butyl dodecane-1,12-diylbis((3-(2-(7-fluoro-1*H*-indol-3-yl)acetamido)propyl)carbamate) (0.025 g, 31%) as a pale yellow oil. Following general procedure B, a sub-sample of this product (0.009 g, 0.010 mmol) was reacted with TFA in CH_2_Cl_2_ to afford, after chromatography, the di-TFA salt **18f** (0.001 g, 11%) as a yellow oil. R*_f_* (RP-18, 10% *aq* HCl:MeOH 1:3) 0.50; IR (ATR) ν_max_ 2931, 2858, 1669, 1646, 1581, 1493, 1437, 1176, 1135, 1047, 969, 836, 798, 705, 721 cm^−1^; ^1^H NMR (CD_3_OD, 400 MHz) δ 7.37 (2H, d, *J* = 8.0 Hz, H-4), 7.25 (2H, s, H-2), 6.97 (2H, td, *J* = 7.8, 4.7 Hz, H-5), 6.84 (2H, dd, *J* = 11.5, 8.0 Hz, H-6), 3.68 (4H, s, H_2_-8), 3.29 (4H, t, *J* = 6.8 Hz, H_2_-11), 2.80 (4H, t, *J* = 7.2 Hz, H_2_-13), 2.74 (4H, t, *J* = 7.8 Hz, H_2_-15), 1.81 (4H, tt, *J* = 6.7, 6.6 Hz, H_2_-12), 1.57–1.51 (4H, m, H_2_-16), 1.34–1.28 (16H, m, H_2_-17, H_2_-18, H_2_-19 and H_2_-20); ^13^C NMR (CD_3_OD, 100 MHz) δ 176.0 (C-9), 151.2 (d, ^1^*J*_CF_ = 243.4 Hz, C-7), 132.4 (d, ^3^*J*_CF_ = 5.7 Hz, C-3a), 126.2 (C-2), 126.1 (C-2 and C-7a, obscured by solvent), 120.3 (d, ^3^*J*_CF_ = 6.1 Hz, C-5), 115.4 (d, ^4^*J*_CF_ = 3.2 Hz, C-4), 110.5 (d, ^4^*J*_CF_ = 2.3 Hz, C-3), 107.2 (d, ^2^*J*_CF_ = 16.5 Hz, C-6), 48.8 (C-15, obscured by solvent), 46.0 (C-13), 36.8 (C-11), 33.9 (C-8), 30.6 (C-20), 30.4 (C-19), 30.1 (C-18), 27.6 (C-12), 27.4 (C-17), 27.1 (C-16); (+)-HRESIMS [M + H]^+^ *m/z* 665.4344 (calcd for C_38_H_55_F_2_N_6_O_2_, 665.4349).

#### 2.2.28. *N*^1^,*N*^4^-Bis(3-(2-(7-methoxy-1*H*-indol-3-yl)acetamido)propyl)butane-1,4-diaminium 2,2,2-trifluoroacetate (**19a**)

Following general procedure A, 7-methoxyindole-3-acetic acid (**11**) (0.050 g, 0.244 mmol) was reacted with EDC**·**HCl (0.055 g, 0.288 mmol), HOBt (0.039 g, 0.288 mmol), DIPEA (0.12 mL, 0.665 mmol) and di-*tert*-butyl butane-1,4-diylbis((3-aminopropyl)carbamate) (**13a**) (0.045 g, 0.111 mmol) to afford di-*tert*-butyl butane-1,4-diylbis((3-(2-(7-methoxy-1*H*-indol-3-yl)acetamido)propyl)carbamate) (0.043 g, 50%) as a pale brown oil. Following general procedure B, a sub-sample of this product (0.011 g, 0.014 mmol) was reacted with TFA in CH_2_Cl_2_ to yield, after chromatography, the di-TFA salt **19a** (0.009 g, 79%) as a yellow oil. R*_f_* (RP-18, 10% *aq* HCl:MeOH 1:3) 0.65; IR (ATR) ν_max_ 2921, 1671, 1457, 1179, 1127, 834, 799, 721 cm^−1^; ^1^H NMR (CD_3_OD, 400 MHz) δ 7.16 (2H, d, *J* = 7.9 Hz, H-4), 7.15 (2H, s, H-2), 6.97 (2H, dd, *J* = 7.9, 7.7 Hz, H-5), 6.65 (2H, d, *J* = 7.7 Hz, H-6), 3.93 (6H, s, OMe), 3.68 (4H, s, H_2_-8), 3.31 (4H, obscured by solvent, H_2_-11), 2.78 (4H, t, *J* = 6.9 Hz, H_2_-13), 2.69 (4H, t, *J* = 7.3 Hz, H_2_-15), 1.80 (4H, tt, *J* = 6.6, 6.5 Hz, H_2_-12), 1.52 (4H, tt, *J* = 3.6, 3.6 Hz, H_2_-16); ^13^C NMR (CD_3_OD, 100 MHz) δ 176.7 (C-9), 148.0 (C-7), 129.9 (C-3a), 128.4 (C-7a), 124.8 (C-2), 120.8 (C-5), 112.2 (C-4), 109.8 (C-3), 102.8 (C-6), 55.8 (OMe), 47.8 (C-15), 45.8 (C-13), 36.5 (C-11), 34.0 (C-8), 27.7 (C-12), 23.9 (C-16); (+)-HRESIMS [M + H]^+^ *m/z* 577.3500 (calcd for C_32_H_45_N_6_O_4_, 577.3497).

#### 2.2.29. *N*^1^,*N*^6^-Bis(3-(2-(7-methoxy-1*H*-indol-3-yl)acetamido)propyl)hexane-1,6-diaminium 2,2,2-trifluoroacetate (**19b**)

Following general procedure A, 7-methoxyindole-3-acetic acid (**11**) (0.050 g, 0.264 mmol) was reacted with EDC**·**HCl (0.055 g, 0.288 mmol), HOBt (0.039 g, 0.288 mmol), DIPEA (0.12 mL, 0.665 mmol) and di-*tert*-butyl hexane-1,6-diylbis((3-aminopropyl)carbamate) (**13b**) (0.045 g, 0.111 mmol) to afford di-*tert*-butyl hexane-1,6-diylbis((3-(2-(7-methoxy-1*H*-indol-3-yl)acetamido)propyl)carbamate) (0.037 g, 41%) as a clear colorless oil. Following general procedure B, a sub-sample of the protected products (0.007 g, 0.009 mmol) was reacted with TFA in CH_2_Cl_2_ to afford, after chromatography, the di-TFA salt **19b** (0.005 g, 69%) as a brown oil. R*_f_* (RP-18, 10% *aq* HCl:MeOH 1:3) 0.60; IR (ATR) ν_max_ 2922, 1671, 1457, 1178, 1127, 834, 799, 721 cm^−1^; ^1^H NMR (CD_3_OD, 400 MHz) δ 7.16 (2H, d, *J* = 7.9 Hz, H-4), 7.15 (2H, s, H-2), 6.97 (2H, dd, *J* = 7.9, 7.6 Hz, H-5), 6.65 (2H, d, *J* = 7.6 Hz, H-6), 3.93 (6H, s, OMe), 3.67 (4H, s, H_2_-8), 3.30 (4H, obscured by solvent, H_2_-11), 2.78 (4H, t, *J* = 7.0 Hz, H_2_-13), 2.72 (4H, t, *J* = 7.3 Hz, H_2_-15), 1.80 (4H, tt, *J* = 6.7, 6.6 Hz, H_2_-12), 1.52 (4H, tt, *J* = 7.5, 7.4 Hz, H_2_-16), 1.29–1.27 (4H, m, H_2_-17); ^13^C NMR (CD_3_OD, 100 MHz) δ 176.6 (C-9), 148.0 (C-7), 129.9 (C-3a), 128.4 (C-7a), 124.7 (C-2), 120.7 (C-5), 112.2 (C-4), 109.8 (C-3), 102.8 (C-6), 55.8 (OMe), 48.5 (C-15, obscured by solvent), 45.9 (C-13), 36.6 (C-11), 34.1 (C-8), 27.6 (C-12), 26.83 (C-17), 26.78 (C-16); (+)-HRESIMS [M + H]^+^ *m/z* 605.3810 (calcd for C_34_H_43_N_6_O_4_, 605.3810).

#### 2.2.30. *N*^1^,*N*^7^-Bis(3-(2-(7-methoxy-1*H*-indol-3-yl)acetamido)propyl)heptane-1,7-diaminium 2,2,2-trifluoroacetate (**19c**)

Following general procedure A, 7-methoxyindole-3-acetic acid (**11**) (0.050 g, 0.244 mmol) was reacted with EDC**·**HCl (0.055 g, 0.288 mmol), HOBt (0.039 g, 0.288 mmol), DIPEA (0.12 mL, 0.665 mmol) and di-*tert*-butyl heptane-1,7-diylbis((3-aminopropyl)carbamate) (**13c**) (0.049 g, 0.111 mmol) to afford di-*tert*-butyl heptane-1,7-diylbis((3-(2-(7-methoxy-1*H*-indol-3-yl)acetamido)propyl)carbamate) (0.040 g, 44%) as a clear colorless oil. Following general procedure B, a sub-sample of this product (0.021 g, 0.026 mmol) was reacted with TFA in CH_2_Cl_2_ to afford, after chromatography, the di-TFA salt **19c** (0.008 g, 37%) as a yellow oil. R*_f_* (RP-18, 10% *aq* HCl:MeOH 1:3) 0.57; IR (ATR) ν_max_ 2922, 1671, 1457, 1179, 1127, 834, 799, 720 cm^−1^; ^1^H NMR (CD_3_OD, 400 MHz) δ 7.16 (2H, d, *J* = 7.6 Hz, H-4), 7.15 (2H, s, H-2), 6.96 (2H, dd, *J* = 7.7, 7.6 Hz, H-5), 6.65 (2H, d, *J* = 7.7 Hz, H-6), 3.93 (6H, s, OMe), 3.66 (4H, s, H_2_-8), 3.29 (4H, t, *J* = 6.5 Hz, H_2_-11), 2.77 (4H, t, *J* = 7.1 Hz, H_2_-13), 2.72 (4H, t, *J* = 7.8 Hz, H_2_-15), 1.80 (4H, tt, *J* = 6.6, 6.4 Hz, H_2_-12), 1.53 (4H, tt, *J* = 7.6, 7.5 Hz, H_2_-16), 1.33–1.28 (6H, m, H_2_-17 and H_2_-18); ^13^C NMR (CD_3_OD, 100 MHz) δ 176.5 (C-9), 148.0 (C-7), 129.8 (C-3a), 128.4 (C-7a), 124.7 (C-2), 120.7 (C-5), 112.2 (C-4), 109.8 (C-3), 102.7 (C-6), 55.8 (OMe), 48.4 (C-15, obscured by solvent), 45.9 (C-13), 36.6 (C-11), 34.1 (C-8), 29.5 (C-18), 27.6 (C-12), 27.1 (C-17), 27.0 (C-16); (+)-HRESIMS [M + H]^+^ *m/z* 619.3946 (calcd for C_35_H_51_N_6_O_4_, 619.3966).

#### 2.2.31. *N*^1^,*N*^8^-Bis(3-(2-(7-methoxy-1*H*-indol-3-yl)acetamido)propyl)octane-1,8-diaminium 2,2,2-trifluoroacetate (**19d**)

Following general procedure A, 7-methoxyindole-3-acetic acid (**11**) (0.050 g, 0.264 mmol) was reacted with EDC**·**HCl (0.055 g, 0.288 mmol), HOBt (0.039 g, 0.288 mmol), DIPEA (0.12 mL, 0.665 mmol) and di-*tert*-butyl octane-1,8-diylbis((3-aminopropyl)carbamate) (**13d**) (0.051 g, 0.111 mmol) to afford di-*tert*-butyl octane-1,8-diylbis((3-(2-(7-methoxy-1*H*-indol-3-yl)acetamido)propyl)carbamate) (0.043 g, 47%) as a pale yellow oil. Following general procedure B, a sub-sample of this product (0.021 g, 0.025 mmol) was reacted with TFA in CH_2_Cl_2_ to afford, after chromatography, the di-TFA salt **19d** (0.011 g, 51%) as a yellow-brown oil. R*_f_* (RP-18, 10% *aq* HCl:MeOH 1:3) 0.55; IR (ATR) ν_max_ 2922, 1671, 1457, 1178, 1128, 835, 799, 720 cm^−1^; ^1^H NMR (CD_3_OD, 400 MHz) δ 7.17 (2H, d, *J* = 7.8 Hz, H-4), 7.16 (2H, d, *J* = 1.2 Hz, H-2), 6.96 (2H, dd, *J* = 7.8, 7.7 Hz, H-5), 6.65 (2H, d, *J* = 7.7 Hz, H-6), 3.94 (6H, s, OMe), 3.67 (4H, s, H_2_-8), 3.29 (4H, obscured by solvent, H_2_-11), 2.77 (4H, t, *J* = 7.1 Hz, H_2_-13), 2.72 (4H, t, *J* = 7.8 Hz, H_2_-15), 1.79 (4H, tt, *J* = 6.7, 6.6 Hz, H_2_-12), 1.58–1.51 (4H, m, H_2_-16), 1.35–1.29 (8H, m, H_2_-17 and H_2_-18); ^13^C NMR (CD_3_OD, 100 MHz) δ 176.6 (C-9), 148.1 (C-7), 129.8 (C-3a), 128.5 (C-7a), 124.7 (C-2), 120.7 (C-5), 112.2 (C-4), 109.8 (C-3), 102.8 (C-6), 55.8 (OMe), 48.3 (C-15, obscured by solvent), 45.9 (C-13), 36.6 (C-11), 34.1 (C-8), 29.9 (C-18), 27.7 (C-12), 27.3 (C-17), 27.2 (C-16); (+)-HRESIMS [M + H]^+^ *m/z* 633.4111 (calcd for C_36_H_53_N_6_O_4_, 633.4123).

#### 2.2.32. *N*^1^,*N*^10^-Bis(3-(2-(7-methoxy-1*H*-indol-3-yl)acetamido)propyl)decane-1,10-diaminium 2,2,2-trifluoroacetate (**19e**)

Following general procedure A, 7-methoxyindole-3-acetic acid (**11**) (0.050 g, 0.244 mmol) was reacted with EDC**·**HCl (0.055 g, 0.288 mmol), HOBt (0.039 g, 0.288 mmol), DIPEA (0.12 mL, 0.665 mmol) and di-*tert*-butyl decane-1,10-diylbis((3-aminopropyl)carbamate) (**13e**) (0.054 g, 0.111 mmol) to yield di-*tert*-butyl decane-1,10-diylbis((3-(2-(7-methoxy-1*H*-indol-3-yl)acetamido)propyl)carbamate) (0.031 g, 33%) as a clear brown oil. Following general procedure B, a sub-sample of this product (0.015 g, 0.017 mmol) was reacted with TFA in CH_2_Cl_2_ to afford, after chromatography, the di-TFA salt **19e** (0.009 g, 58%) as a yellow oil. R*_f_* (RP-18, 10% *aq* HCl:MeOH 1:3) 0.50; IR (ATR) ν_max_ 3366, 3290, 3053, 2932, 2857, 1668, 1579, 1502, 1433, 1376, 1261, 1202, 1178, 1128, 1092, 1052, 941, 840, 797, 773, 721 cm^−1^; ^1^H NMR (CD_3_OD, 400 MHz) δ 7.17 (2H, d, *J* = 7.9 Hz, H-4), 7.16 (2H, d, *J* = 0.8 Hz, H-2), 6.96 (2H, dd, *J* = 7.9, 7.5 Hz, H-5), 6.65 (2H, d, *J* = 7.5 Hz, H-6), 3.94 (6H, s, OMe), 3.66 (4H, s, H_2_-8), 3.29 (4H, obscured by solvent, H_2_-11), 2.76 (4H, t, *J* = 7.2 Hz, H_2_-13), 2.71 (4H, t, *J* = 7.8 Hz, H_2_-15), 1.78 (4H, tt, *J* = 6.8, 6.7 Hz, H_2_-12), 1.54 (4H, tt, *J* = 7.5, 7.5 Hz, H_2_-16), 1.36–1.29 (12H, m, H_2_-17, H_2_-18 and H_2_-19); ^13^C NMR (CD_3_OD, 100 MHz) δ 176.5 (C-9), 148.0 (C-7), 129.8 (C-3a), 128.5 (C-7a), 124.7 (C-2), 120.7 (C-5), 112.2 (C-4), 109.8 (C-3), 102.7 (C-6), 55.8 (OMe), 48.9 (C-15, obscured by solvent), 45.9 (C-13), 36.7 (C-11), 34.1 (C-8), 30.4 (C-19), 30.2 (C-18), 27.6 (C-12), 27.5 (C-17), 27.2 (C-16); (+)-HRESIMS [M + H]^+^ *m/z* 661.4433 (calcd for C_38_H_57_N_6_O_4_, 661.4436).

#### 2.2.33. *N*^1^,*N*^12^-Bis(3-(2-(7-methoxy-1*H*-indol-3-yl)acetamido)propyl)dodecane-1,12-diaminium 2,2,2-trifluoroacetate (**19f**)

Following general procedure A, 7-methoxyindole-3-acetic acid (**11**) (0.050 g, 0.244 mmol) was reacted with EDC**·**HCl (0.055 g, 0.288 mmol), HOBt (0.039 g, 0.288 mmol), DIPEA (0.12 mL, 0.665 mmol) and di-*tert*-butyl dodecane-1,12-diylbis((3-aminopropyl)carbamate) (**13f**) (0.057 g, 0.111 mmol) to afford di-*tert*-butyl dodecane-1,12-diylbis((3-(2-(7-methoxy-1*H*-indol-3-yl)acetamido)propyl)carbamate) (0.029 g, 29%) as a pale yellow oil. Following general procedure B, a sub-sample of this product (0.007 g, 0.008 mmol) was reacted with TFA in CH_2_Cl_2_ to afford, after chromatography, the di-TFA salt **19f** (0.001 g, 14%) as a yellow oil. R*_f_* (RP-18, 10% *aq* HCl:MeOH 1:3) 0.47; IR (ATR) ν_max_ 3367, 3290, 3051, 2932, 2857, 1668, 1579, 1502, 1434, 1375, 1261, 1202, 1178, 1128, 1092, 1052, 941, 840, 797, 774, 721 cm^−1^; ^1^H NMR (CD_3_OD, 400 MHz) δ 7.17 (2H, d, *J* = 7.8 Hz, H-4), 7.16 (2H, d, *J* = 0.8 Hz, H-2), 6.96 (2H, dd, *J* = 7.8, 7.6 Hz, H-5), 6.65 (2H, d, *J* = 7.6 Hz, H-6), 3.94 (6H, s, OMe), 3.66 (4H, s, H_2_-8), 3.29 (4H, obscured by solvent, H_2_-11), 2.76 (4H, t, *J* = 7.1 Hz, H_2_-13), 2.70 (4H, t, *J* = 7.9 Hz, H_2_-15), 1.77 (4H, tt, *J* = 6.8, 6.7 Hz, H_2_-12), 1.56–1.50 (4H, m, H_2_-16), 1.38–1.28 (16H, m, H_2_-17, H_2_-18, H_2_-19 and H_2_-20); ^13^C NMR (CD_3_OD, 100 MHz) δ 176.5 (C-9), 148.0 (C-7), 129.8 (C-3a), 128.5 (C-7a), 124.7 (C-2), 120.7 (C-5), 112.2 (C-4), 109.8 (C-3), 102.7 (C-6), 55.8 (OMe), 48.8 (C-15, obscured by solvent), 45.9 (C-13), 36.6 (C-11), 34.1 (C-8), 30.7 (C-20), 30.6 (C-19), 30.2 (C-18), 27.6 (C-12), 27.5 (C-17), 27.2 (C-16); (+)-HRESIMS [M + H]^+^ *m/z* 689.4747 (calcd for C_40_H_61_N_6_O_4_, 689.4749).

#### 2.2.34. *N*^1^,*N*^4^-Bis(3-(2-(7-methyl-1*H*-indol-3-yl)acetamido)propyl)butane-1,4-diaminium 2,2,2-trifluoroacetate (**20a**)

Following general procedure A, 7-methylindole-3-acetic acid (**12**) (0.050 g, 0.264 mmol) was reacted with EDC**·**HCl (0.060 g, 0.312 mmol), HOBt (0.042 g, 0.312 mmol), DIPEA (0.13 mL, 0.721 mmol) and di-*tert*-butyl butane-1,4-diylbis((3-aminopropyl)carbamate) (**13a**) (0.048 g, 0.120 mmol) to afford di-*tert*-butyl butane-1,4-diylbis((3-(2-(7-methyl-1*H*-indol-3-yl)acetamido)propyl)carbamate) (0.015 g, 17%) as a clear colorless oil. Following general procedure B, a sub-sample of this product (0.013 g, 0.018 mmol) was reacted with TFA in CH_2_Cl_2_ to afford, after chromatography, the di-TFA salt **20a** (0.012 g, 89%) as a purple oil. R*_f_* (RP-18, 10% *aq* HCl:MeOH 1:3) 0.55; IR (ATR) ν_max_ 3288, 2834, 1669, 1542, 1435, 1340, 1199, 1183, 1130, 836, 800, 747, 721 cm^−1^; ^1^H NMR (CD_3_OD, 400 MHz) δ 7.39 (2H, dd, *J* = 7.4, 0.9 Hz, H-4), 7.20 (2H, s, H-2), 6.95 (2H, t, *J* = 7.3 Hz, H-5), 6.92 (2H, d, *J* = 6.4 Hz, H-6), 3.69 (4H, s, H_2_-8), 3.30 (4H, obscured by solvent, H_2_-11), 2.78 (4H, t, *J* = 6.9 Hz, H_2_-13), 2.72 (4H, t, *J* = 7.8 Hz, H_2_-15), 2.47 (6H, s, Me), 1.80 (4H, tt, *J* = 6.6, 6.5 Hz, H_2_-12), 1.56 (4H, tt, *J* = 3.6, 3.6 Hz, H_2_-16); ^13^C NMR (CD_3_OD, 100 MHz) δ 176.7 (C-9), 137.6 (C-7a), 128.1 (C-3a), 125.1 (C-2), 123.2 (C-6), 122.2 (C-7), 120.4 (C-5), 117.0 (C-4), 109.7 (C-3), 47.9 (C15), 45.9 (C-13), 36.6 (C-11), 34.1 (C-8), 27.7 (C-12), 24.0 (C-16), 16.9 (Me); (+)-HRESIMS [M + Na]^+^ *m*/*z* 567.3417 (calcd for C_32_H_44_N_6_NaO_2_, 567.3418).

#### 2.2.35. *N*^1^,*N*^6^-Bis(3-(2-(7-methyl-1*H*-indol-3-yl)acetamido)propyl)hexane-1,6-diaminium 2,2,2-trifluoroacetate (**20b**)

Following general procedure A, 7-methylindole-3-acetic acid (**12**) (0.050 g, 0.264 mmol) was reacted with EDC**·**HCl (0.060 g, 0.312 mmol), HOBt (0.042 g, 0.312 mmol), DIPEA (0.13 mL, 0.721 mmol) and di-*tert*-butyl hexane-1,6-diylbis((3-aminopropyl)carbamate) (**13b**) (0.052 g, 0.120 mmol) to yield di-*tert*-butyl hexane-1,6-diylbis((3-(2-(7-methyl-1*H*-indol-3-yl)acetamido)propyl)carbamate) (0.058 g, 62%) as a pale yellow oil. Following general procedure B, a sub-sample of this product (0.029 g, 0.038 mmol) was reacted with TFA in CH_2_Cl_2_ to afford, after chromatography, the di-TFA salt **20b** (0.016 g, 53%) as a yellow oil. R*_f_* (RP-18, 10% *aq* HCl:MeOH 1:3) 0.48; IR (ATR) ν_max_ 3289, 3065, 2833, 1669, 1542, 1436, 1340, 1199, 1181, 1129, 835, 799, 748, 721 cm^−1^; ^1^H NMR (CD_3_OD, 400 MHz) δ 7.40 (2H, d, *J* = 7.3 Hz, H-4), 7.20 (2H, s, H-2), 6.97–6.91 (4H, m, H-5 and H-6), 3.68 (4H, s, H_2_-8), 3.29 (4H, obscured by solvent, H_2_-11), 2.77 (4H, t, *J* = 7.0 Hz, H_2_-13), 2.71 (4H, t, *J* = 7.6 Hz, H_2_-15), 2.47 (6H, s, Me), 1.79 (4H, tt, *J* = 6.5, 6.3 Hz, H_2_-12), 1.57–1.50 (4H, m, H_2_-16), 1.31–1.26 (4H, m, H_2_-17); ^13^C NMR (CD_3_OD, 100 MHz) δ 176.5 (C-9), 137.6 (C-7a), 128.0 (C-3a), 125.0 (C-2), 123.2 (C-6), 122.1 (C-7), 120.3 (C-5), 117.0 (C-4), 109.7 (C-3), 48.5 (C-15, obscured by solvent), 45.9 (C-13), 36.7 (C-11), 34.1 (C-8), 27.6 (C-12), 26.82 (C-17), 26.77 (C-16), 16.9 (Me); (+)-HRESIMS [M + H]^+^ *m/z* 573.3901 (calcd for C_34_H_49_N_6_O_2_, 573.3912).

#### 2.2.36. *N*^1^,*N*^7^-Bis(3-(2-(7-methyl-1*H*-indol-3-yl)acetamido)propyl)heptane-1,7-diaminium 2,2,2-trifluoroacetate (**20c**)

Following general procedure A, 7-methylindole-3-acetic acid (**12**) (0.050 g, 0.264 mmol) was reacted with EDC**·**HCl (0.060 g, 0.312 mmol), HOBt (0.042 g, 0.312 mmol), DIPEA (0.13 mL, 0.721 mmol) and di-*tert*-butyl heptane-1,7-diylbis((3-aminopropyl)carbamate) (**13c**) (0.053 g, 0.120 mmol) to afford di-*tert*-butyl heptane-1,7-diylbis((3-(2-(7-methyl-1*H*-indol-3-yl)acetamido)propyl)carbamate) (0.027 g, 29%) as a clear colorless oil. Following general procedure B, this product (0.027 g, 0.034 mmol) was reacted with TFA in CH_2_Cl_2_ to afford, after chromatography, the di-TFA salt **20c** (0.021 g, 75%) as a clear colorless oil. R*_f_* (RP-18, 10% *aq* HCl:MeOH 1:3) 0.40; IR (ATR) ν_max_ 3279, 2939, 2859, 1671, 1554, 1436, 1344, 1199, 1178, 1128, 834, 799, 747, 720 cm^−1^; ^1^H NMR (CD_3_OD, 400 MHz) δ 7.40 (2H, dd, *J* = 7.4, 1.1 Hz, H-4), 7.21 (2H, s, H-2), 6.95 (2H, t, *J* = 7.3 Hz, H-5), 6.92 (2H, d, *J* = 6.5 Hz, H-6), 3.68 (4H, s, H_2_-8), 3.28 (4H, t, *J* = 6.4 Hz, H_2_-11), 2.76 (4H, t, *J* = 7.1 Hz, H_2_-13), 2.70 (4H, t, *J* = 7.7 Hz, H_2_-15), 2.48 (6H, s, Me), 1.78 (4H, tt, *J* = 6.9, 6.8 Hz, H_2_-12), 1.54 (4H, tt, *J* = 7.6, 7.4 Hz, H_2_-16), 1.33–1.27 (6H, m, H_2_-17 and H_2_-18); ^13^C NMR (CD_3_OD, 100 MHz) δ 176.4 (C-9), 137.6 (C-7a), 128.0 (C-3a), 125.0 (C-2), 123.2 (C-6), 122.1 (C-7), 120.3 (C-5), 117.0 (C-4), 109.8 (C-3), 49.8 (C15), 45.9 (C-13), 36.7 (C-11), 34.1 (C-8), 29.5 (C-18), 27.6 (C-12), 27.1 (C-17), 27.0 (C-16), 16.9 (Me); (+)-HRESIMS [M + H]^+^ *m/z* 587.4065 (calcd for C_35_H_51_N_6_O_2_, 587.4068).

#### 2.2.37. *N*^1^,*N*^8^-Bis(3-(2-(7-methyl-1*H*-indol-3-yl)acetamido)propyl)octane-1,8-diaminium 2,2,2-trifluoroacetate (**20d**)

Following general procedure A, 7-methylindole-3-acetic acid (**12**) (0.050 g, 0.264 mmol) was reacted with EDC**·**HCl (0.060 g, 0.312 mmol), HOBt (0.042 g, 0.312 mmol), DIPEA (0.13 mL, 0.721 mmol) and di-*tert*-butyl octane-1,8-diylbis((3-aminopropyl)carbamate) (**13d**) (0.55 g, 0.120 mmol) to yield di-*tert*-butyl octane-1,8-diylbis((3-(2-(7-methyl-1*H*-indol-3-yl)acetamido)propyl)carbamate) (0.052, 54%) as a clear colorless oil. Following general procedure B, a sub-sample of this product (0.020 g, 0.025 mmol) was reacted with TFA in CH_2_Cl_2_ to afford, after chromatography, the di-TFA salt **20d** (0.014 g, 68%) as a pale yellow oil. R*_f_* (RP-18, 10% *aq* HCl:MeOH 1:3) 0.38; IR (ATR) ν_max_ 3280, 3057, 2940, 2860, 1670, 1555, 1436, 1344, 1199, 1178, 1128, 834, 799, 747, 720 cm^−1^; ^1^H NMR (CD_3_OD, 400 MHz) δ 7.40 (2H, d, *J* = 7.3 Hz, H-4), 7.21 (2H, s, H-2), 6.97–6.91 (4H, m, H-5 and H-6), 3.68 (4H, s, H_2_-8), 3.28 (4H, obscured by solvent, H_2_-11), 2.76 (4H, t, *J* = 7.0 Hz, H_2_-13), 2.71 (4H, t, *J* = 7.8 Hz, H_2_-15), 2.48 (6H, s, Me), 1.78 (4H, tt, *J* = 6.8, 6.7 Hz, H_2_-12), 1.54 (4H, tt, *J* = 7.4, 7.2 Hz, H_2_-16), 1.35–1.28 (8H, m, H_2_-17 and H_2_-18); ^13^C NMR (CD_3_OD, 100 MHz) δ 176.5 (C-9), 137.6 (C-7a), 128.0 (C-3a), 125.0 (C-2), 123.2 (C-6), 122.1 (C-7), 120.3 (C-5), 117.0 (C-4), 109.7 (C-3), 48.4 (C-15, obscured by solvent), 45.9 (C-13), 36.6 (C-11), 34.2 (C-8), 29.9 (C-18), 27.6 (C-12), 27.3 (C-17), 27.1 (C-16), 16.9 (Me); (+)-HRESIMS [M + H]^+^ *m/z* 601.4224 (calcd for C_36_H_53_N_6_O_2_, 601.4225).

#### 2.2.38. *N*^1^,*N*^10^-Bis(3-(2-(7-methyl-1*H*-indol-3-yl)acetamido)propyl)decane-1,10-diaminium 2,2,2-trifluoroacetate (**20e**)

Following general procedure A, 7-methylindole-3-acetic acid (**12**) (0.025 g, 0.132 mmol) was reacted with EDC**·**HCl (0.030 g, 0.156 mmol), HOBt (0.021 g, 0.156 mmol), DIPEA (0.07 mL, 0.311 mmol) and di-*tert*-butyl decane-1,10-diylbis((3-aminopropyl)carbamate) (**13e**) (0.029 g, 0.060 mmol) to yield di-*tert*-butyl decane-1,10-diylbis((3-(2-(7-methyl-1*H*-indol-3-yl)acetamido)propyl)carbamate) (0.037 g, 74%) as a clear colorless oil. Following general procedure B, a sub-sample of this product (0.014 g, 0.017 mmol) was reacted with TFA in CH_2_Cl_2_ to afford, after chromatography, the di-TFA salt **20e** (0.010 g, 69%) as a pale brown oil. R*_f_* (RP-18, 10% *aq* HCl:MeOH 1:3) 0.37; IR (ATR) ν_max_ 3280, 3053, 2940, 1670, 1542, 1436, 1344, 1199, 1179, 1128, 834, 799, 747, 720 cm^−1^; ^1^H NMR (CD_3_OD, 400 MHz) δ 7.41 (2H, dd, *J* = 7.5, 1.1 Hz, H-4), 7.21 (2H, s, H-2), 6.97–6.91 (4H, m, H-5 and H-6), 3.68 (4H, s, H_2_-8), 3.29 (4H, obscured by solvent, H_2_-11), 2.76 (4H, t, *J* = 7.2 Hz, H_2_-13), 2.70 (4H, t, *J* = 7.9 Hz, H_2_-15), 2.48 (6H, s, Me), 1.78 (4H, tt, *J* = 6.8, 6.7 Hz, H_2_-12), 1.54 (4H, tt, *J* = 7.1, 7.1 Hz, H_2_-16), 1.35–1.29 (12H, m, H_2_-17, H_2_-18 and H_2_-19); ^13^C NMR (CD_3_OD, 100 MHz) δ 176.5 (C-9), 137.6 (C-7a), 128.0 (C-3a), 125.0 (C-2), 123.2 (C-6), 122.1 (C-7), 120.3 (C-5), 117.0 (C-4), 109.8 (C-3), 48.9 (C-15), 45.9 (C-13), 36.7 (C-11), 34.2 (C-8), 30.4 (C-19), 30.2 (C-18), 27.6 (C-12), 27.5 (C-17), 27.2 (C-16), 16.9 (Me); (+)-HRESIMS [M + H]^+^ *m/z* 629.4531 (calcd for C_38_H_57_N_6_O_2_, 629.4538).

#### 2.2.39. *N*^1^,*N*^12^-Bis(3-(2-(7-methyl-1*H*-indol-3-yl)acetamido)propyl)dodecane-1,12-diaminium 2,2,2-trifluoroacetate (**20f**)

Following general procedure A, 7-methylindole-3-acetic acid (**12**) (0.025 g, 0.132 mmol) was reacted with EDC**·**HCl (0.030 g, 0.156 mmol), HOBt (0.021 g, 0.156 mmol), DIPEA (0.07 mL, 0.311 mmol) and di-*tert*-butyl dodecane-1,12-diylbis((3-aminopropyl)carbamate) (**13f**) (0.031 g, 0.060 mmol) to afford di-*tert*-butyl dodecane-1,12-diylbis((3-(2-(7-methyl-1*H*-indol-3-yl)acetamido)propyl)carbamate) (0.035 g, 68%) as a clear oil. Following general procedure B, a sub-sample of this product (0.025 g, 0.029 mmol) was reacted with TFA in CH_2_Cl_2_ to afford, after chromatography, the di-TFA salt **20f** (0.011 g, 43%) as a brown oil. R*_f_* (RP-18, 10% *aq* HCl:MeOH 1:3) 0.33; IR (ATR) ν_max_ 3275, 2938, 2860, 1670, 1542, 1436, 1344, 1199, 1179, 1129, 834, 799, 747, 721 cm^−1^; ^1^H NMR (CD_3_OD, 400 MHz) δ 7.41 (2H, dd, *J* = 7.1, 1.2 Hz, H-4), 7.21 (2H, s, H-2), 6.95 (2H, t, *J* = 7.3 Hz, H-5), 6.92 (2H, d, *J* = 6.4 Hz, H-6), 3.68 (4H, s, H_2_-8), 3.29 (4H, obscured by solvent, H_2_-11), 2.75 (4H, t, *J* = 7.2 Hz, H_2_-13), 2.69 (4H, t, *J* = 7.8 Hz, H_2_-15), 2.48 (6H, s, Me), 1.77 (4H, tt, *J* = 6.8, 6.7 Hz, H_2_-12), 1.52 (4H, tt, *J* = 7.8, 7.0 Hz, H_2_-16), 1.38–1.28 (16H, m, H_2_-17, H_2_-18, H_2_-19 and H_2_-20); ^13^C NMR (CD_3_OD, 100 MHz) δ 176.5 (C-9), 137.7 (C-7a), 128.0 (C-3a), 125.1 (C-2), 123.2 (C-6), 122.1 (C-7), 120.3 (C-5), 117.0 (C-4), 109.8 (C-3), 48.5 (C-15, obscured by solvent), 45.9 (C-13), 36.6 (C-11), 34.2 (C-8), 30.7 (C-20), 30.6 (C-19), 30.2 (C-18), 27.6 (C-12), 27.5 (C-17), 27.2 (C-16), 16.9 (Me); (+)-HRESIMS [M + H]^+^ *m/z* 657.4846 (calcd for C_40_H_61_N_6_O_2_, 657.4851).

### 2.3. Antimicrobial Assays

The susceptibility of bacterial strains *Staphylococcus aureus* (ATCC 25923), *Escherichia coli* (ATCC 25922) and *Pseudomonas aeruginosa* (ATCC 27853) to antibiotics and compounds was determined using previously reported protocols [16]. Additional antimicrobial evaluation against MRSA (ATCC 43300), *Klebsiella pneumoniae* (ATCC 700603), *Acinetobacter baumannii* (ATCC 19606), *Candida albicans* (ATCC 90028) and *Cryptococcus neoformans* (ATCC 208821) was undertaken at the Community for Open Antimicrobial Drug Discovery at The University of Queensland (Australia) according to their standard protocols, as reported previously [21].

### 2.4. Determination of the MICs of Antibiotics in the Presence of Synergizing Compounds

Antibiotic enhancer concentrations were determined using previously reported protocols [16].

### 2.5. Cytotoxicity Assays

Cytotoxicity assays were conducted using the protocols previously reported [16,21].

### 2.6. Hemolytic Assay

Hemolysis assays were conducted using the protocols previously reported [16,21].

## 3. Results and Discussion

**Figure 3:**
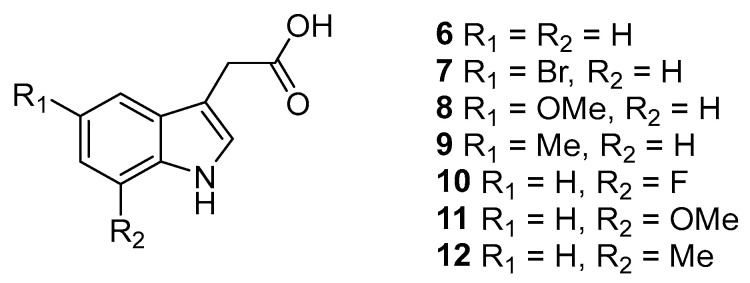
The structures of indole-3-acetic acid head groups **6**–**12**.

The expanded set of indole-3-acetamido-polyamines was comprised of seven capping acids, namely indole-3-acetic acid (**6**) and the 5-bromo- (**7**), 5-methoxyl- (**8**), 5-methyl- (**9**), 7-fluoro- (**10**), 7-methoxyl- (**11**) and 7-methyl- (**12**) analogues (Figure 3). These particular substituents and the substituent position on the indole ring were selected as they had previously been shown to improve intrinsic antimicrobial and antibiotic enhancement properties [12,16]. All seven were available from commercial sources.

**Figure 4:**
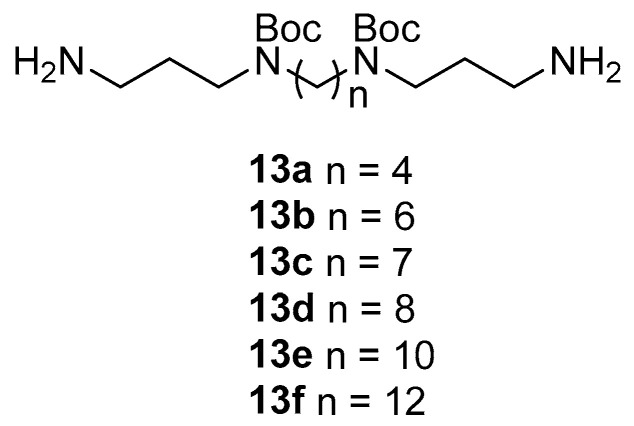
Polyamine scaffolds **13a**–**f**.

The second component of the target conjugates was the polyamine core. In order to explore any variation of biological activities associated with this core, a set of six Boc-protected polyamines (**13a**–**f**) (Figure 4) were synthesized according to the methods described previously [17,18,19,20].

**Scheme 1:**
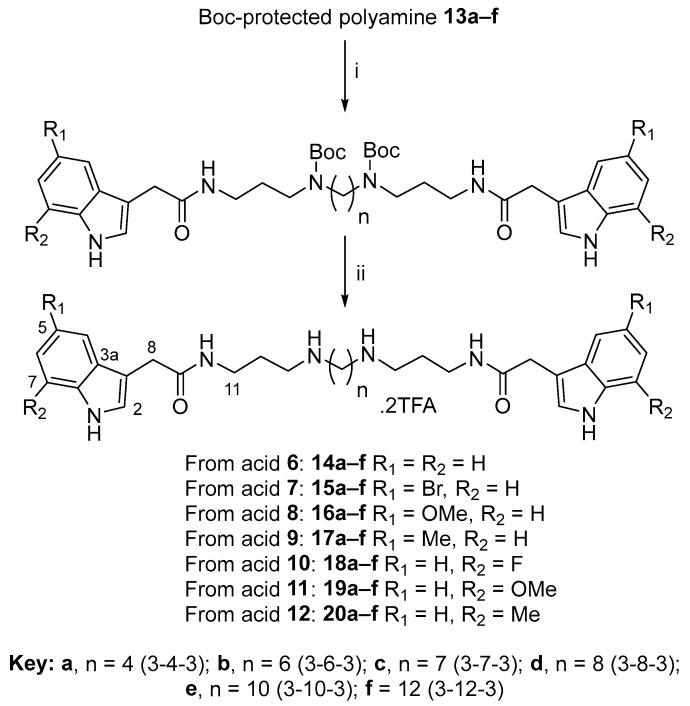
General method for the synthesis of target polyamine analogues **14–20**. Reagents and conditions: (i) Carboxylic acid RCO_2_H (**6**–**12**) (2.2 equiv.), Boc-protected polyamine (**13a**–**f**) (1.0 equiv.), EDC·HCl (2.6 equiv.), HOBt (2.6 equiv.), DIPEA (6 equiv.), in either CH_2_Cl_2_ or DMF, 0 °C, N_2_, 24 h (yields 17–85%); (ii) TFA (0.2 mL), CH_2_Cl_2_ (2 mL), r.t., 2 h (yields 11–97%).

The target conjugates were then synthesized in a two-step sequence. In the first step, amide bond formation between the indole-3-acetic acids (**6**–**12**) and Boc-protected polyamines **13a**–**f** was performed using the reagent combination of EDC**·**HCl and HOBt in either CH_2_Cl_2_ or DMF solvent. These Boc-protected intermediates were then deprotected using TFA/CH_2_Cl_2_ to give the target polyamine conjugates **14**–**20** as their di-TFA salts (Scheme 1) (Figure S1–S39).

Initially, the library of indole-3-acetamido-polyamine conjugates was screened for intrinsic antimicrobial activity against *S. aureus* and methicillin-resistant *S. aureus* (MRSA), *P. aeruginosa*, *E. coli*, *K. pneumoniae*, *A. baumannii, C. albicans* and *C. neoformans*, with the results recorded as minimum inhibitory concentrations (MIC) (Table 1). In general, the compounds tested showed excellent activity towards MRSA and *C. neoformans*, with exceptions being the previously reported spermine analogues **14a**, **15a** and **16a**, 5-methoxy analogues **16b** and **16c** (inactive towards MRSA), and 7-methoxy analogues **19a** 3-4-3, **19c** 3-7-3 and **19d** 3-8-3 (also inactive against MRSA). Activity towards the fungus *C. albicans* was mainly associated with 5-bromo analogues (e.g., **15c**, **15e**, **15f**) and longer polyamine chain analogues bearing an unsubstituted indole-3-acetamide capping group (**14e**, **14f**), 5-methyl (e.g., **17e**, **17f**), 7-fluoro (e.g., **17f**) and 7-methyl (e.g., **20f**) substituents. None of the compounds in the set exhibited activity towards the Gram-negative bacteria *P. aeruginosa*, *K. pneumoniae* and *A. baumannii*, though a limited subset of the analogues exhibited activity towards *E. coli*, with the only notable example being the 5-methoxyindole analogue **16d** with an MIC value of 6.25 µM.

The compound set was then evaluated for cytotoxicity, reported as the concentration of compound at 50% cytotoxicity (IC_50_) towards the HEK293 cell line, and for hemolytic properties, reported as the concentration of compound at 10% hemolytic activity (HC_10_) against human red blood cells (Table 2). While cytotoxicity was observed for just two analogues (**15f**, IC_50_ 4.75 µM; **18f**, IC_50_ 27.2 µM), hemolytic properties were more widespread, with twenty analogues identified with HC_10_ values less than 30 µM. Overall, cytotoxicity and hemolytic properties tended to be associated with longer polyamine chain variants, with obvious exceptions being the 5-methyl and 7-methyl substituted examples, which also demonstrated hemolytic properties for the shorter PA-3-4-3 (spermine) (e.g., **17a** and **20a**) and PA-3-6-3 (e.g., **17b** and **20b**) analogues. Notably, none of the 5-methoxy analogues and only one 7-methoxy analogue (**19f**) exhibited hemolytic properties. Taken together, the intrinsic antimicrobial activities and cytotoxic/hemolysis results successfully identified ten analogues (**14b** (H 3-6-3), **15b** (5-Br 3-6-3), **17c** (5-Me 3-7-3), **18a** (7-F 3-4-3), **18b** (7-F 3-6-3), **18d** (7-F 3-8-3), **19b** (7-OMe 3-6-3), **19e** (7-OMe 3-10-3), **20c** (7-Me 3-7-3) and **20d** (7-Me 3-8-3)) as being in vitro non-toxic antimicrobials with activity directed towards MRSA and *C. neoformans*, while the 5-bromo analogue **15c** (5-Br 3-7-3) and 5-methoxy analogues **16d** (5-OMe 3-8-3), **16e** (5-OMe 3-10-3), **16f** (5-OMe 3-12-3) exhibited a slightly broadened spectrum of activity that also included inhibition of the Gram-negative bacterium *E. coli*.

**Figure 5:**
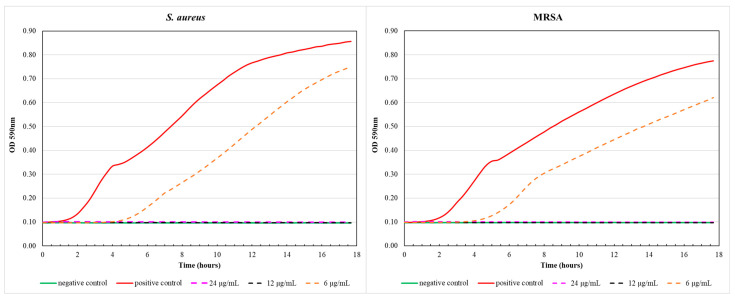
Bacterial growth inhibition exhibited by **15c** against *S. aureus* ATCC 25923 (**left**) and MRSA (CF-Marseille) (**right**) with different concentrations. Positive control was bacteria only and negative control was media only.

We next assessed the kinetics of antibacterial activity of **15c** towards the Gram-positive bacteria *S. aureus* ATCC 25923 and MRSA (CF-Marseille) [22] by measuring real time growth inhibition curves. The test compound completely inhibited both strains at 25.4 μM and 12.7 μM, whereas at the lowest tested concentration, 6.4 μM, bacterial growth was detected after 4 h (Figure 5). Classical microdilution methodology determined an MIC value of 12.7 μM for **15c** towards these two microorganisms, with the values matching those observed at 18 h in the real time growth inhibition curve plots. The same values were observed for the minimum bactericidal concentration (MBC) for **15c** against the two organisms, identifying this analogue as being bactericidal.

The compound set was next evaluated for the ability to enhance the antibiotic action of doxycycline towards *P. aeruginosa* ATCC 27853 and the action of erythromycin against *E. coli* ATCC 25922 (Table 3). In the case of the latter drug-microbe combination, the best result was observed for the 7-fluoro analogue **18a** with a modest four-fold enhancement of activity compared to the antibiotic alone. In contrast, three analogues were observed to potentiate the action of doxycycline against *P. aeruginosa* with greater than ten-fold enhancement—**15c** (18-fold), **16c** (>16-fold) and **16d** (16-fold).

## 4. Conclusions

Herein we have presented the latest results in our search for new α,ω-disubstituted indole-3-acetamido polyamine conjugates as antimicrobials and antibiotic enhancers. The capping acid that was the focus of this study, indole-3-acetic acid, was selected based upon our previous reported observation that a 5-bromoindole-3-acetamido-spermine conjugate exhibited antibiotic enhancement properties. We synthesized 39 new analogues, evaluating them for intrinsic antimicrobial activities as well as the ability to enhance the action of doxycycline against *P. aeruginosa* and erythromycin against *E. coli*. The results of our study revealed that many of these new compounds demonstrated remarkable potency against Gram-positive bacteria, specifically MRSA, as well as a fungal strain (*C. neoformans*). Among these compounds, a particular subset consisting of one 5-bromo analogue (**15c**) and three 5-methoxy analogues (**16d**–**f**) also displayed significant activity against the Gram-negative bacterium *E. coli*. Overall, we observed that compounds containing medium-length polyamine chains (3-6-3, 3-7-3, and 3-8-3) with a substituent on the 7-position of the indole tended to exhibit favorable activity against MRSA and *C. neoformans* with minimal to no cytotoxic activity. On the other hand, compounds bearing a 5-OMe substituent on the indole demonstrated a broader range of activity against different microbial targets. While the compound series was essentially unable to enhance the action of the lipophilic antibiotic erythromycin towards *E. coli*, three derivatives (**15c**, **16c**, **16d**) were found to enhance the action of doxycycline against *P. aeruginosa* with 16–18-fold enhancements. Collectively these results demonstrate the potential of this new series of compounds, suggesting that further efforts at activity optimization may well lead to viable candidates for in vivo evaluation.

## Figures and Tables

**Table 1:** Antimicrobial activities (MIC, µM) of analogues **14**–**20**.

Compound	MIC (µM)							
	*S. a* ^a^	*MRSA* ^b^	*P. a* ^c^	*E. c* ^d^	*K. p* ^e^	*A. b* ^f^	*C. a* ^g^	*C. n* ^h^
**14a** ^i^	200	>62	>200	>200 ^j^	>62	>62	>62	>62
**14b**	129	≤0.32	>259	>129	n.t. ^j^	n.t. ^j^	>41	≤0.32
**14c**	15.9	≤0.32	>254	>254	>41	>41	>41	≤0.32
**14d**	31.2	≤0.31	>250	62.4	>40	>40	>40	≤0.31
**14e**	3.77	≤0.30	60.3	15.1	>39	>39	≤0.30	≤0.30
**14f**	3.65	≤0.29	58.3	29.2	>37	>37	≤0.29	≤0.29
**15a** ^i^	25	47.4	200	200	>48	>48	>48	11.9
**15b**	13.4	≤0.27	215	26.9	>34	>34	2.15	≤0.27
**15c**	12.7	≤0.26	106	13.2	>34	>34	≤0.26	≤0.26
**15d**	13.0	≤0.26	>209	26.1	>33	>33	2.09	≤0.26
**15e**	6.33	≤0.25	50.7	12.7	>32	>32	≤0.25	≤0.25
**15f**	6.16	≤0.25	197	12.3	>32	7.88	≤0.25	≤0.25
**16a** ^i^	200	>55	>200	>200	>55	>55	>55	>55
**16b**	50	38	>200	100	>38	>38	>38	≤0.30
**16c**	>200	>38	>200	25	>38	>38	>38	2.36
**16d**	25	2.32	100	6.25	>37	>37	>37	≤0.29
**16e**	7.03	≤0.28	112	14.1	>36	>36	2.25	≤0.28
**16f**	6.82	≤0.27	109	13.6	>35	>35	35	≤0.27
**17a**	129	≤0.32	>259	>259	>41	>41	20.7	≤0.32
**17b**	31.2	≤0.31	>250	250	>40	>40	40.0	≤0.31
**17c**	61.4	≤0.31	>245	245	>39	>39	39.3	≤0.31
**17d**	15.1	≤0.30	>241	241	n.t. ^j^	n.t. ^j^	2.41	≤0.30
**17e**	7.29	≤0.29	233	58.3	>37	37	≤0.29	≤0.29
**17f**	7.06	≤0.28	226	14.1	>36	36	≤0.28	≤0.28
**18a**	16.0	≤0.32	>256	256	>41	>41	5.12	≤0.32
**18b**	124	≤0.31	>247	>247	>40	>40	39.6	≤0.31
**18c**	30.4	≤0.30	>243	>243	n.t. ^j^	n.t. ^j^	38.9	≤0.30
**18d**	29.9	≤0.30	>239	>239	n.t. ^j^	n.t. ^j^	>38	≤0.30
**18e**	7.23	≤0.29	>231	116	n.t. ^j^	n.t. ^j^	2.31	≤0.29
**18f**	7.00	≤0.28	224	28.0	n.t. ^j^	n.t. ^j^	≤0.28	≤0.28
**19a**	124	19.9	>249	>249	n.t. ^j^	n.t. ^j^	>40	19.9
**19b**	30.0	≤0.30	>240	240	n.t. ^j^	n.t. ^j^	>38	≤0.30
**19c**	29.5	37.8	>236	236	>38	>38	>38	≤0.30
**19d**	116	37.2	>232	>232	n.t. ^j^	n.t. ^j^	>37	≤0.29
**19e**	28.1	≤0.28	>225	>225	n.t. ^j^	n.t. ^j^	>36	≤0.28
**19f**	13.6	≤0.27	>218	109	n.t. ^j^	n.t. ^j^	34.9	≤0.27
**20a**	32.3	≤0.32	>259	259	n.t. ^j^	n.t. ^j^	41.4	≤0.32
**20b**	62.4	≤0.31	>250	250	n.t. ^j^	n.t. ^j^	40.0	≤0.31
**20c**	3.83	≤0.31	>245	245	>39	>39	>39	≤0.31
**20d**	30.2	≤0.30	>241	241	n.t. ^j^	n.t. ^j^	38.6	≤0.30
**20e**	233	≤0.29	>233	>233	n.t. ^j^	n.t. ^j^	18.7	≤0.29
**20f**	28.2	≤0.28	>226	56.5	n.t. ^j^	n.t. ^j^	≤0.28	≤0.28

^a^ *S. aureus* ATCC 25923 with streptomycin (MIC 21.5 μM) and chloramphenicol (MIC 1.5–3 μM) as positive controls and values presented as the mean (n = 3); ^b^ MRSA ATCC 43300 with vancomycin (MIC 0.7 μM) used as the positive control and values presented as the mean (n = 2); ^c^ *P. aeruginosa* ATCC 27853 with streptomycin (MIC 21.5 μM) and colistin (MIC 1 μM) as positive controls and values presented as the mean (n = 3); ^d^ *E. coli* ATCC 25922 with streptomycin (MIC 21.5 μM) and colistin (MIC 2 μM) as positive controls and values presented as the mean (n = 3); ^e^ *K. pneumoniae* ATCC 700603 with values presented as the mean (n = 2); ^f^ *A. baumannii* ATCC 19606 with colistin (MIC 0.2 μM) as the positive control and values presented as the mean (n = 2); ^g^ *C. albicans* ATCC 90028 with fluconazole (MIC 0.4 μM) as the positive control and values presented as the mean (n = 2); ^h^ *C. neoformans* ATCC 208821 with fluconazole (MIC 26 μM) as the positive control and values presented as the mean (n = 2); ^i^ Data taken from Cadelis et al. [15]; ^j^ Not tested.

**Table 2:** Cytotoxic (IC_50_, µM) and hemolytic (HC_10_, µM) properties of analogues **14**–**20**.

Compound	Cytotoxicity ^a^	Hemolysis ^b^	Compound	Cytotoxicity ^a^	Hemolysis ^b^
**14a** ^c^	>43	>43	**18a**	>41	>41
**14b**	>41	>41	**18b**	>40	>40
**14c**	>41	0.72	**18c**	>39	1.94
**14d**	>40	15.8	**18d**	>38	>38
**14e**	>39	≤0.30	**18e**	>37	1.49
**14f**	>37	≤0.29	**18f**	27.2	≤0.28
**15a** ^c^	>35	>35	**19a**	>40	>40
**15b**	>34	>34	**19b**	>38	n.t. ^d^
**15c**	>34	>34	**19c**	>38	>38
**15d**	>33	10.1	**19d**	>37	>37
**15e**	>32	≤0.25	**19e**	>36	n.t.^d^
**15f**	4.75	≤0.25	**19f**	>35	10.7
**16a** ^c^	>40	>40	**20a**	>41	4.11
**16b**	>38	>38	**20b**	>40	9.24
**16c**	>38	>38	**20c**	>39	n.t. ^d^
**16d**	>37	>37	**20d**	>39	n.t. ^d^
**16e**	>36	>36	**20e**	>37	12.7
**16f**	>35	>35	**20f**	>36	≤0.28
**17a**	>41	8.46			
**17b**	>40	23.1			
**17c**	>39	>39			
**17d**	>39	0.42			
**17e**	>37	3.19			
**17f**	>36	≤0.28			

All values presented as the mean (n = 2); ^a^ Concentration of compound at 50% cytotoxicity on HEK293 (human embryonic kidney) cells with tamoxifen as the positive control (IC_50_ 24 μM); ^b^ Concentration of compound at 10% hemolytic activity on human red blood cells with melittin as the positive control (HC_10_ 0.95 μM); ^c^ Data taken from Cadelis et al. [15]; ^d^ Not tested.

**Table 3:** Antibiotic enhancement activity (MIC, µM) of analogues **14**–**20**.

Compound	Dox/*P. a* ^a^	Eryth/*E. c* ^b^	Compound	Dox/*P. a* ^a^	Eryth/*E. c* ^b^
**14a** ^c^	50 (>4)	n.t. ^d^	**18a**	64.0 (4)	64.0 (4)
**14b**	129 (>2)	>259 (0.5)	**18b**	247 (>1)	>247 (1)
**14c**	63.5 (4)	>254 (1)	**18c**	243 (>1)	243 (>1)
**14d**	250 (>1)	250 (0.25)	**18d**	>239 (1)	>239 (1)
**14e**	15.1 (4)	15.1 (1)	**18e**	231 (1)	57.8 (2)
**14f**	14.6 (4)	14.6 (2)	**18f**	224 (1)	14.0 (2)
**15a** ^c^	6.25 (32)	n.t. ^d^	**19a**	>249 (1)	>249 (1)
**15b**	26.9 (8)	53.7 (0.5)	**19b**	>240 (1)	240 (1)
**15c**	6.62 (18)	6.62 (2)	**19c**	118 (>2)	236 (1)
**15d**	52.2 (>4)	52.2 (0.5)	**19d**	>232 (1)	>232 (1)
**15e**	12.7 (4)	12.7 (1)	**19e**	225 (>1)	225 (>1)
**15f**	98.5 (2)	24.6 (0.5)	**19f**	218 (>1)	109 (1)
**16a** ^c^	200 (>1)	n.t. ^d^	**20a**	>259 (1)	64.7 (4)
**16b**	25 (>8)	50 (2)	**20b**	250 (>1)	62.4 (4)
**16c**	12.5 (>16)	50 (0.5)	**20c**	123 (>2)	123 (2)
**16d**	6.25 (16)	6.25 (1)	**20d**	>241 (1)	241 (1)
**16e**	14.1 (8)	28.1 (0.5)	**20e**	>233 (1)	>233 (1)
**16f**	13.6 (8)	27.3 (0.5)	**20f**	>226 (1)	28.2 (2)
**17a**	129 (>2)	64.7 (4)			
**17b**	31.2 (>8)	31.2 (8)			
**17c**	123 (>2)	61.4 (4)			
**17d**	241 (>1)	121 (2)			
**17e**	233 (1)	29.2 (2)			
**17f**	226 (1)	14.1 (1)			

^a^ Concentration (µM) required to restore doxycycline activity at 4.5 µM against *P. aeruginosa* ATCC 27853. Fold change shown in parentheses is the ratio between the intrinsic MIC of the test compound and the combination MIC; ^b^ Concentration (µM) required to restore erythromycin activity at 10.9 µM against *E. coli* ATCC 25922. Fold change shown in parentheses is the ratio between the intrinsic MIC of the test compound and the combination MIC; ^c^ Data taken from Cadelis et al. [15]; ^d^ Not tested.

## Data Availability

Data are contained within the article or Supplementary Materials.

## Supplementary Materials

The following supporting information can be downloaded at: https://www.mdpi.com/article/10.3390/biom13081226/s1, Figure S1: ^1^H (CD_3_OD, 400 MHz) and ^13^C (CD_3_OD, 100 MHz) NMR spectra for **14b**; Figure S2: ^1^H (CD_3_OD, 400 MHz) and ^13^C (CD_3_OD, 100 MHz) NMR spectra for **14c**; Figure S3: ^1^H (CD_3_OD, 400 MHz) and ^13^C (CD_3_OD, 100 MHz) NMR spectra for **14d**; Figure S4: ^1^H (CD_3_OD, 400 MHz) and ^13^C (CD_3_OD, 100 MHz) NMR spectra for **14e**; Figure S5: ^1^H (CD_3_OD, 400 MHz) and ^13^C (CD_3_OD, 100 MHz) NMR spectra for **14f**; Figure S6: ^1^H (CD_3_OD, 400 MHz) and ^13^C (CD_3_OD, 100 MHz) NMR spectra for **15b**; Figure S7: ^1^H (CD_3_OD, 400 MHz) and ^13^C (CD_3_OD, 100 MHz) NMR spectra for **15c**; Figure S8: ^1^H (CD_3_OD, 400 MHz) and ^13^C (CD_3_OD, 100 MHz) NMR spectra for **15d**; Figure S9: ^1^H (CD_3_OD, 400 MHz) and ^13^C (CD_3_OD, 100 MHz) NMR spectra for **15e**; Figure S10: ^1^H (CD_3_OD, 400 MHz) and ^13^C (CD_3_OD, 100 MHz) NMR spectra for **15f**; Figure S11: ^1^H (CD_3_OD, 400 MHz) and ^13^C (CD_3_OD, 100 MHz) NMR spectra for **16b**; Figure S12: ^1^H (CD_3_OD, 400 MHz) and ^13^C (CD_3_OD, 100 MHz) NMR spectra for **16c**; Figure S13: ^1^H (CD_3_OD, 400 MHz) and ^13^C (CD_3_OD, 100 MHz) NMR spectra for **16d**; Figure S14: ^1^H (CD_3_OD, 400 MHz) and ^13^C (CD_3_OD, 100 MHz) NMR spectra for **16e**; Figure S15: ^1^H (CD_3_OD, 400 MHz) and ^13^C (CD_3_OD, 100 MHz) NMR spectra for **16f**; Figure S16: ^1^H (CD_3_OD, 400 MHz) and ^13^C (CD_3_OD, 100 MHz) NMR spectra for **17a**; Figure S17: ^1^H (CD_3_OD, 400 MHz) and ^13^C (CD_3_OD, 100 MHz) NMR spectra for **17b**; Figure S18: ^1^H (CD_3_OD, 400 MHz) and ^13^C (CD_3_OD, 100 MHz) NMR spectra for **17c**; Figure S19: ^1^H (CD_3_OD, 400 MHz) and ^13^C (CD_3_OD, 100 MHz) NMR spectra for **17d**; Figure S20: ^1^H (CD_3_OD, 400 MHz) and ^13^C (CD_3_OD, 100 MHz) NMR spectra for **17e**; Figure S21: ^1^H (CD_3_OD, 400 MHz) and ^13^C (CD_3_OD, 100 MHz) NMR spectra for **17f**; Figure S22: ^1^H (CD_3_OD, 400 MHz) and ^13^C (CD_3_OD, 100 MHz) NMR spectra for **18a**; Figure S23: ^1^H (CD_3_OD, 400 MHz) and ^13^C (CD_3_OD, 100 MHz) NMR spectra for **18b**; Figure S24: ^1^H (CD_3_OD, 400 MHz) and ^13^C (CD_3_OD, 100 MHz) NMR spectra for **18c**; Figure S25: ^1^H (CD_3_OD, 400 MHz) and ^13^C (CD_3_OD, 100 MHz) NMR spectra for **18d**; Figure S26: ^1^H (CD_3_OD, 400 MHz) and ^13^C (CD_3_OD, 100 MHz) NMR spectra for **18e**; Figure S27: ^1^H (CD_3_OD, 400 MHz) and ^13^C (CD_3_OD, 100 MHz) NMR spectra for **18f**; Figure S28: ^1^H (CD_3_OD, 400 MHz) and ^13^C (CD_3_OD, 100 MHz) NMR spectra for **19a**; Figure S29: ^1^H (CD_3_OD, 400 MHz) and ^13^C (CD_3_OD, 100 MHz) NMR spectra for **19b**; Figure S30: ^1^H (CD_3_OD, 400 MHz) and ^13^C (CD_3_OD, 100 MHz) NMR spectra for **19c**; Figure S31: ^1^H (CD_3_OD, 400 MHz) and ^13^C (CD_3_OD, 100 MHz) NMR spectra for **19d**; Figure S32: ^1^H (CD_3_OD, 400 MHz) and ^13^C (CD_3_OD, 100 MHz) NMR spectra for **19e**; Figure S33: ^1^H (CD_3_OD, 400 MHz) and ^13^C (CD_3_OD, 100 MHz) NMR spectra for **19f**; Figure S34: ^1^H (CD_3_OD, 400 MHz) and ^13^C (CD_3_OD, 100 MHz) NMR spectra for **20a**; Figure S35: ^1^H (CD_3_OD, 400 MHz) and ^13^C (CD_3_OD, 100 MHz) NMR spectra for **20b**; Figure S36: ^1^H (CD_3_OD, 400 MHz) and ^13^C (CD_3_OD, 100 MHz) NMR spectra for **20c**; Figure S37: ^1^H (CD_3_OD, 400 MHz) and ^13^C (CD_3_OD, 100 MHz) NMR spectra for **20d**; Figure S38: ^1^H (CD_3_OD, 400 MHz) and ^13^C (CD_3_OD, 100 MHz) NMR spectra for **20e**; Figure S39: ^1^H (CD_3_OD, 400 MHz) and ^13^C (CD_3_OD, 100 MHz) NMR spectra for **20f**.

## References

[1] Payne D.J., Gwynn M.N., Holmes D.J., Pompliano D.L. (2007). Drugs for Bad Bugs: Confronting the Challenges of Antibacterial Discovery. Nat. Rev. Drug Discov..

[2] Bernal P., Molina-Santiago C., Daddaoua A., Llamas M.A. (2013). Antibiotic Adjuvants: Identification and Clinical Use: Antibiotic Adjuvants. Microb. Biotechnol..

[3] Farha M.A., Brown E.D. (2013). Discovery of Antibiotic Adjuvants. Nat. Biotechnol..

[4] Melander R.J., Melander C. (2017). The Challenge of Overcoming Antibiotic Resistance: An Adjuvant Approach?. ACS Infect. Dis..

[5] Dhanda G., Acharya Y., Haldar J. (2023). Antibiotic Adjuvants: A Versatile Approach to Combat Antibiotic Resistance. ACS Omega.

[6] Douafer H., Andrieu V., Phanstiel O., Brunel J.M. (2019). Antibiotic Adjuvants: Make Antibiotics Great Again!. J. Med. Chem..

[7] Bush K., Bradford P.A. (2016). β-Lactams and β-Lactamase Inhibitors: An Overview. Cold Spring Harb. Perspect. Med..

[8] Zurawski D.V., Reinhart A.A., Alamneh Y.A., Pucci M.J., Si Y., Abu-Taleb R., Shearer J.P., Demons S.T., Tyner S.D., Lister T. (2017). SPR741, an Antibiotic Adjuvant, Potentiates the In Vitro and In Vivo Activity of Rifampin against Clinically Relevant Extensively Drug-Resistant Acinetobacter Baumannii. Antimicrob. Agents Chemother..

[9] Konai M.M., Haldar J. (2020). Lysine-Based Small Molecule Sensitizes Rifampicin and Tetracycline against Multidrug-Resistant *Acinetobacter Baumannii* and *Pseudomonas Aeruginosa*. ACS Infect. Dis..

[10] Pieri C., Borselli D., Di Giorgio C., De Méo M., Bolla J.-M., Vidal N., Combes S., Brunel J.M. (2014). New Ianthelliformisamine Derivatives as Antibiotic Enhancers against Resistant Gram-Negative Bacteria. J. Med. Chem..

[11] Li S.A., Cadelis M.M., Sue K., Blanchet M., Vidal N., Brunel J.M., Bourguet-Kondracki M.-L., Copp B.R. (2019). 6-Bromoindolglyoxylamido Derivatives as Antimicrobial Agents and Antibiotic Enhancers. Bioorg. Med. Chem..

[12] Cadelis M.M., Liu T., Sue K., Rouvier F., Bourguet-Kondracki M.-L., Brunel J.M., Copp B.R. (2023). Structure–Activity Relationship Studies of Indolglyoxyl-Polyamine Conjugates as Antimicrobials and Antibiotic Potentiators. Pharmaceuticals.

[13] James D.A., Koya K., Li H., Liang G., Xia Z., Ying W., Wu Y., Sun L. (2008). Indole- and Indolizine-Glyoxylamides Displaying Cytotoxicity against Multidrug Resistant Cancer Cell Lines. Bioorg. Med. Chem. Lett..

[14] Colley H.E., Muthana M., Danson S.J., Jackson L.V., Brett M.L., Harrison J., Coole S.F., Mason D.P., Jennings L.R., Wong M. (2015). An Orally Bioavailable, Indole-3-Glyoxylamide Based Series of Tubulin Polymerization Inhibitors Showing Tumor Growth Inhibition in a Mouse Xenograft Model of Head and Neck Cancer. J. Med. Chem..

[15] Cadelis M.M., Li S.A., Bourguet-Kondracki M., Blanchet M., Douafer H., Brunel J.M., Copp B.R. (2021). Spermine Derivatives of Indole-3-carboxylic Acid, Indole-3-acetic Acid and Indole-3-acrylic Acid as Gram-Negative Antibiotic Adjuvants. ChemMedChem.

[16] Cadelis M.M., Pike E.I.W., Kang W., Wu Z., Bourguet-Kondracki M.-L., Blanchet M., Vidal N., Brunel J.M., Copp B.R. (2019). Exploration of the Antibiotic Potentiating Activity of Indolglyoxylpolyamines. Eur. J. Med. Chem..

[17] Pearce A.N., Kaiser M., Copp B.R. (2017). Synthesis and Antimalarial Evaluation of Artesunate-Polyamine and Trioxolane-Polyamine Conjugates. Eur. J. Med. Chem..

[18] Klenke B., Gilbert I.H. (2001). Nitrile Reduction in the Presence of Boc-Protected Amino Groups by Catalytic Hydrogenation over Palladium-Activated Raney-Nickel. J. Org. Chem..

[19] Klenke B., Stewart M., Barrett M.P., Brun R., Gilbert I.H. (2001). Synthesis and Biological Evaluation of s-Triazine Substituted Polyamines as Potential New Anti-Trypanosomal Drugs. J. Med. Chem..

[20] Israel M., Rosenfield J.S., Modest E.J. (1964). Analogs of Spermine and Spermidine. I. Synthesis of Polymethylenepolyamines by Reduction of Cyanoethylated α,ι-Alkylenediamines1,2. J. Med. Chem..

[21] Blaskovich M.A.T., Zuegg J., Elliott A.G., Cooper M.A. (2015). Helping Chemists Discover New Antibiotics. ACS Infect. Dis..

[22] Rolain J.-M., Francois P., Hernandez D., Bittar F., Richet H., Fournous G., Mattenberger Y., Bosdure E., Stremler N., Dubus J.-C. (2009). Genomic Analysis of an Emerging Multiresistant Staphylococcus Aureus Strain Rapidly Spreading in Cystic Fibrosis Patients Revealed the Presence of an Antibiotic Inducible Bacteriophage. Biol. Direct.

