# Supplementary material for: Indole-3-Acetamido-Polyamines as Antimicrobial Agents and Antibiotic Adjuvants
Source: Biomolecules. 2023 Aug 7;13(8):1226. doi: 10.3390/biom13081226 (PMC10452110; doi:10.3390/biom13081226)
Supplement: Supplementary file 1 [file biomolecules-13-01226-s001.zip › biomolecules-2505375-supplementary.pdf]

Supporting Information

Indole Acetamido-Polyamines as Antimicrobial Agents and Antibiotic Adjuvants

Kenneth Sue[1], Melissa M. Cadelis[1,2], Evangelene S. Gill[1], Florent Rouvier[3], Marie-Lise Bourguet-Kondracki[4], Jean Michel Brunel[3] and Brent R. Copp[1,*]

[1] School of Chemical Sciences, The University of Auckland, Private Bag 92019, Auckland 1142, New Zealand

[2] School of Medical Sciences, The University of Auckland, Private Bag 92019, Auckland 1142, New Zealand

[3] Membranes et Cibles Thérapeutiques, INSERM, Aix-Marseille Universite, 27 bd Jean Moulin, 13385 Marseille, France

[4] Laboratoire Molécules de Communication et Adaptation des Micro-organismes, UMR 7245 CNRS, Muséum National d'Histoire Naturelle, 57 rue Cuvier (C.P. 54), 75005 Paris, France

∗Corresponding author e-mail address: b.copp@auckland.ac.nz (Brent R. Copp).

Contents

Figure S1 ^{1}H (CD_3OD, 400 MHz) and ^{13}C (CD_3OD, 100 MHz) NMR spectra for **14b** **S3**
Figure S2 ^{1}H (CD_3OD, 400 MHz) and ^{13}C (CD_3OD, 100 MHz) NMR spectra for **14c** **S4**
Figure S3 ^{1}H (CD_3OD, 400 MHz) and ^{13}C (CD_3OD, 100 MHz) NMR spectra for **14d** **S5**
Figure S4 ^{1}H (CD_3OD, 400 MHz) and ^{13}C (CD_3OD, 100 MHz) NMR spectra for **14e** **S6**
Figure S5 ^{1}H (CD_3OD, 400 MHz) and ^{13}C (CD_3OD, 100 MHz) NMR spectra for **14f** **S7**
Figure S6 ^{1}H (CD_3OD, 400 MHz) and ^{13}C (CD_3OD, 100 MHz) NMR spectra for **15b** **S8**
Figure S7 ^{1}H (CD_3OD, 400 MHz) and ^{13}C (CD_3OD, 100 MHz) NMR spectra for **15c** **S9**
Figure S8 ^{1}H (CD_3OD, 400 MHz) and ^{13}C (CD_3OD, 100 MHz) NMR spectra for **15d** **S10**
Figure S9 ^{1}H (CD_3OD, 400 MHz) and ^{13}C (CD_3OD, 100 MHz) NMR spectra for **15e** **S11**
Figure S10 ^{1}H (CD_3OD, 400 MHz) and ^{13}C (CD_3OD, 100 MHz) NMR spectra for **15f** **S12**
Figure S11 ^{1}H (CD_3OD, 400 MHz) and ^{13}C (CD_3OD, 100 MHz) NMR spectra for **16b** **S13**
Figure S12 ^{1}H (CD_3OD, 400 MHz) and ^{13}C (CD_3OD, 100 MHz) NMR spectra for **16c** **S14**
Figure S13 ^{1}H (CD_3OD, 400 MHz) and ^{13}C (CD_3OD, 100 MHz) NMR spectra for **16d** **S15**
Figure S14 ^{1}H (CD_3OD, 400 MHz) and ^{13}C (CD_3OD, 100 MHz) NMR spectra for **16e** **S16**
Figure S15 ^{1}H (CD_3OD, 400 MHz) and ^{13}C (CD_3OD, 100 MHz) NMR spectra for **16f** **S17**
Figure S16 ^{1}H (CD_3OD, 400 MHz) and ^{13}C (CD_3OD, 100 MHz) NMR spectra for **17a** **S18**
Figure S17 ^{1}H (CD_3OD, 400 MHz) and ^{13}C (CD_3OD, 100 MHz) NMR spectra for **17b** **S19**
Figure S18 ^{1}H (CD_3OD, 400 MHz) and ^{13}C (CD_3OD, 100 MHz) NMR spectra for **17c** **S20**

Figure S19 ^{1}H (CD_3OD, 400 MHz) and ^{13}C (CD_3OD, 100 MHz) NMR spectra for **17d** **S21**
Figure S20 ^{1}H (CD_3OD, 400 MHz) and ^{13}C (CD_3OD, 100 MHz) NMR spectra for **17e** **S22**
Figure S21 ^{1}H (CD_3OD, 400 MHz) and ^{13}C (CD_3OD, 100 MHz) NMR spectra for **17f** **S23**
Figure S22 ^{1}H (CD_3OD, 400 MHz) and ^{13}C (CD_3OD, 100 MHz) NMR spectra for **18a** **S24**
Figure S23 ^{1}H (CD_3OD, 400 MHz) and ^{13}C (CD_3OD, 100 MHz) NMR spectra for **18b** **S25**
Figure S24 ^{1}H (CD_3OD, 400 MHz) and ^{13}C (CD_3OD, 100 MHz) NMR spectra for **18c** **S26**
Figure S25 ^{1}H (CD_3OD, 400 MHz) and ^{13}C (CD_3OD, 100 MHz) NMR spectra for **18d** **S27**
Figure S26 ^{1}H (CD_3OD, 400 MHz) and ^{13}C (CD_3OD, 100 MHz) NMR spectra for **18e** **S28**
Figure S27 ^{1}H (CD_3OD, 400 MHz) and ^{13}C (CD_3OD, 100 MHz) NMR spectra for **18f** **S29**
Figure S28 ^{1}H (CD_3OD, 400 MHz) and ^{13}C (CD_3OD, 100 MHz) NMR spectra for **19a** **S30**
Figure S29 ^{1}H (CD_3OD, 400 MHz) and ^{13}C (CD_3OD, 100 MHz) NMR spectra for **19b** **S31**
Figure S30 ^{1}H (CD_3OD, 400 MHz) and ^{13}C (CD_3OD, 100 MHz) NMR spectra for **19c** **S32**
Figure S31 ^{1}H (CD_3OD, 400 MHz) and ^{13}C (CD_3OD, 100 MHz) NMR spectra for **19d** **S33**
Figure S32 ^{1}H (CD_3OD, 400 MHz) and ^{13}C (CD_3OD, 100 MHz) NMR spectra for **19e** **S34**
Figure S33 ^{1}H (CD_3OD, 400 MHz) and ^{13}C (CD_3OD, 100 MHz) NMR spectra for **19f** **S35**
Figure S34 ^{1}H (CD_3OD, 400 MHz) and ^{13}C (CD_3OD, 100 MHz) NMR spectra for **20a** **S36**
Figure S35 ^{1}H (CD_3OD, 400 MHz) and ^{13}C (CD_3OD, 100 MHz) NMR spectra for **20b** **S37**
Figure S36 ^{1}H (CD_3OD, 400 MHz) and ^{13}C (CD_3OD, 100 MHz) NMR spectra for **20c** **S38**
Figure S37 ^{1}H (CD_3OD, 400 MHz) and ^{13}C (CD_3OD, 100 MHz) NMR spectra for **20d** **S39**
Figure S38 ^{1}H (CD_3OD, 400 MHz) and ^{13}C (CD_3OD, 100 MHz) NMR spectra for **20e** **S40**
Figure S39 ^{1}H (CD_3OD, 400 MHz) and ^{13}C (CD_3OD, 100 MHz) NMR spectra for **20f** **S41**

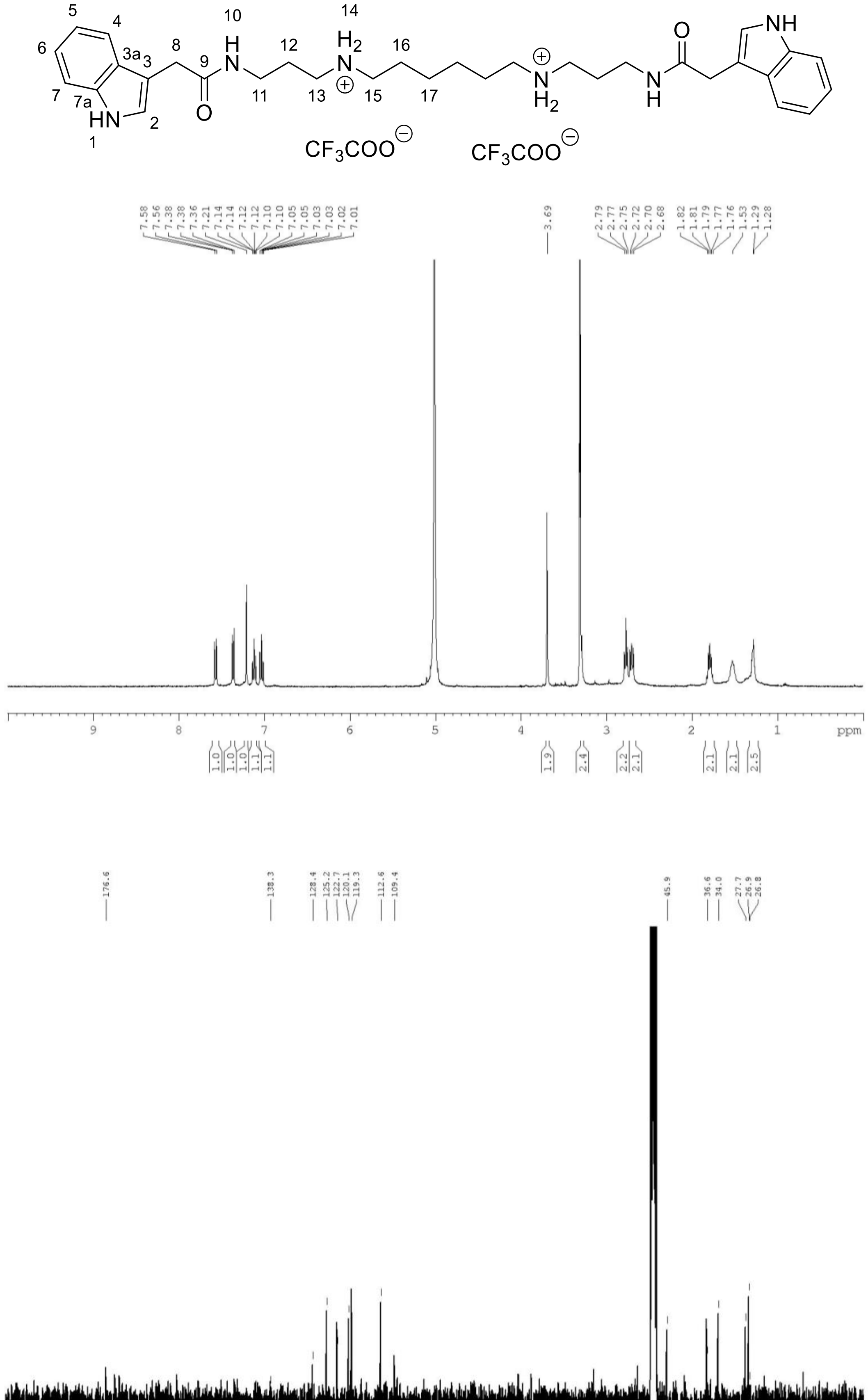

Figure S1 ^{1}H (CD_3OD, 400 MHz) and ^{13}C (CD_3OD, 100 MHz) NMR spectra for **14b**

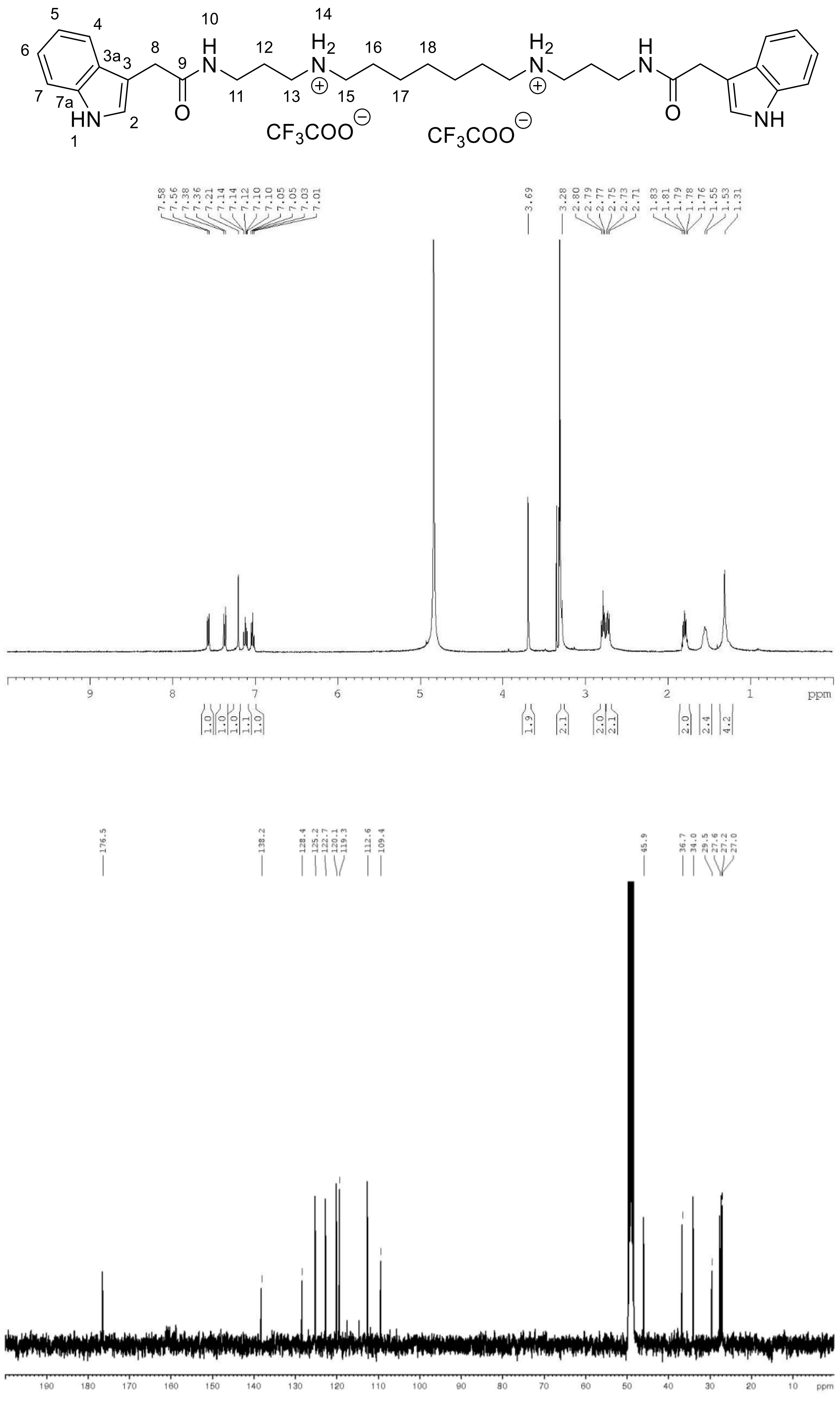

Figure S2 ^{1}H (CD_3OD, 400 MHz) and ^{13}C (CD_3OD, 100 MHz) NMR spectra for **14c**

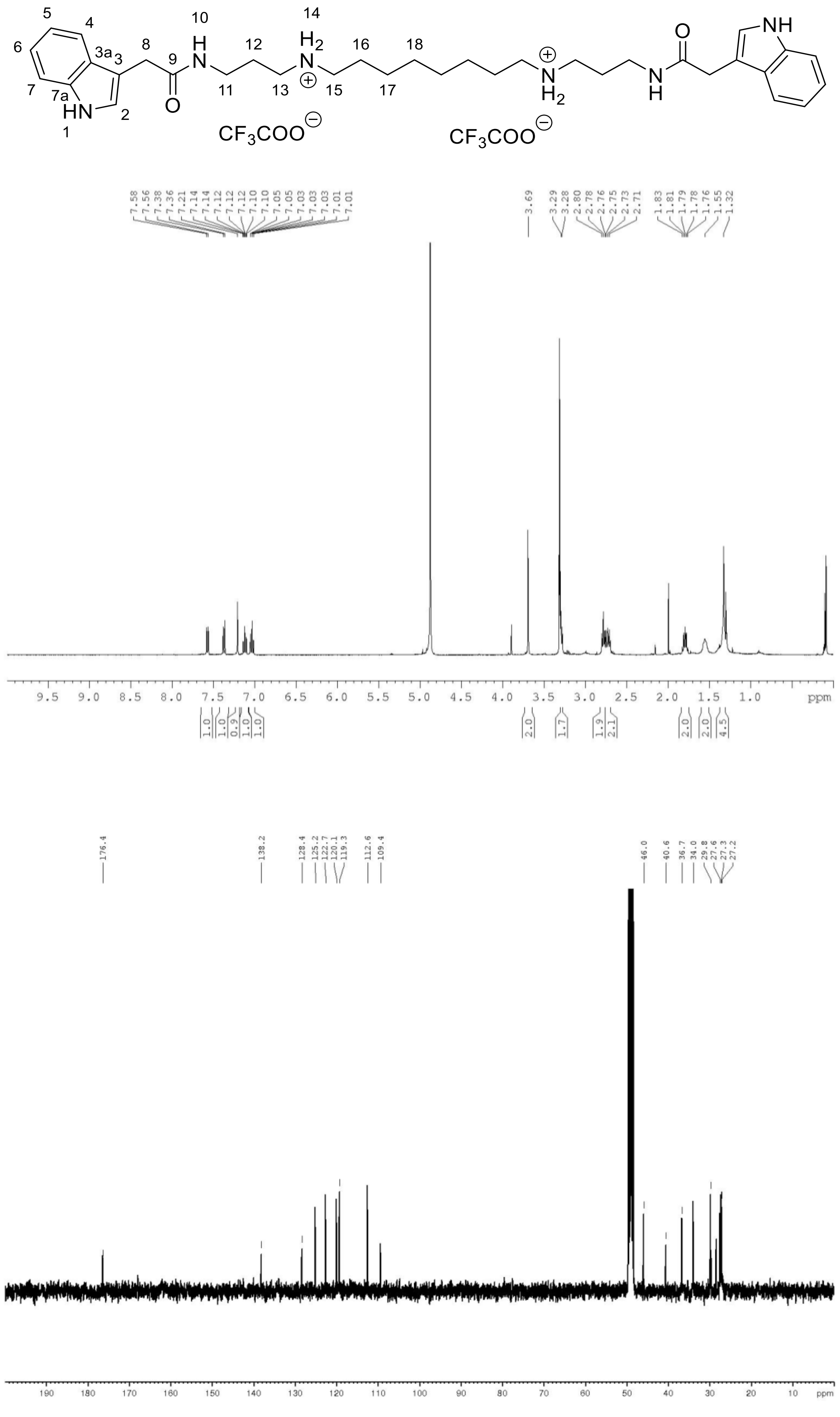

Figure S3 ${}^{1}H$ (CD_3OD, 400 MHz) and ${}^{13}C$ (CD_3OD, 100 MHz) NMR spectra for **14d**

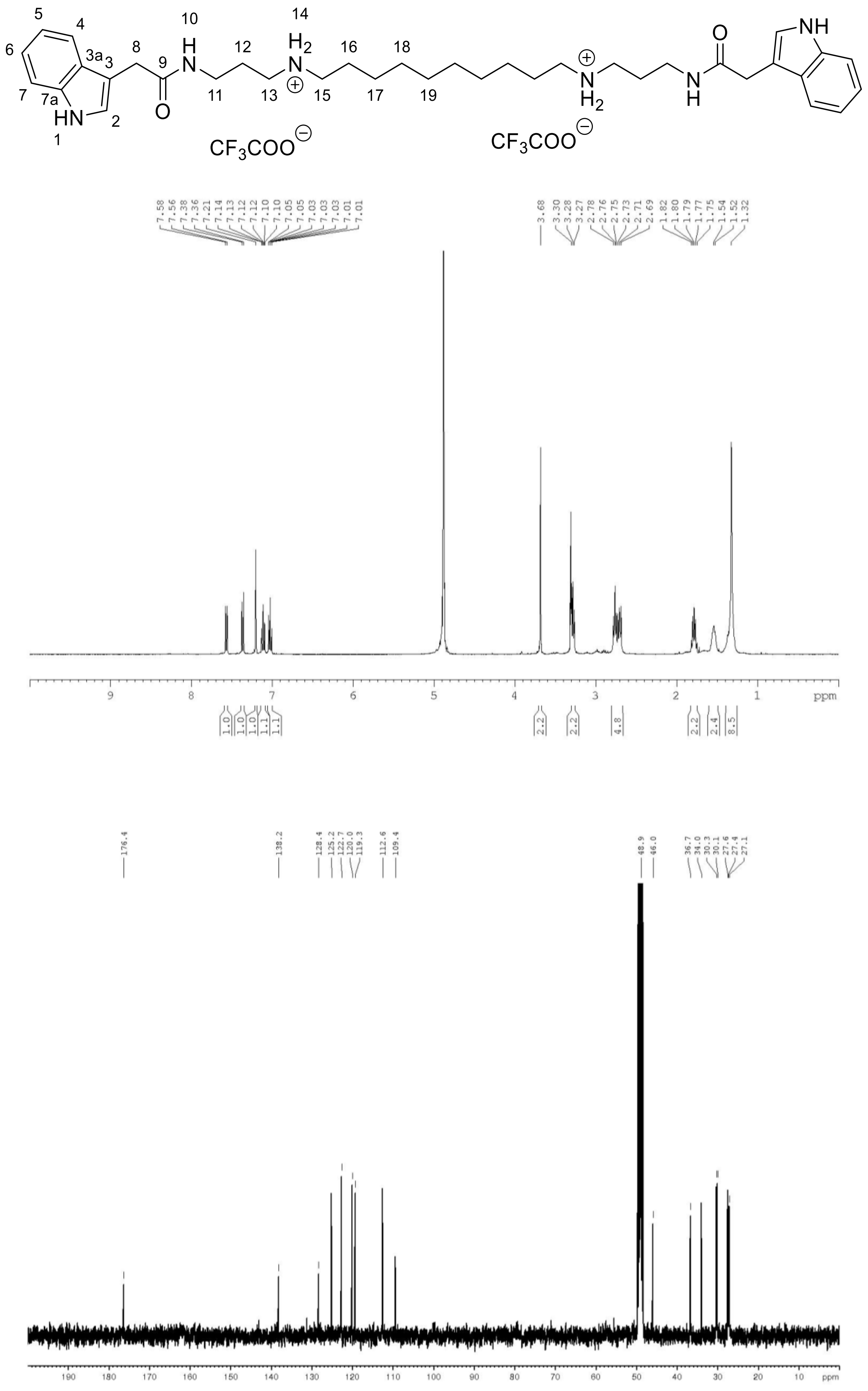

Figure S4 ^{1}H (CD_3OD, 400 MHz) and ^{13}C (CD_3OD, 100 MHz) NMR spectra for **14e**

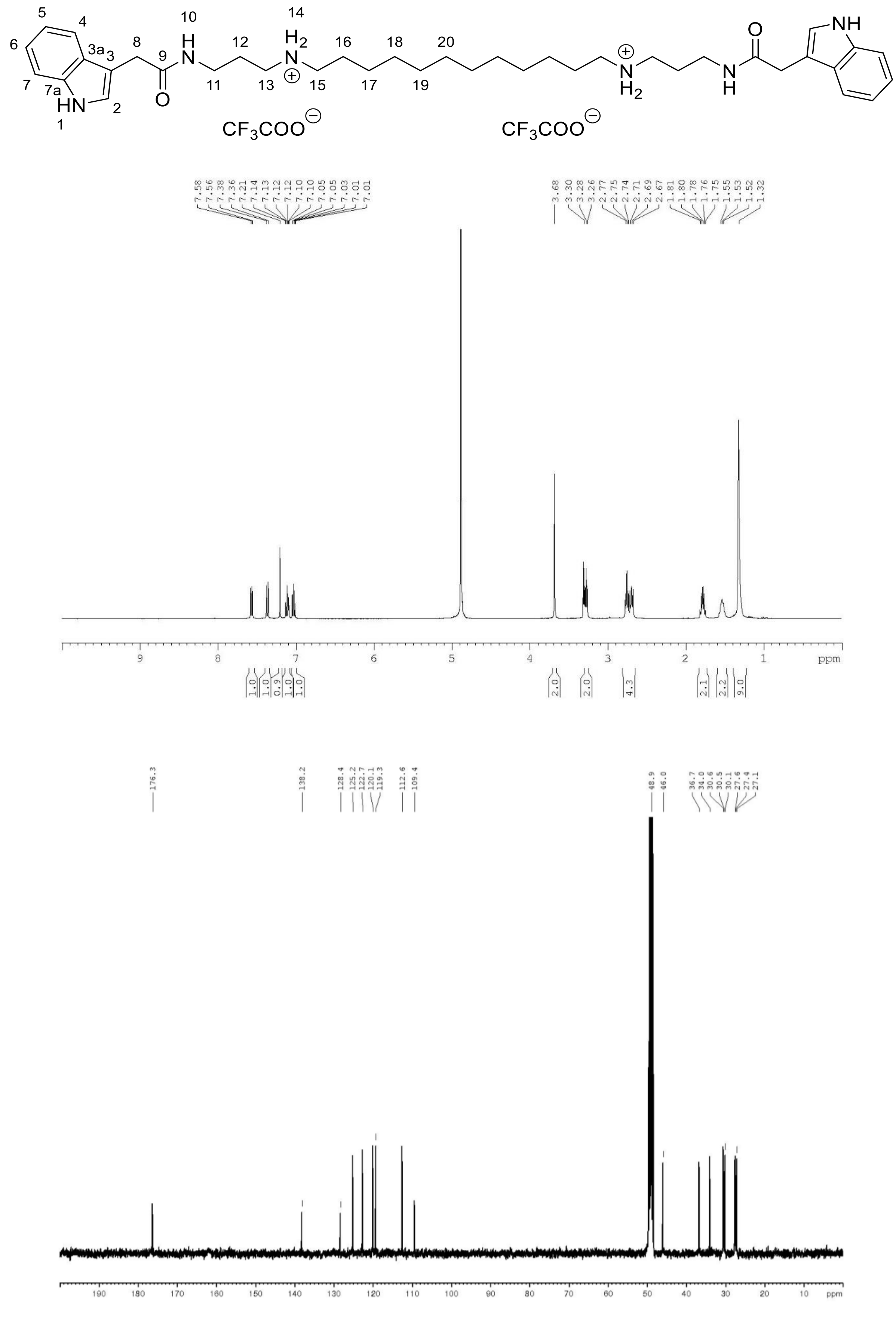

Figure S5 ^{1}H (CD_3OD, 400 MHz) and ^{13}C (CD_3OD, 100 MHz) NMR spectra for **14f**

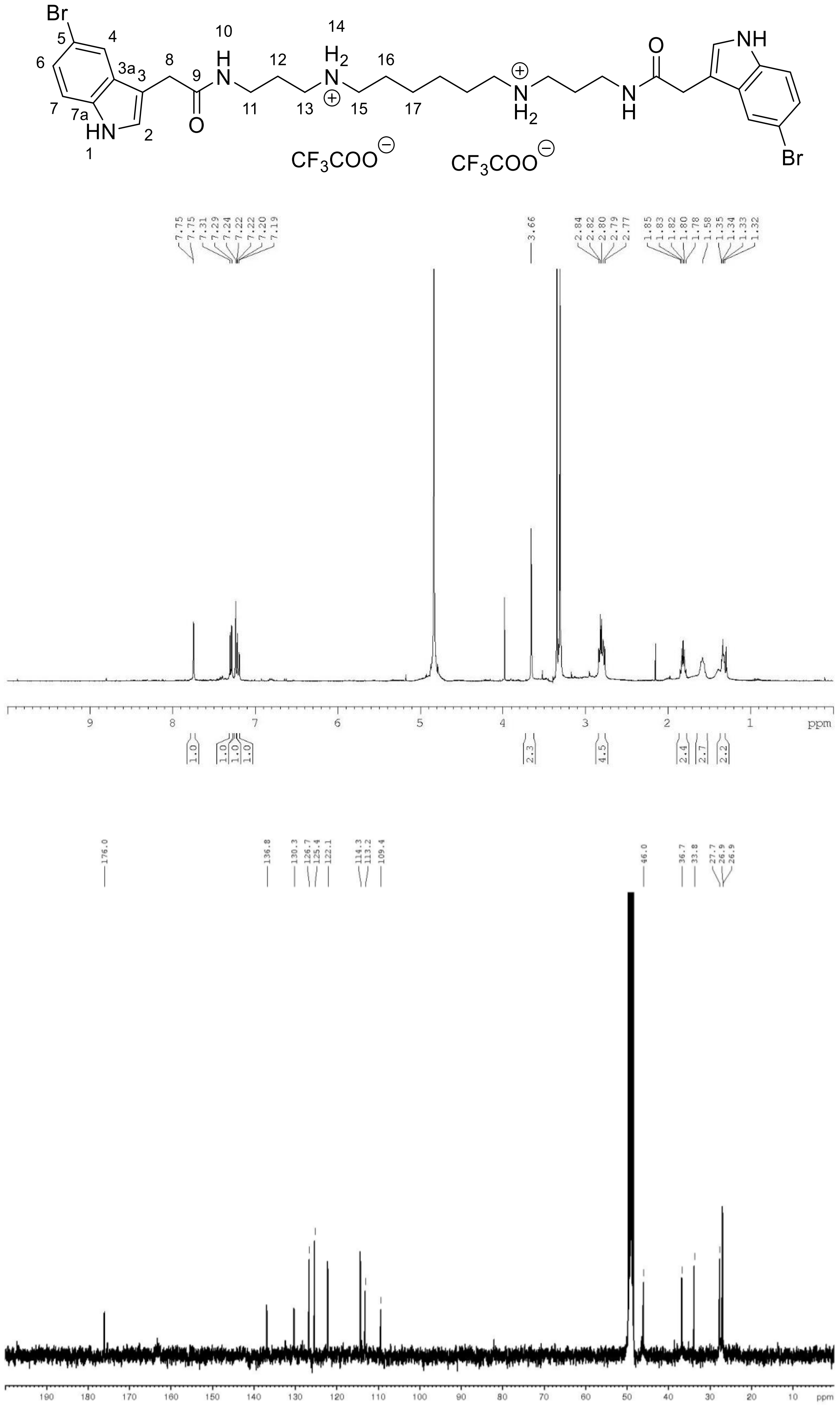

Figure S6 ^{1}H (CD_3OD, 400 MHz) and ^{13}C (CD_3OD, 100 MHz) NMR spectra for **15b**

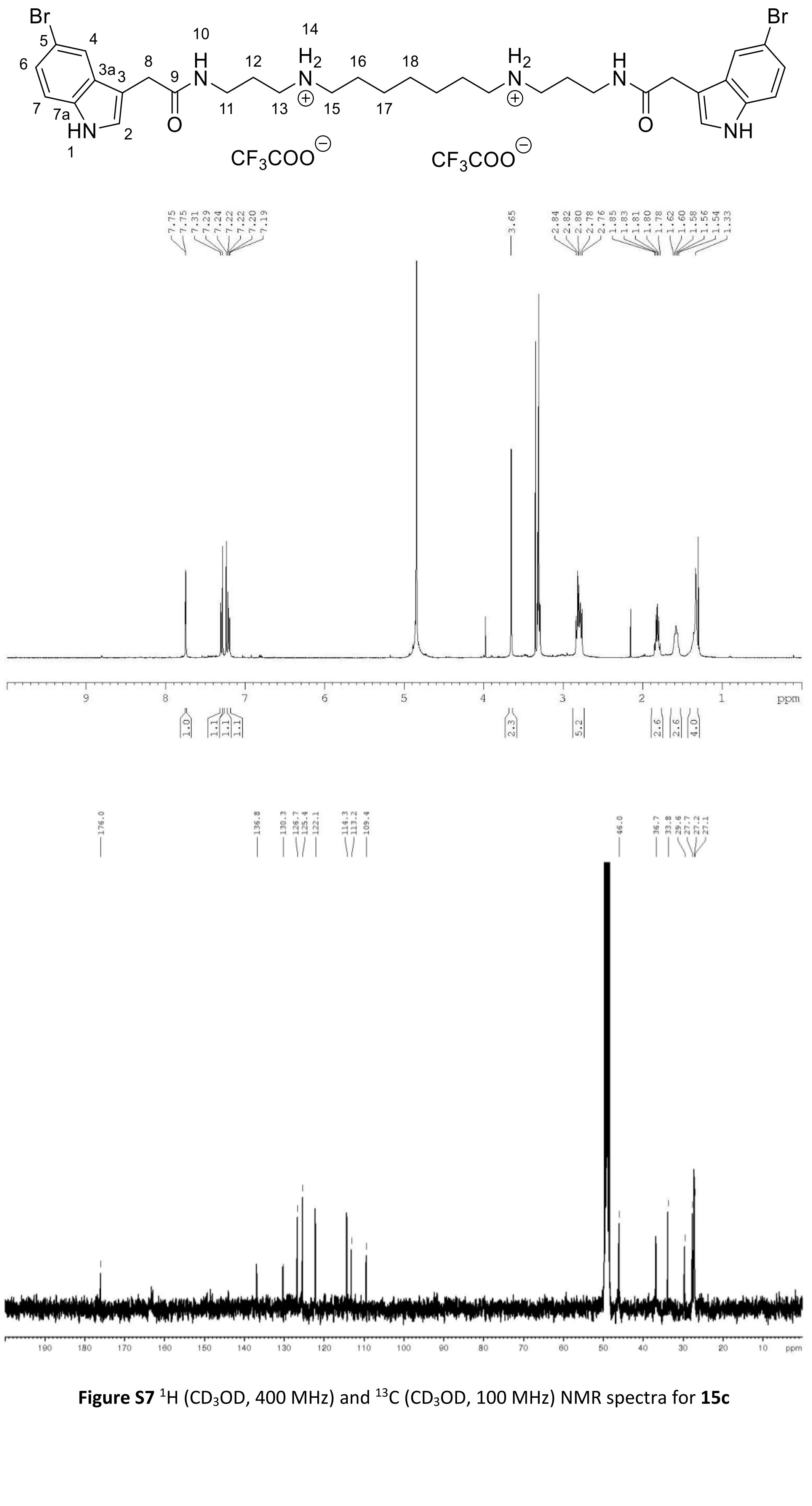

Figure S7 ^{1}H (CD_3OD, 400 MHz) and ^{13}C (CD_3OD, 100 MHz) NMR spectra for **15c**

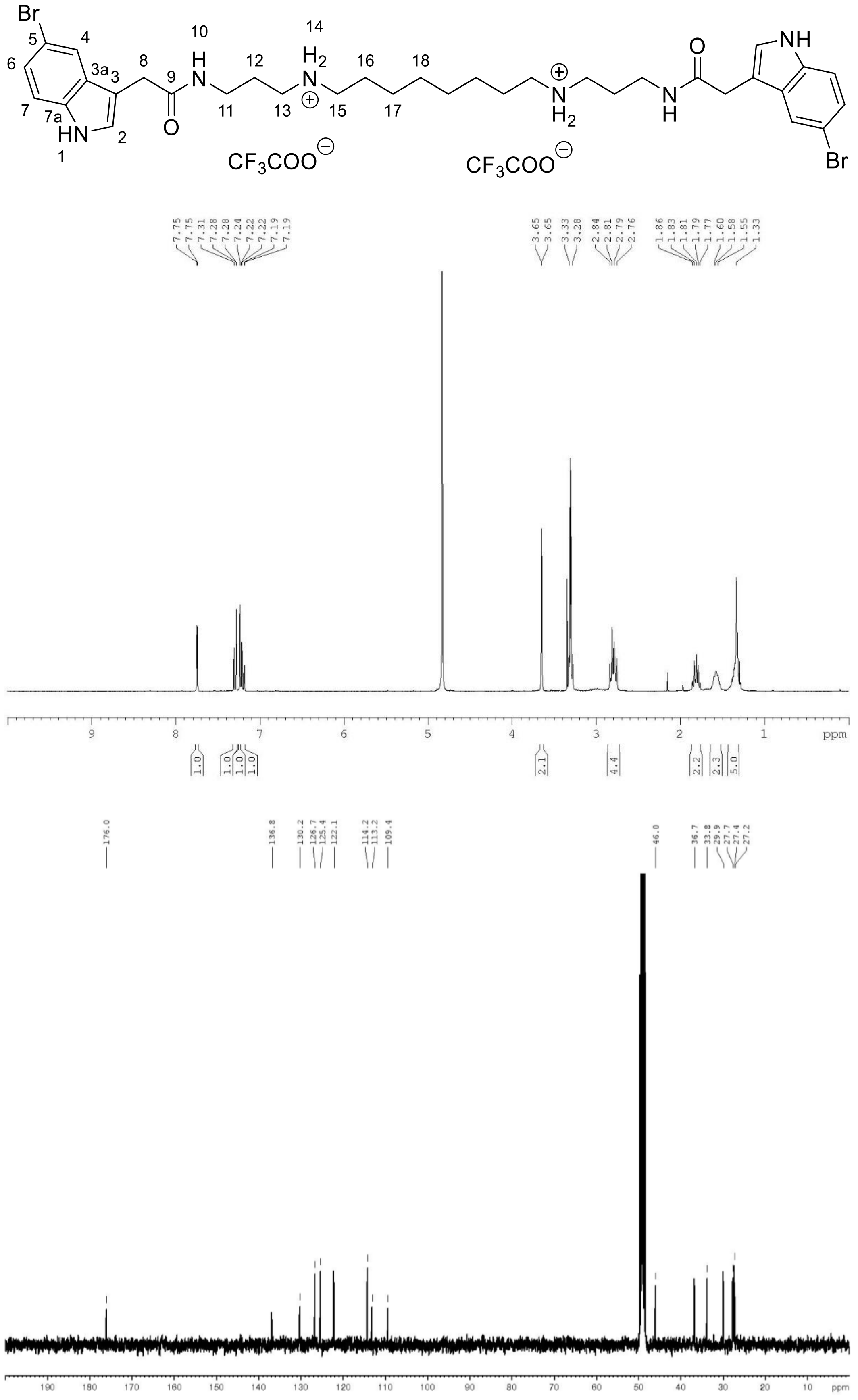

Figure S8 ${}^{1}H$ (CD_3OD, 400 MHz) and ${}^{13}C$ (CD_3OD, 100 MHz) NMR spectra for **15d**

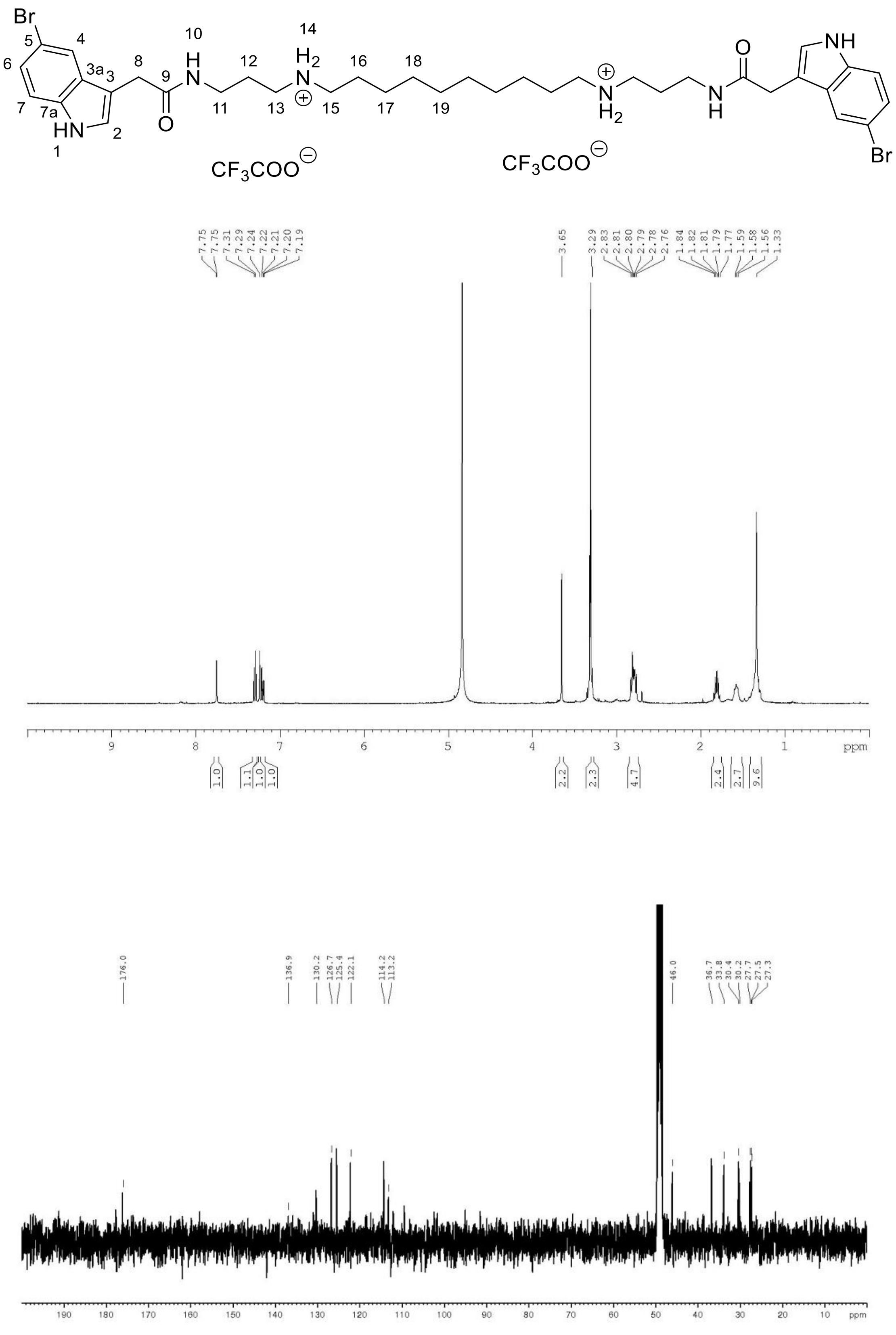

Figure S9 ^{1}H (CD_3OD, 400 MHz) and ^{13}C (CD_3OD, 100 MHz) NMR spectra for **15e**

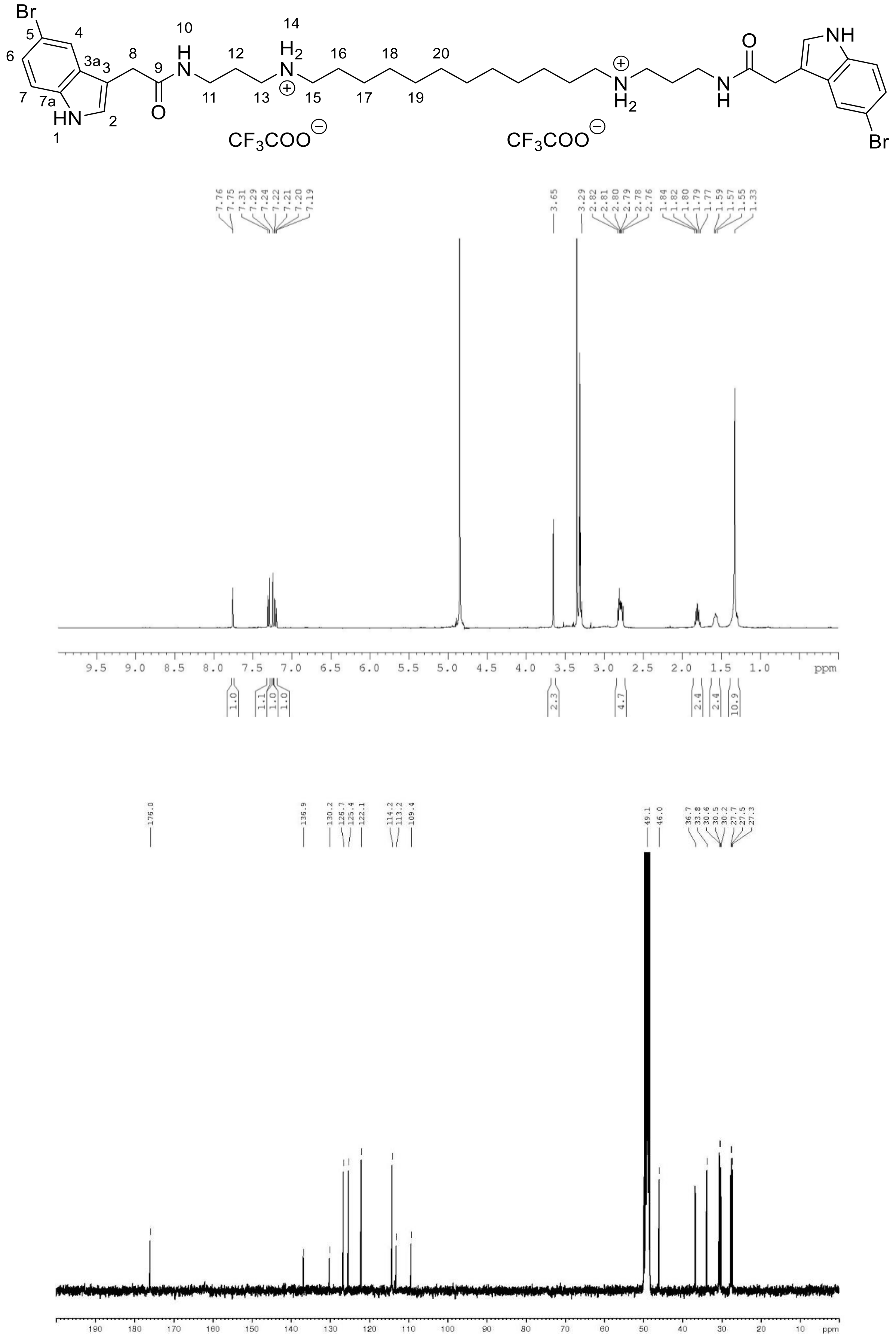

Figure S10 ^{1}H (CD_3OD, 400 MHz) and ^{13}C (CD_3OD, 100 MHz) NMR spectra for **15f**

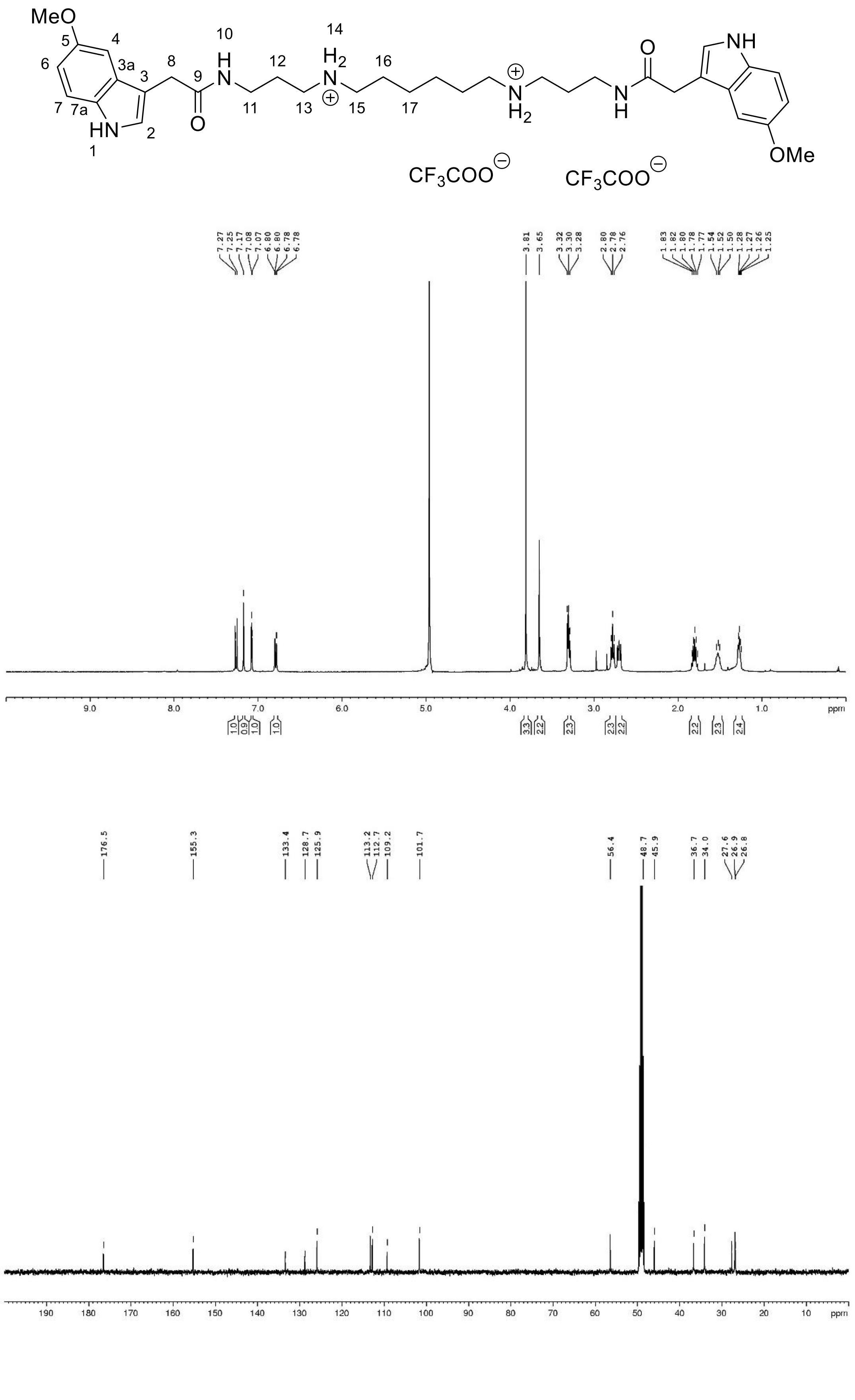

Figure S11 ^{1}H (CD_3OD, 400 MHz) and ^{13}C (CD_3OD, 100 MHz) NMR spectra for **16b**

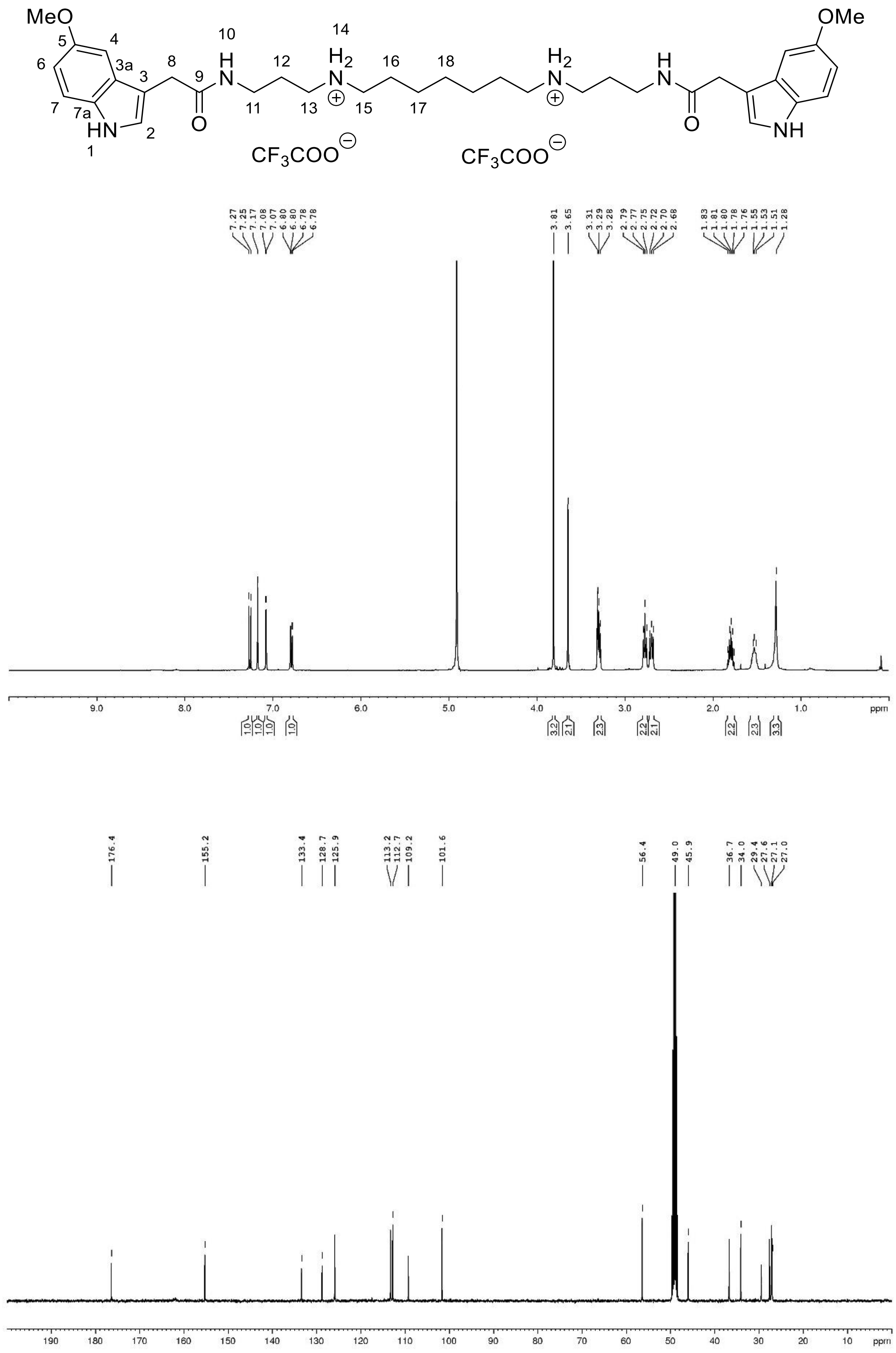

Figure S12 ^{1}H (CD_3OD, 400 MHz) and ^{13}C (CD_3OD, 100 MHz) NMR spectra for **16c**

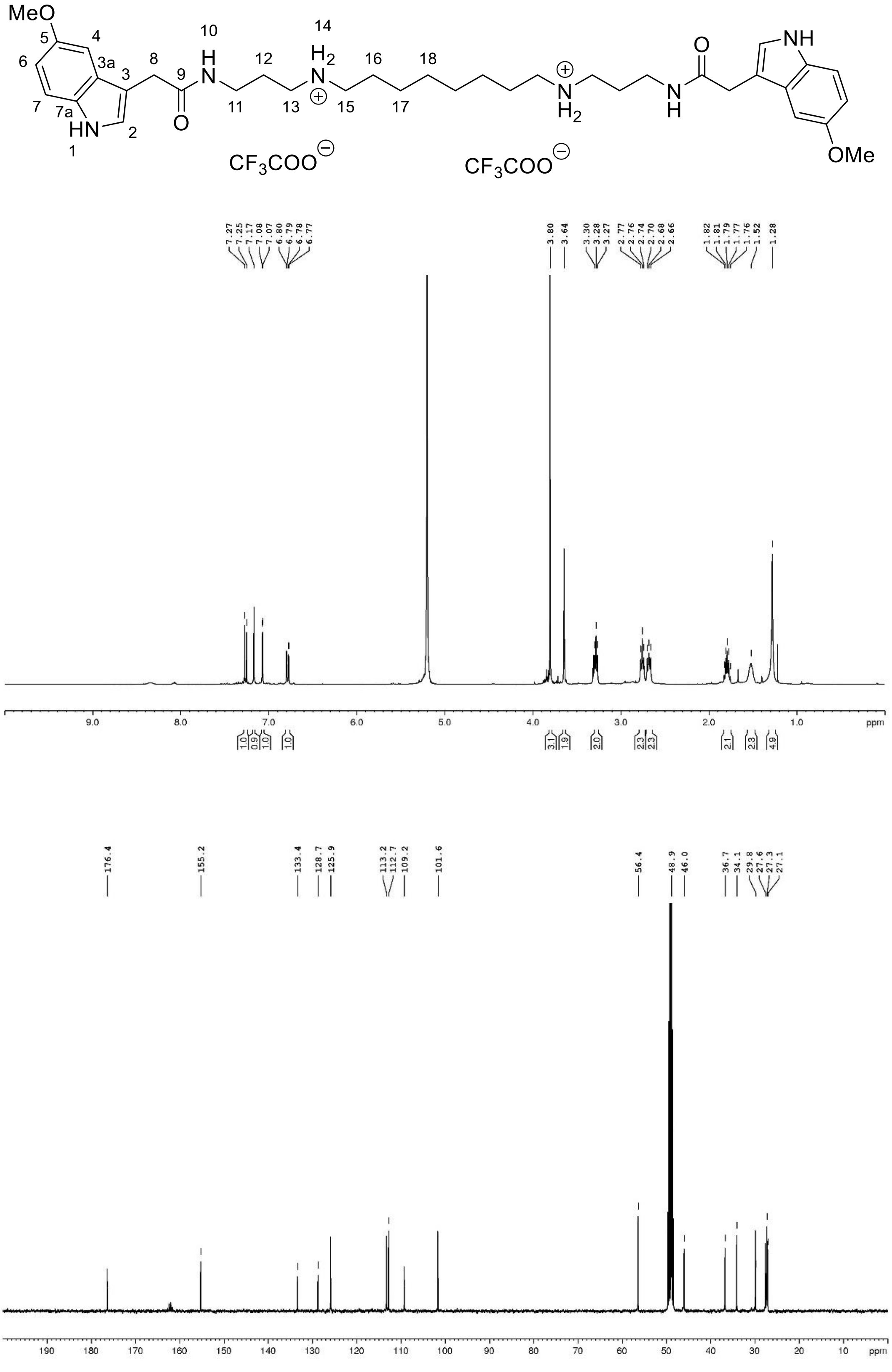

Figure S13 ^{1}H (CD_3OD, 400 MHz) and ^{13}C (CD_3OD, 100 MHz) NMR spectra for **16d**

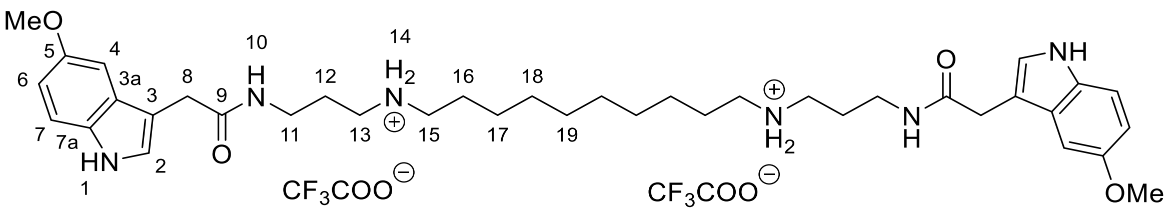

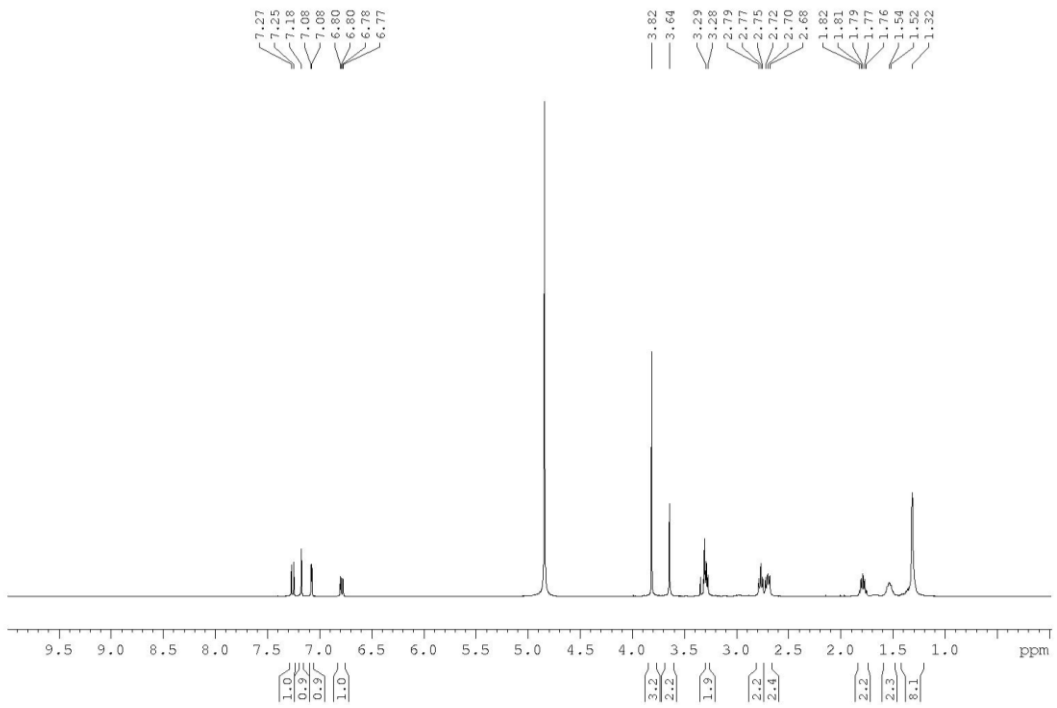

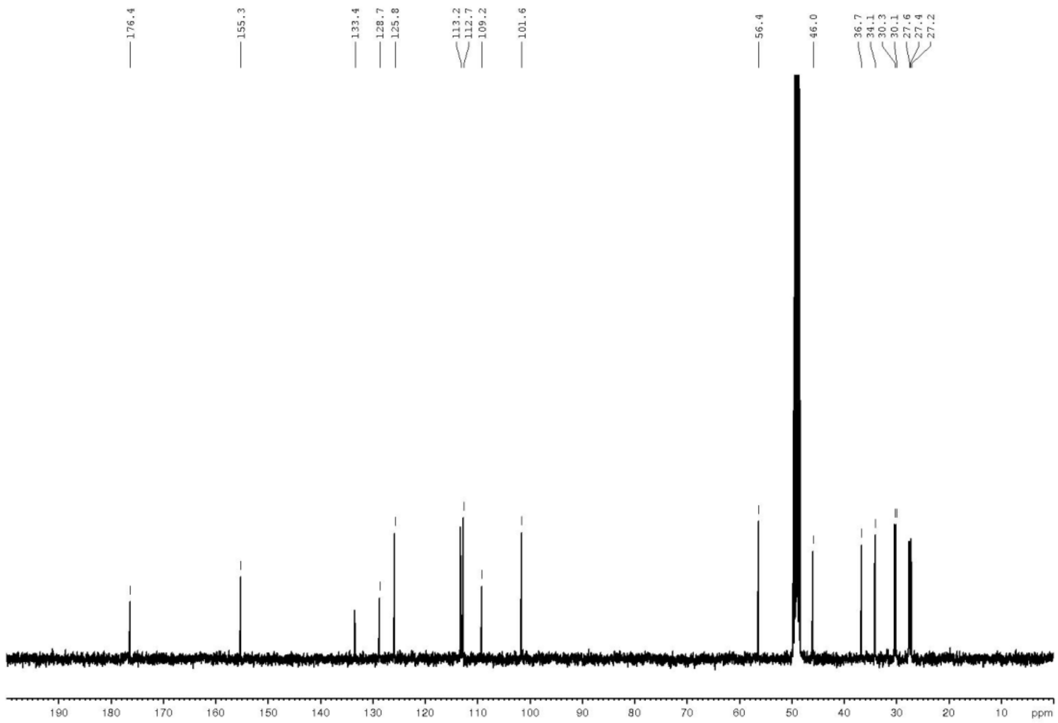

Figure S14 ¹H (CD_3OD, 400 MHz) and ¹³C (CD_3OD, 100 MHz) NMR spectra for **16e**

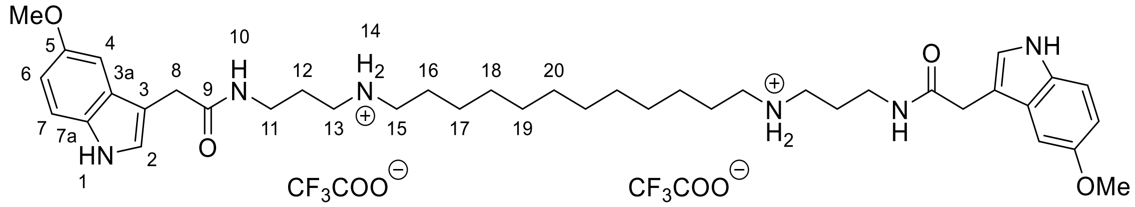

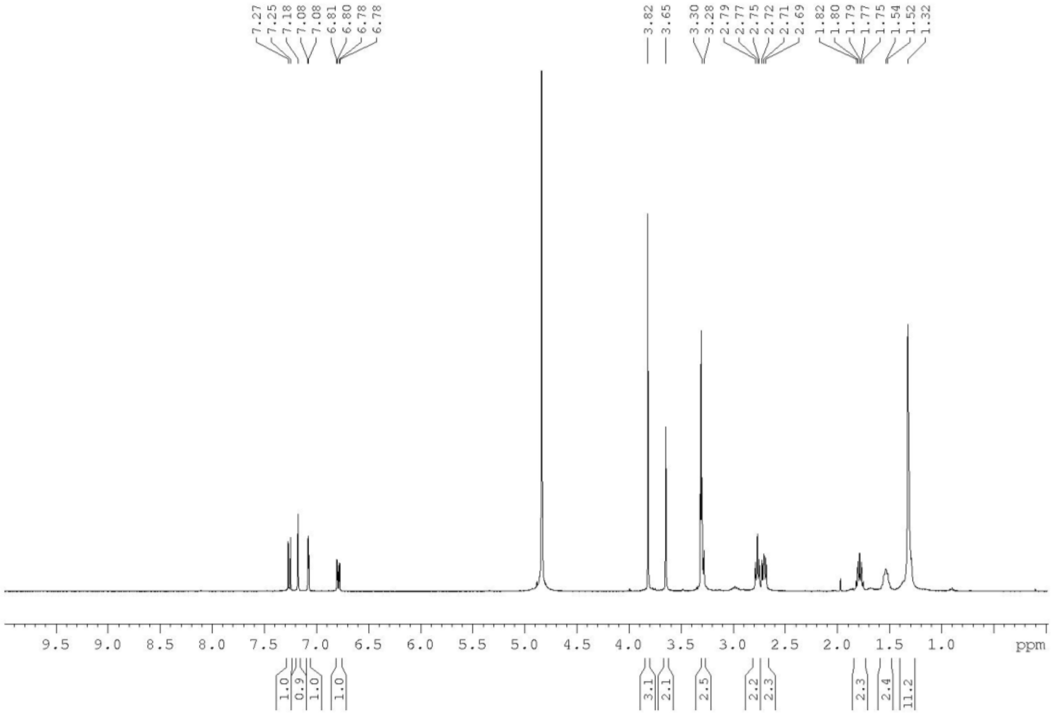

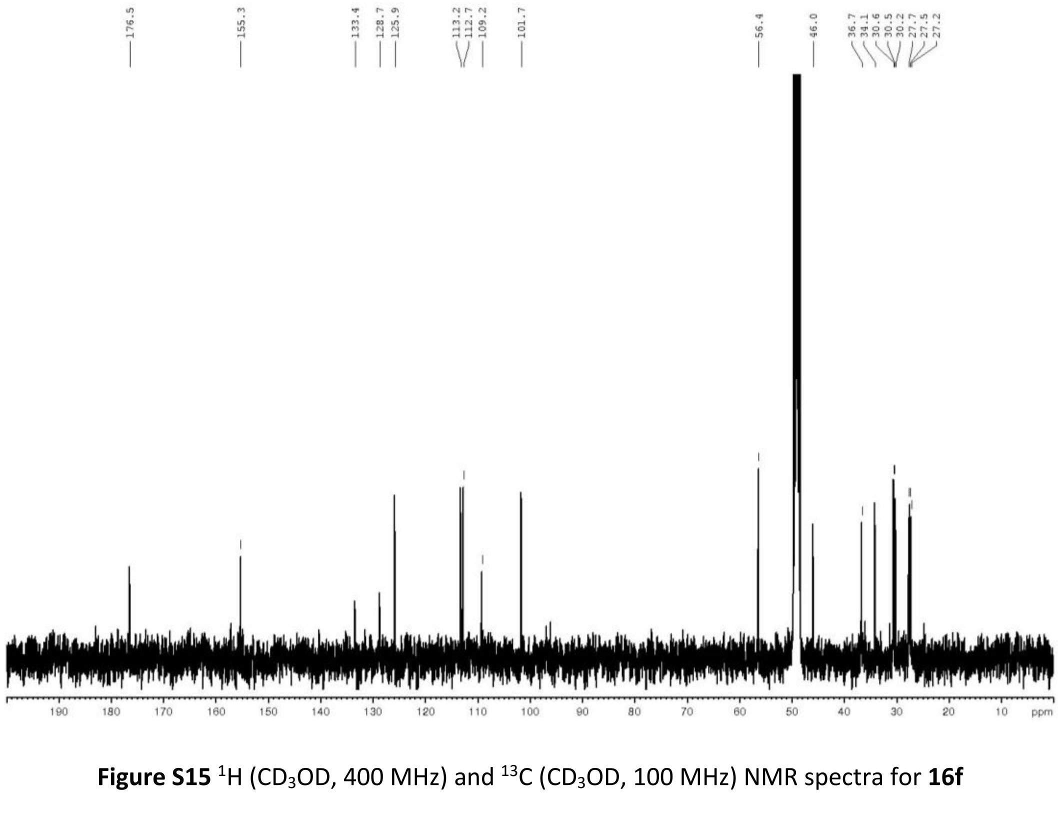

Figure S15 ^{1}H (CD_3OD, 400 MHz) and ^{13}C (CD_3OD, 100 MHz) NMR spectra for **16f**

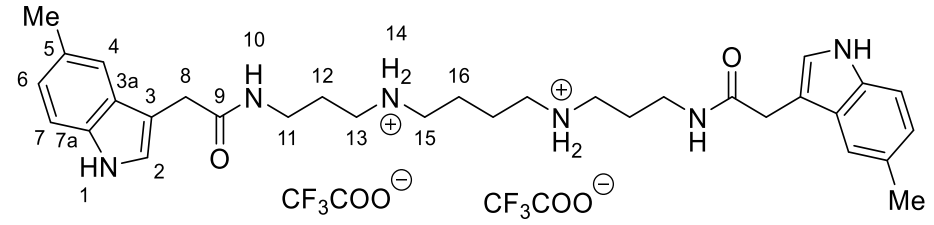

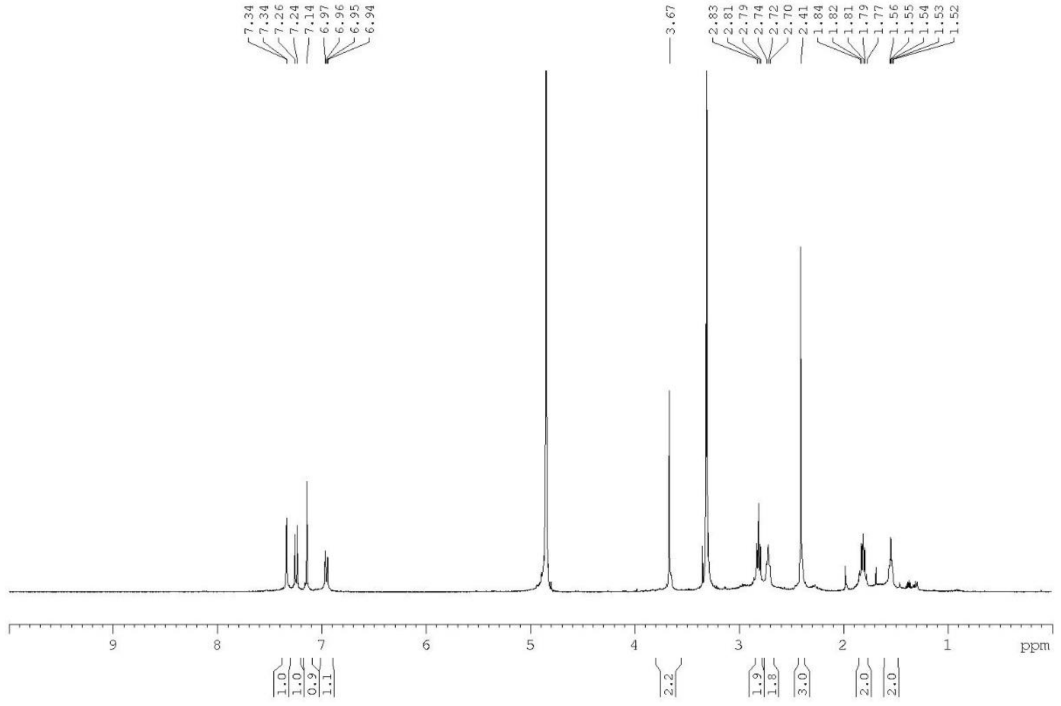

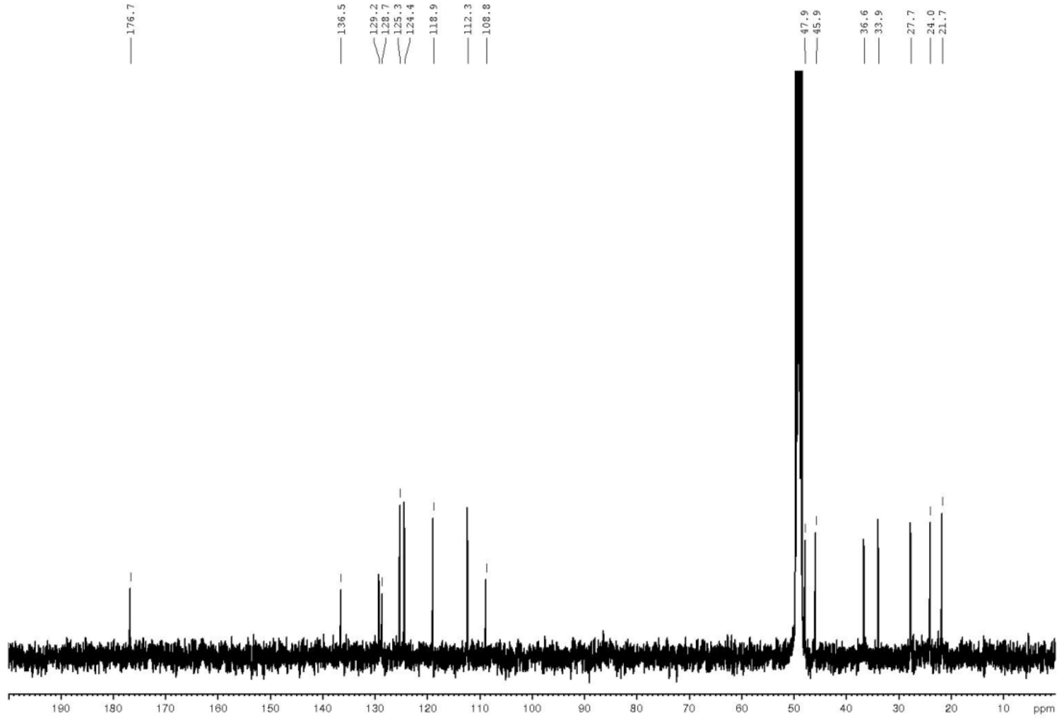

Figure S16 ^{1}H (CD_3OD, 400 MHz) and ^{13}C (CD_3OD, 100 MHz) NMR spectra for **17a**

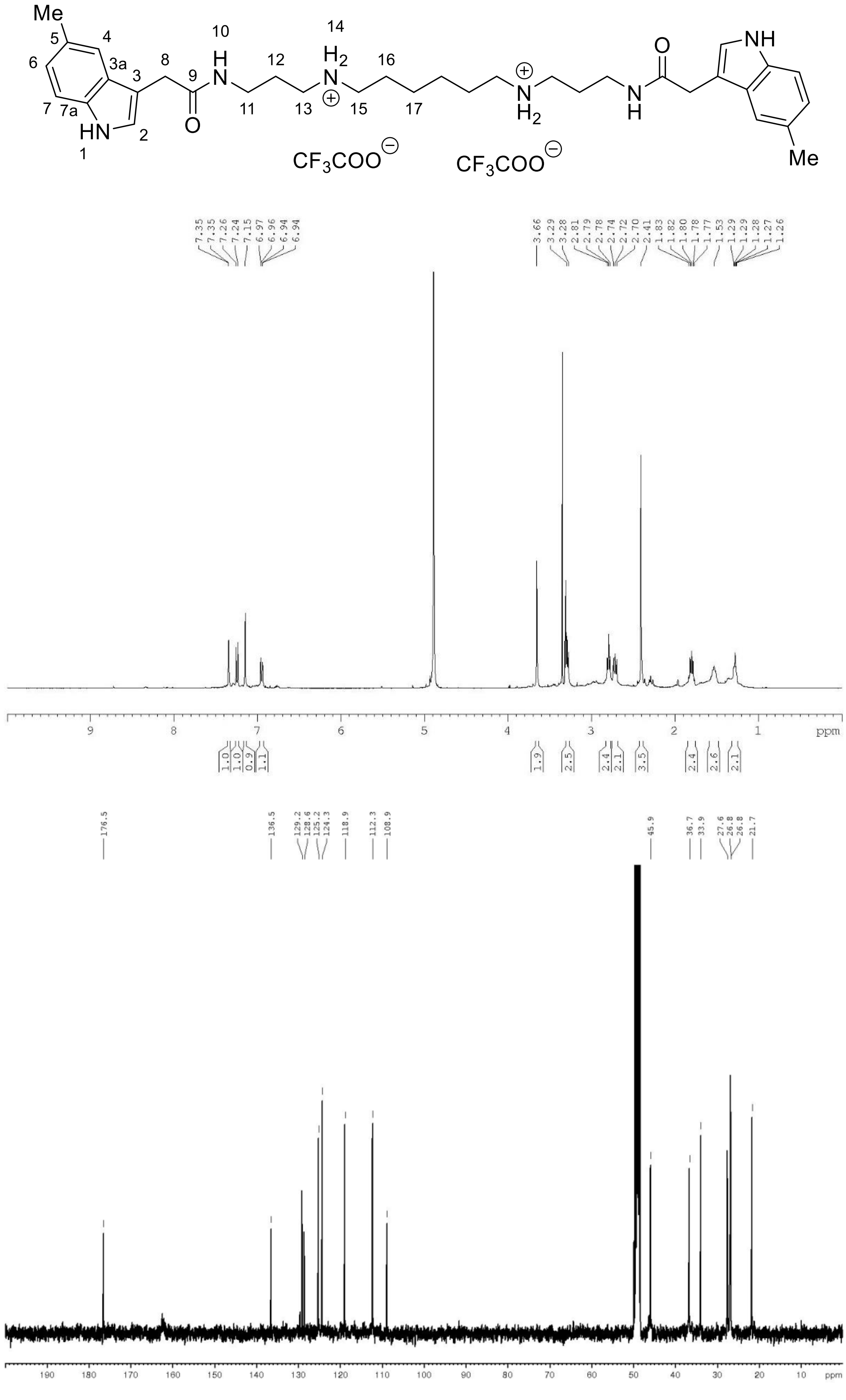

Figure S17 ^{1}H (CD_3OD, 400 MHz) and ^{13}C (CD_3OD, 100 MHz) NMR spectra for **17b**

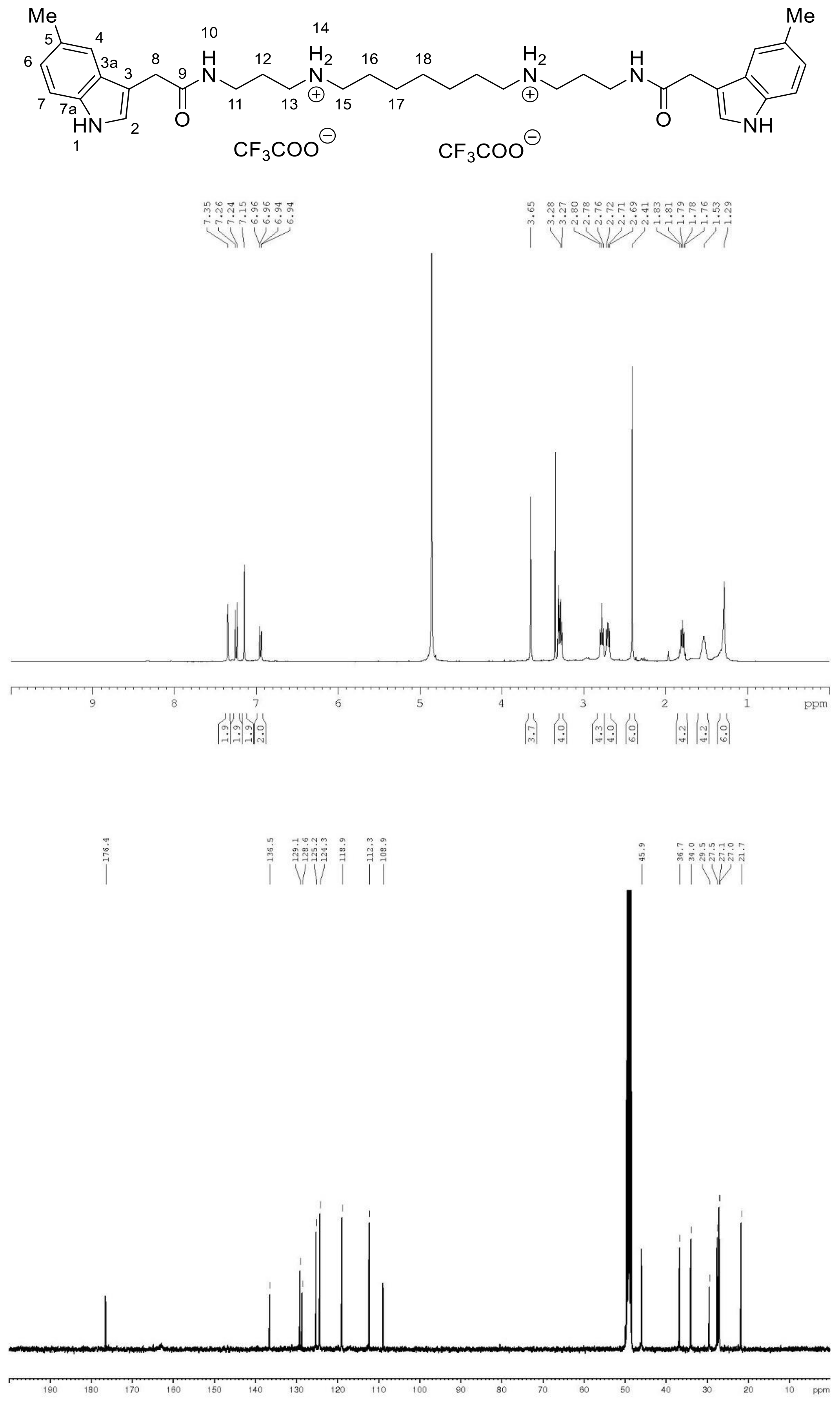

Figure S18 ^{1}H (CD_3OD, 400 MHz) and ^{13}C (CD_3OD, 100 MHz) NMR spectra for **17c**

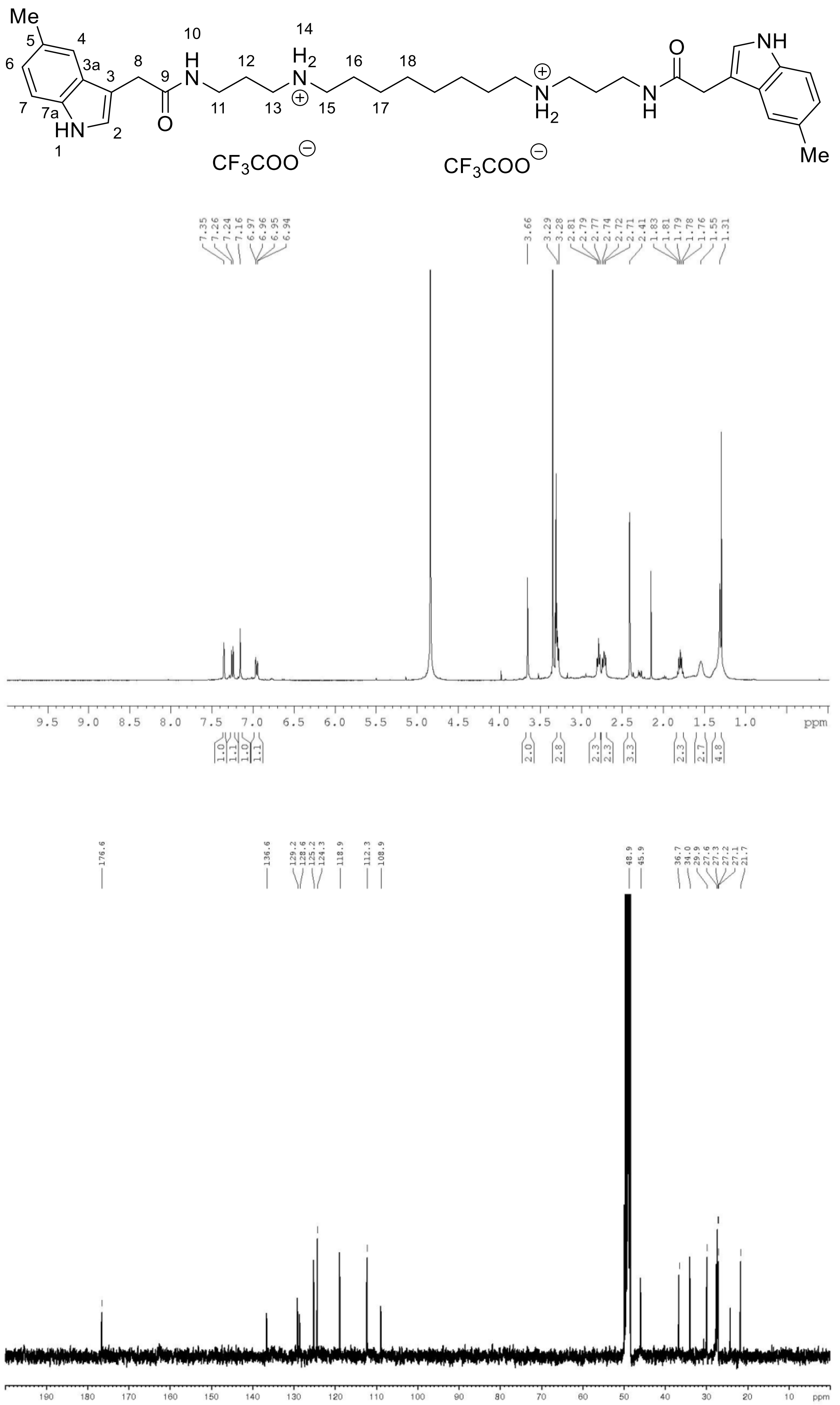

Figure S19 ^{1}H (CD_3OD, 400 MHz) and ^{13}C (CD_3OD, 100 MHz) NMR spectra for **17d**

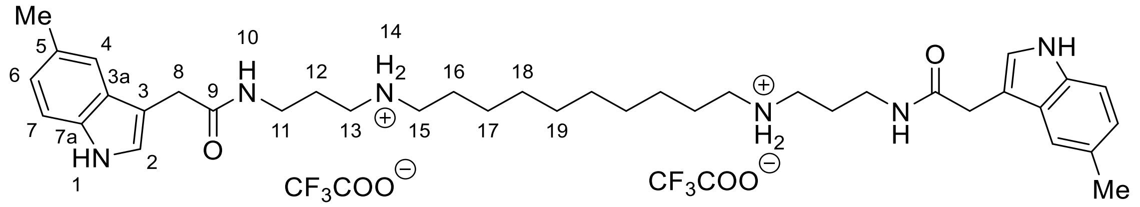

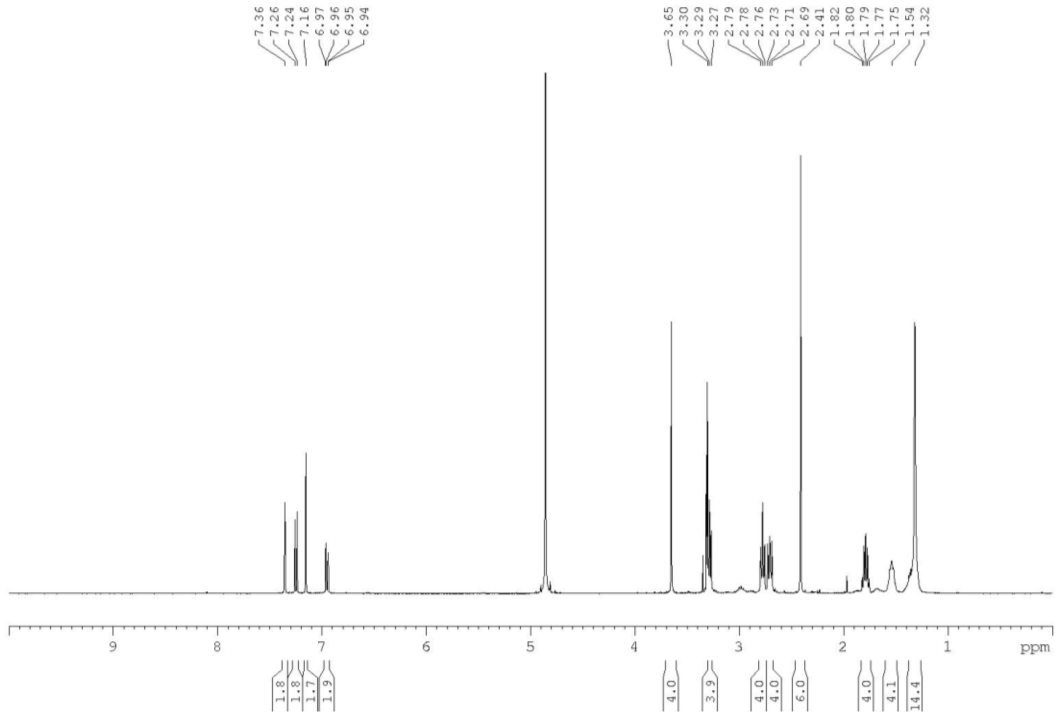

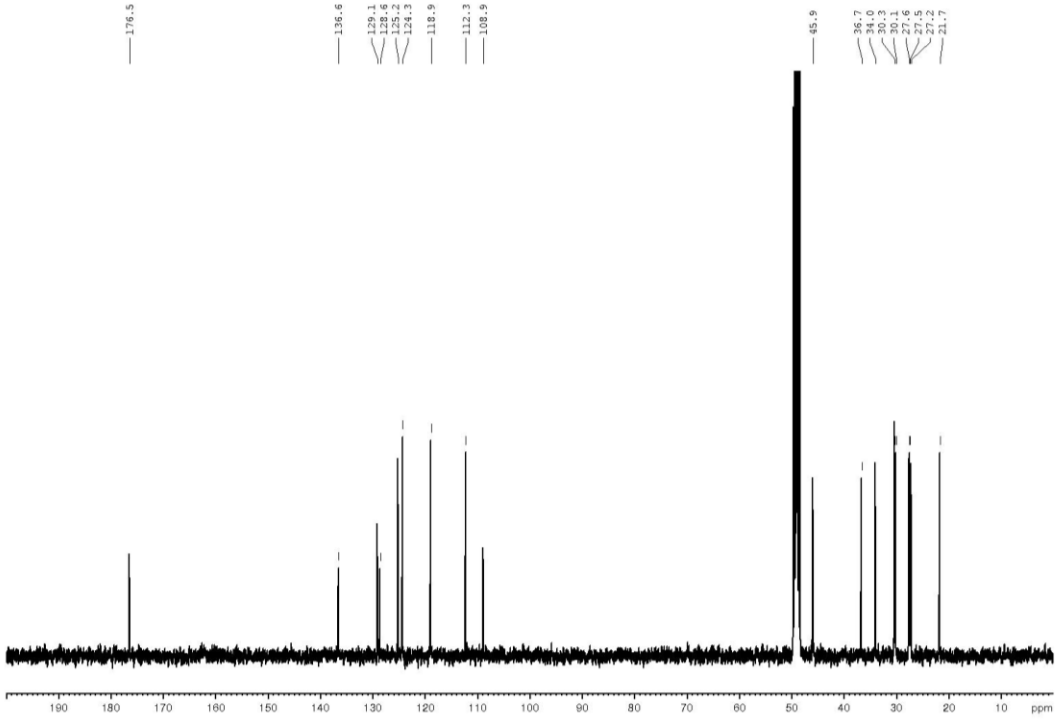

Figure S20 ^{1}H (CD_3OD, 400 MHz) and ^{13}C (CD_3OD, 100 MHz) NMR spectra for **17e**

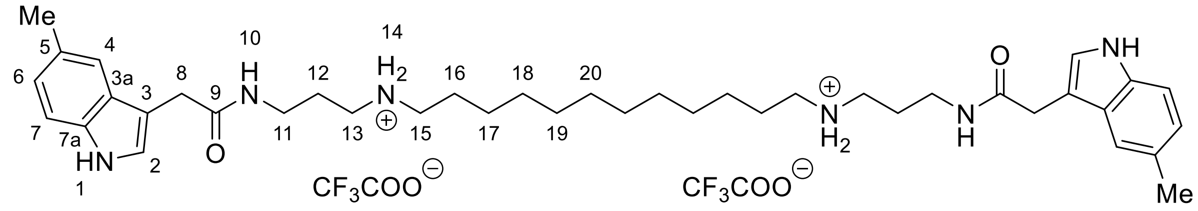

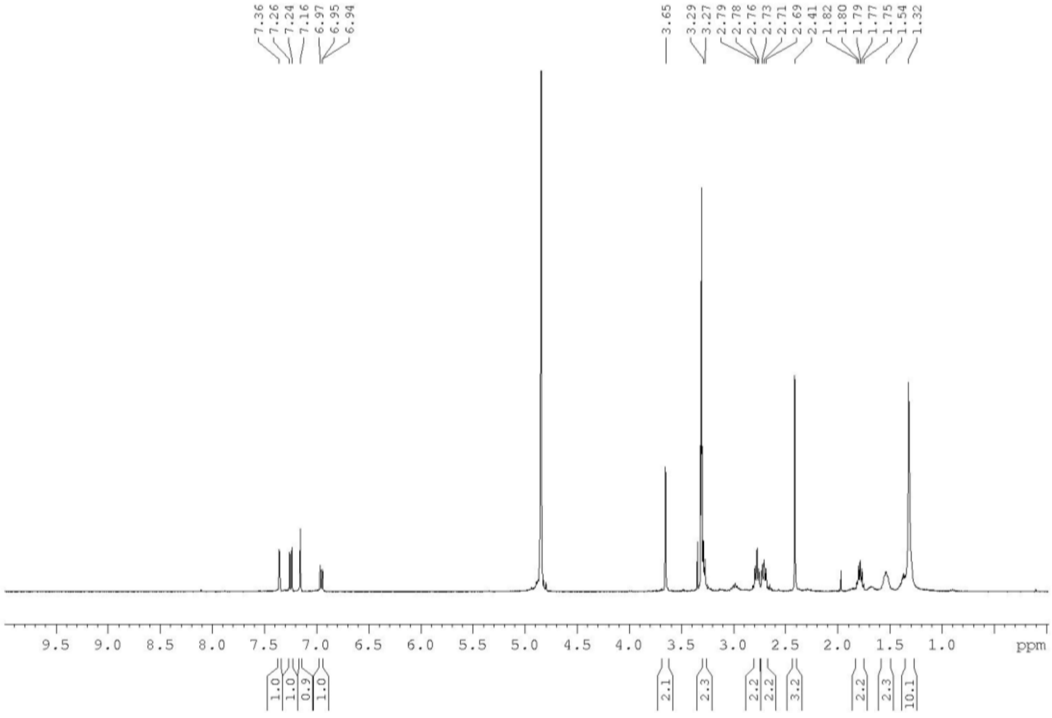

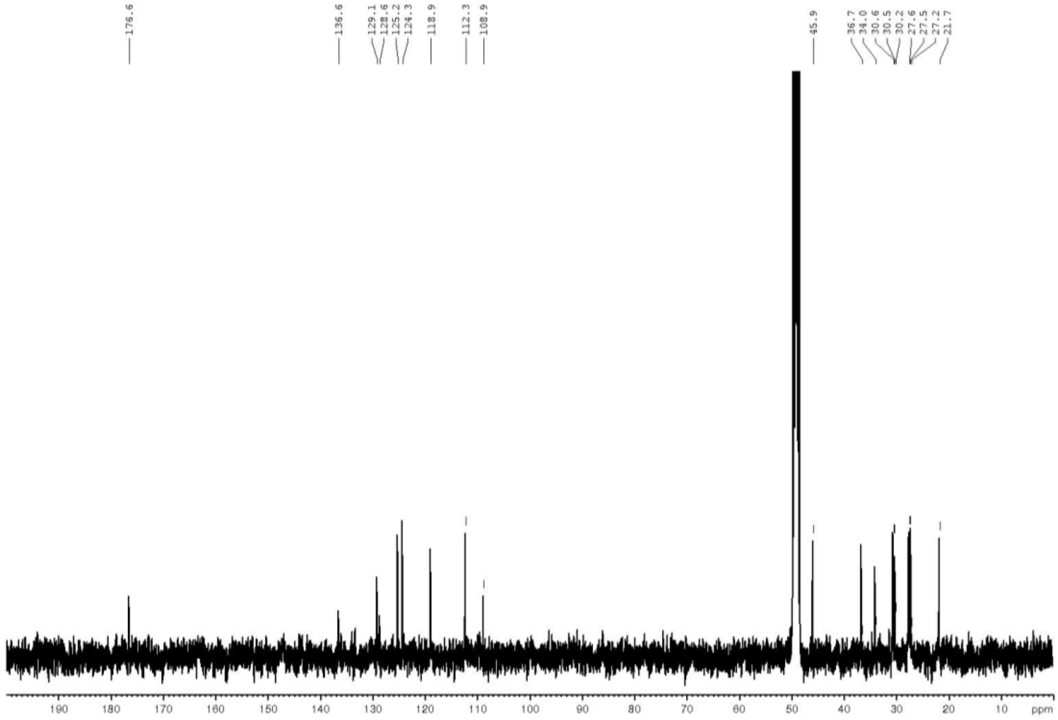

Figure S21 ^{1}H (CD_3OD, 400 MHz) and ^{13}C (CD_3OD, 100 MHz) NMR spectra for **17f**

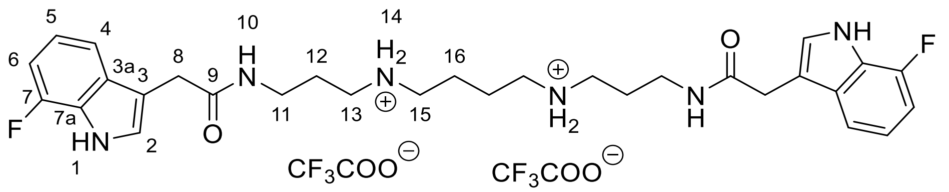

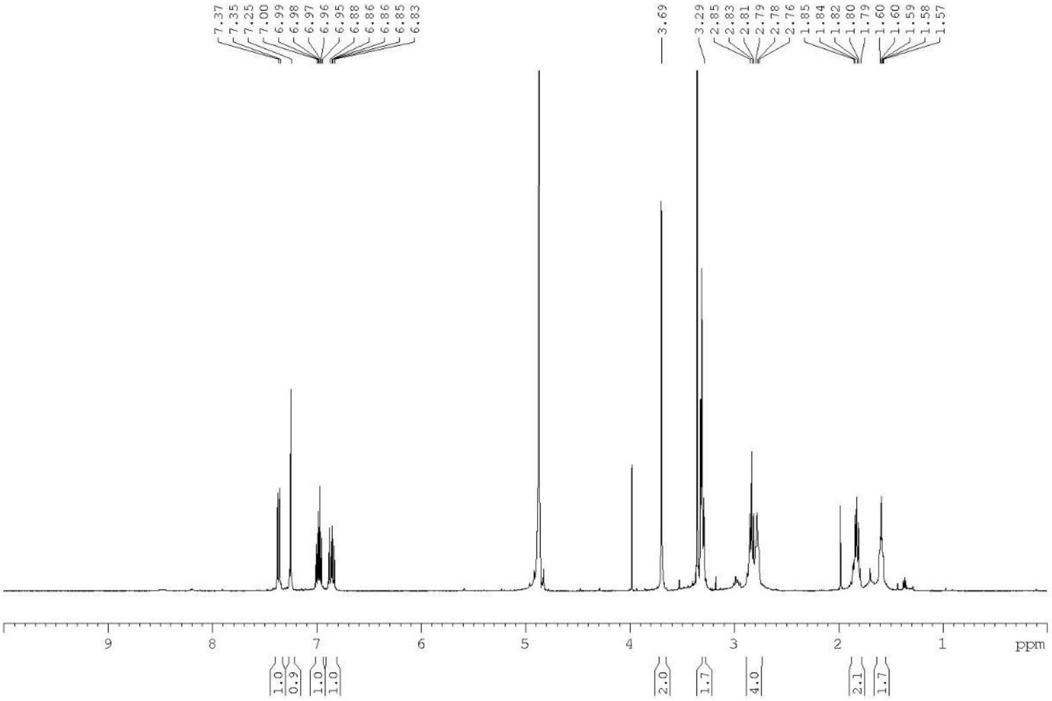

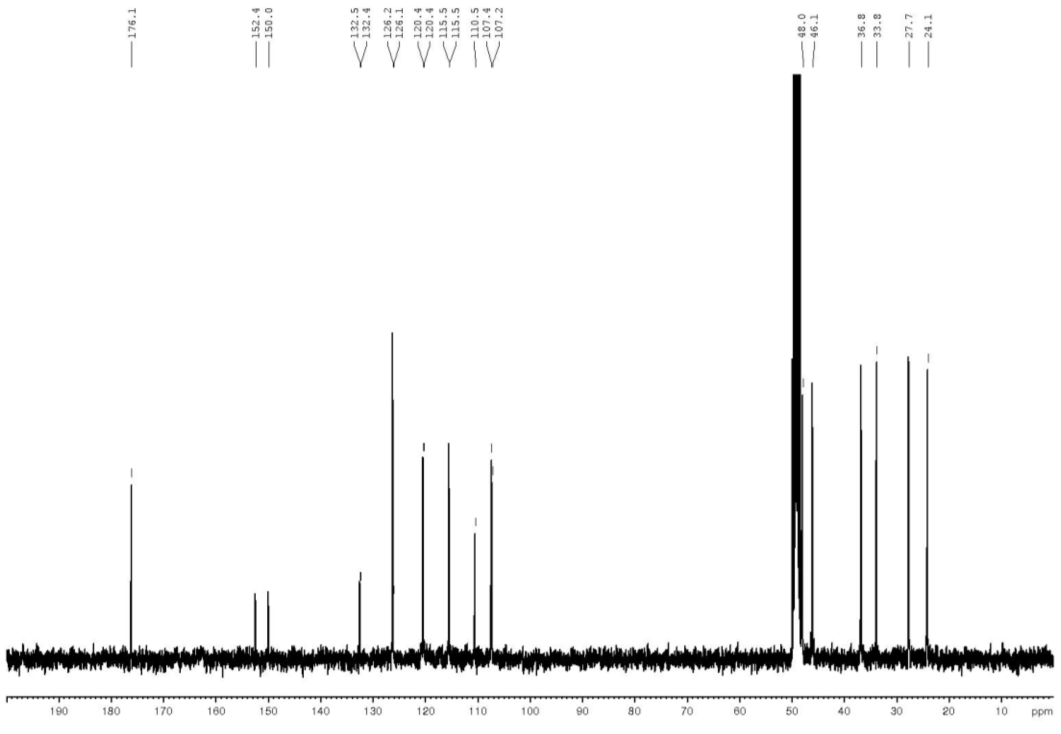

Figure S22 ^{1}H (CD_3OD, 400 MHz) and ^{13}C (CD_3OD, 100 MHz) NMR spectra for **18a**

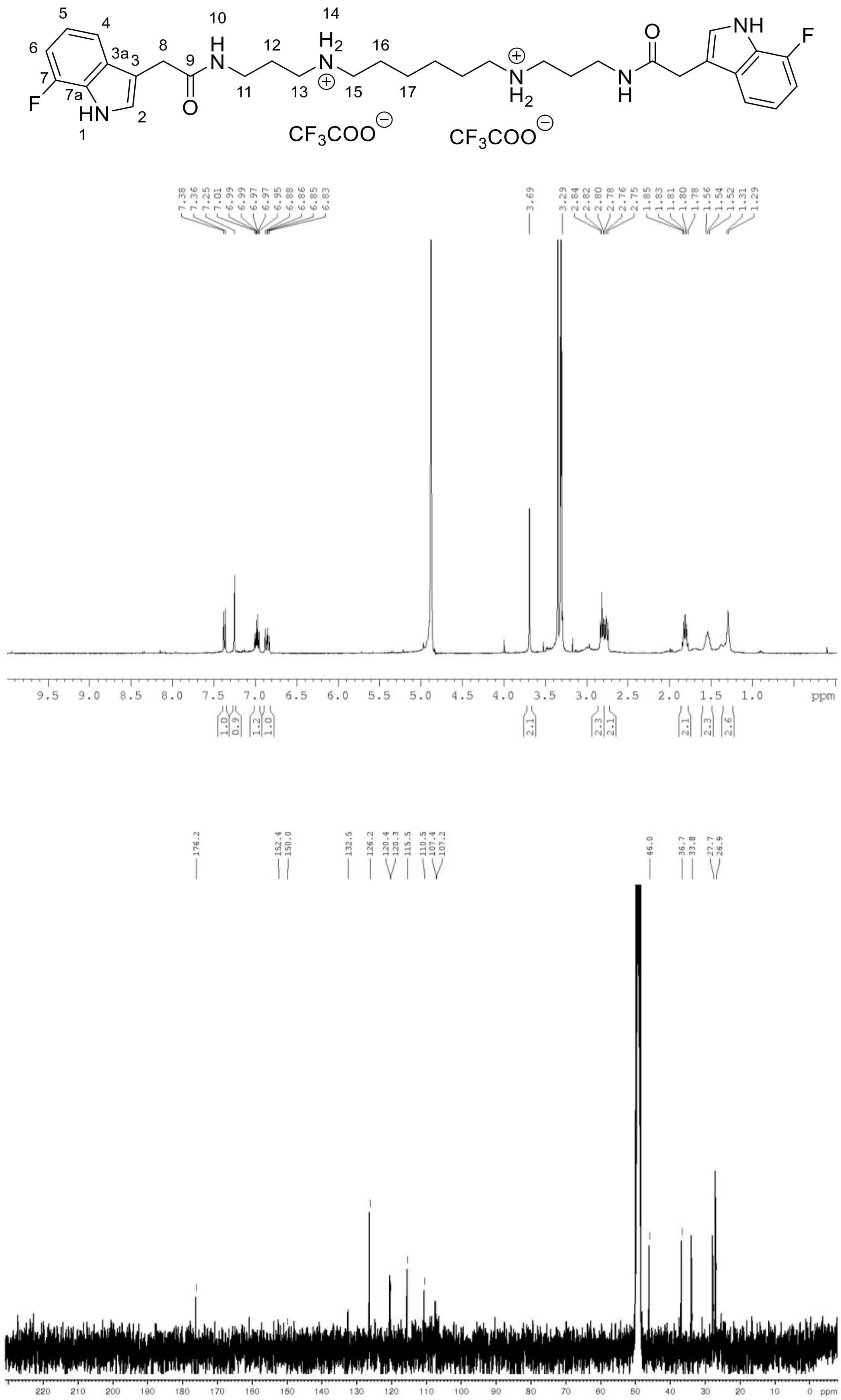

Figure S23 ^{1}H (CD_3OD, 400 MHz) and ^{13}C (CD_3OD, 100 MHz) NMR spectra for **18b**

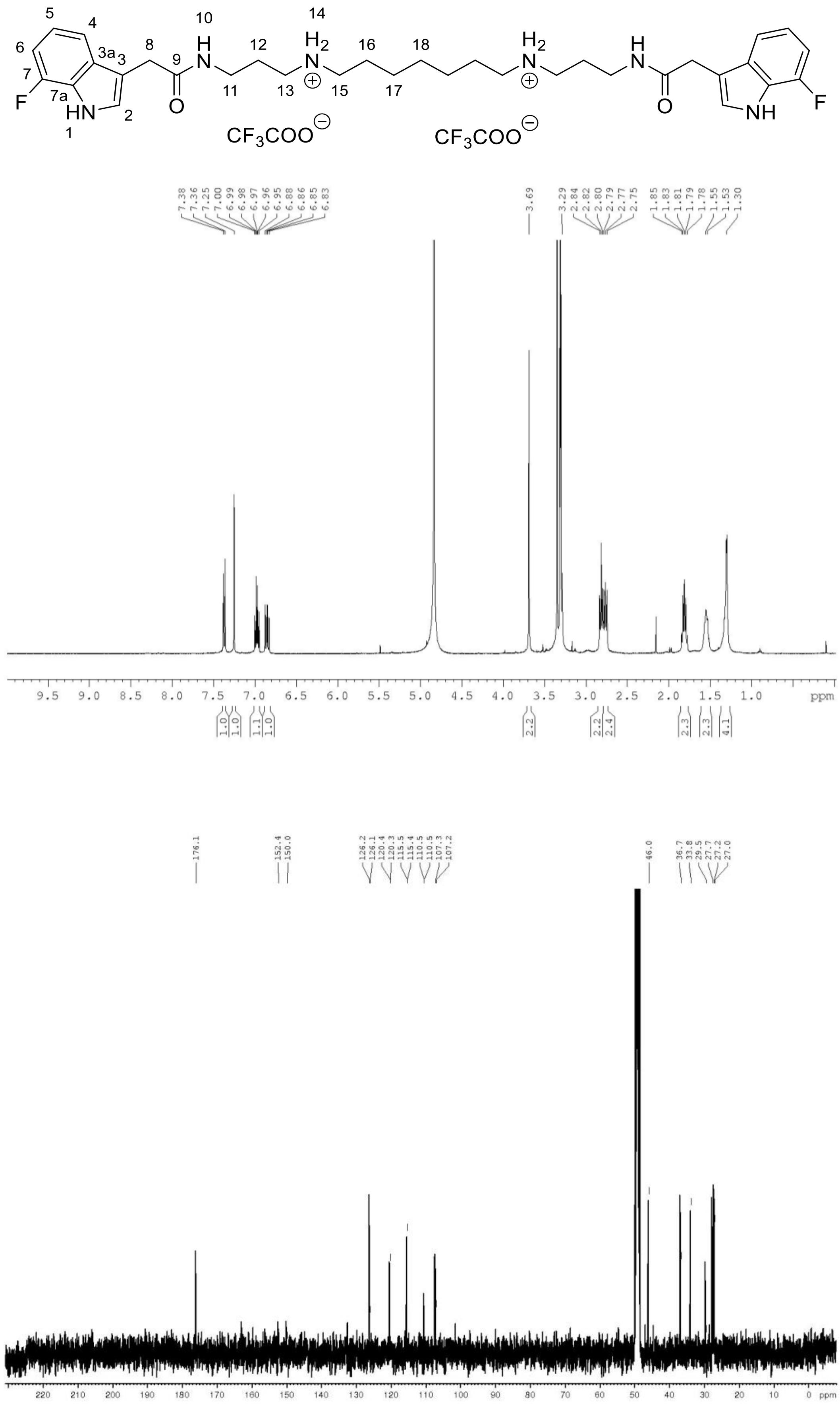

Figure S24 ^{1}H (CD_3OD, 400 MHz) and ^{13}C (CD_3OD, 100 MHz) NMR spectra for **18c**

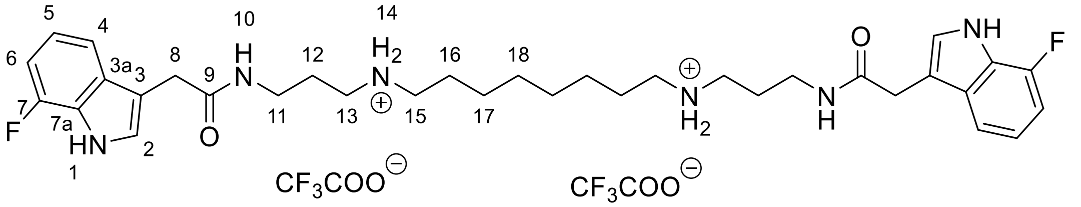

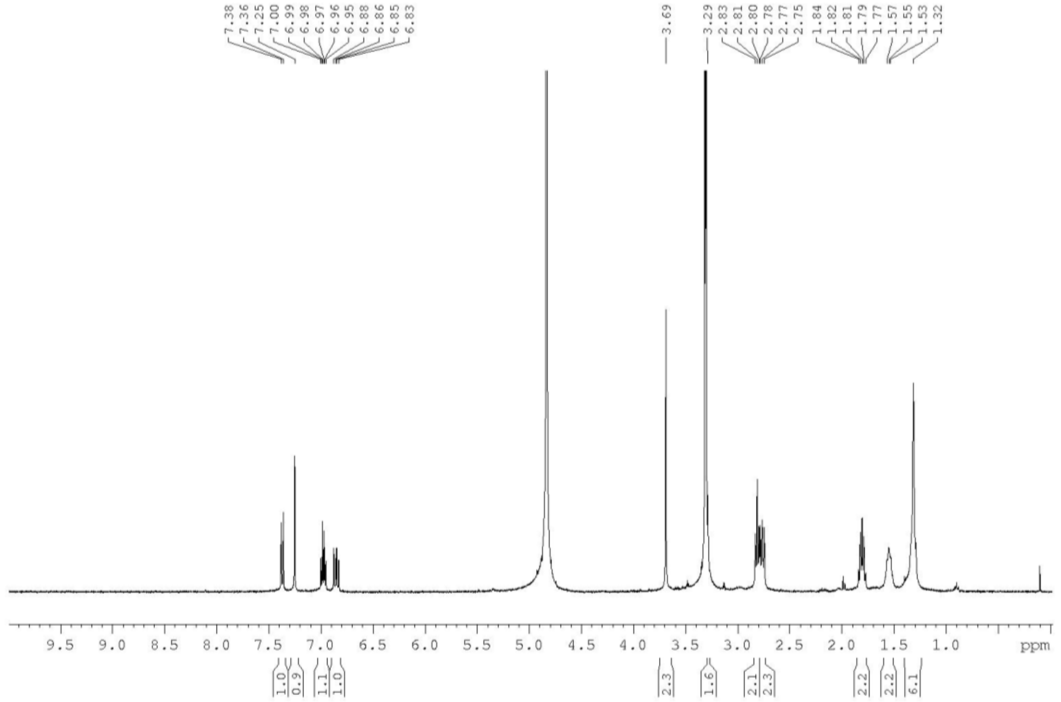

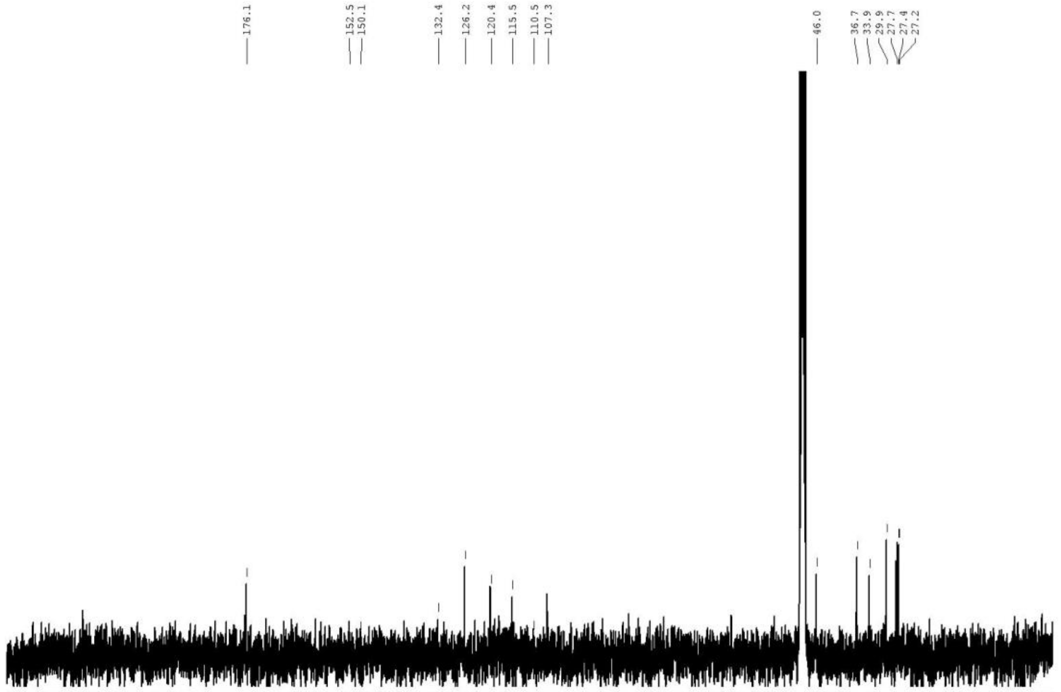

Figure S25 ^{1}H (CD_3OD, 400 MHz) and ^{13}C (CD_3OD, 100 MHz) NMR spectra for **18d**

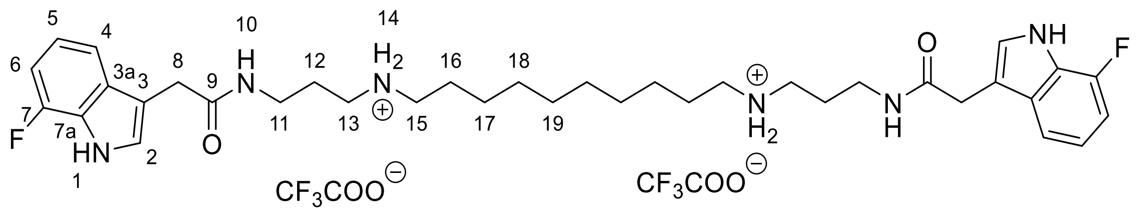

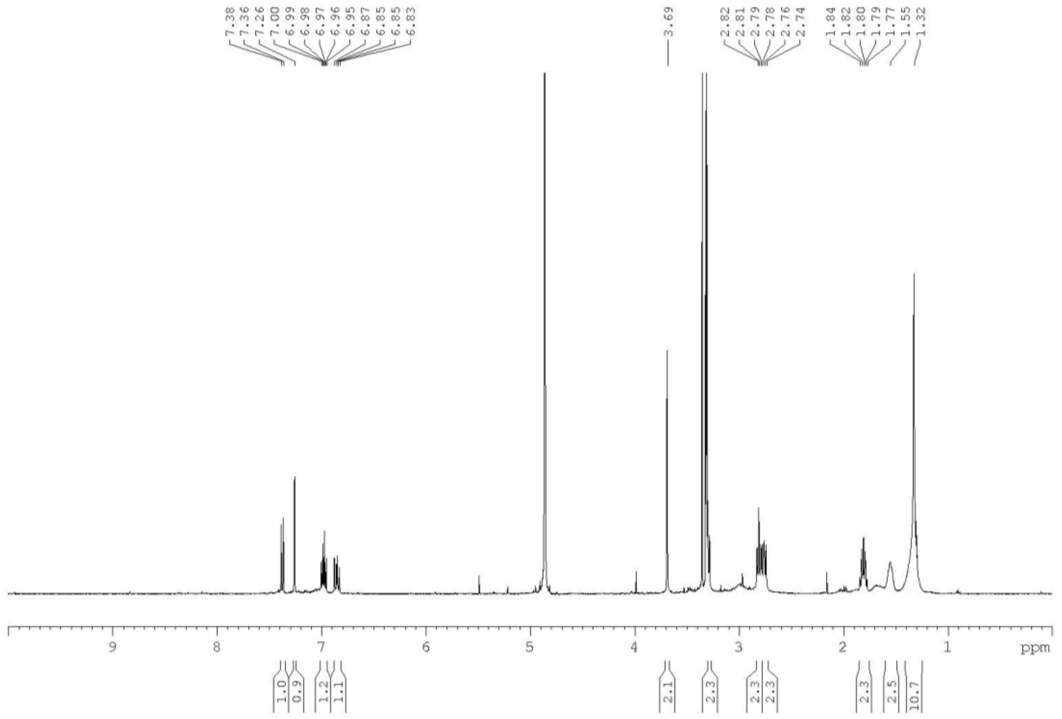

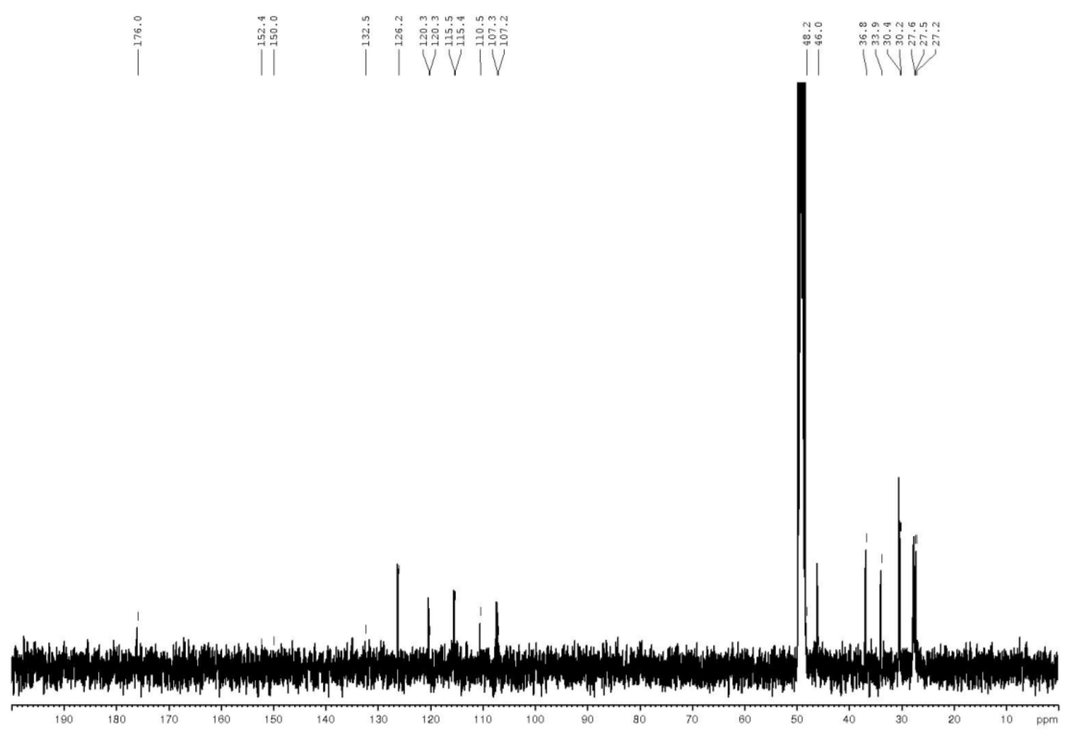

Figure S26 ^{1}H (CD_3OD, 400 MHz) and ^{13}C (CD_3OD, 100 MHz) NMR spectra for **18e**

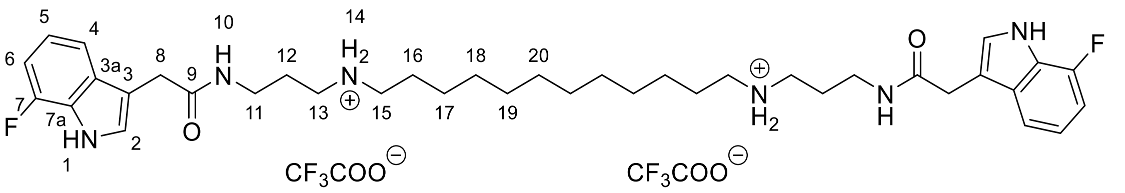

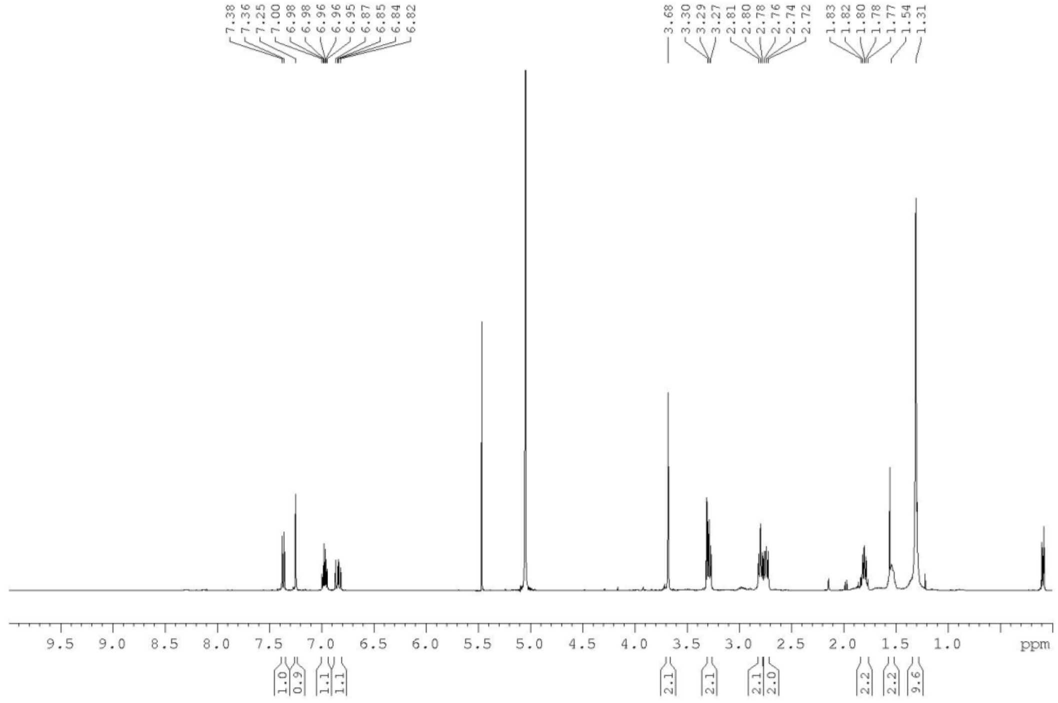

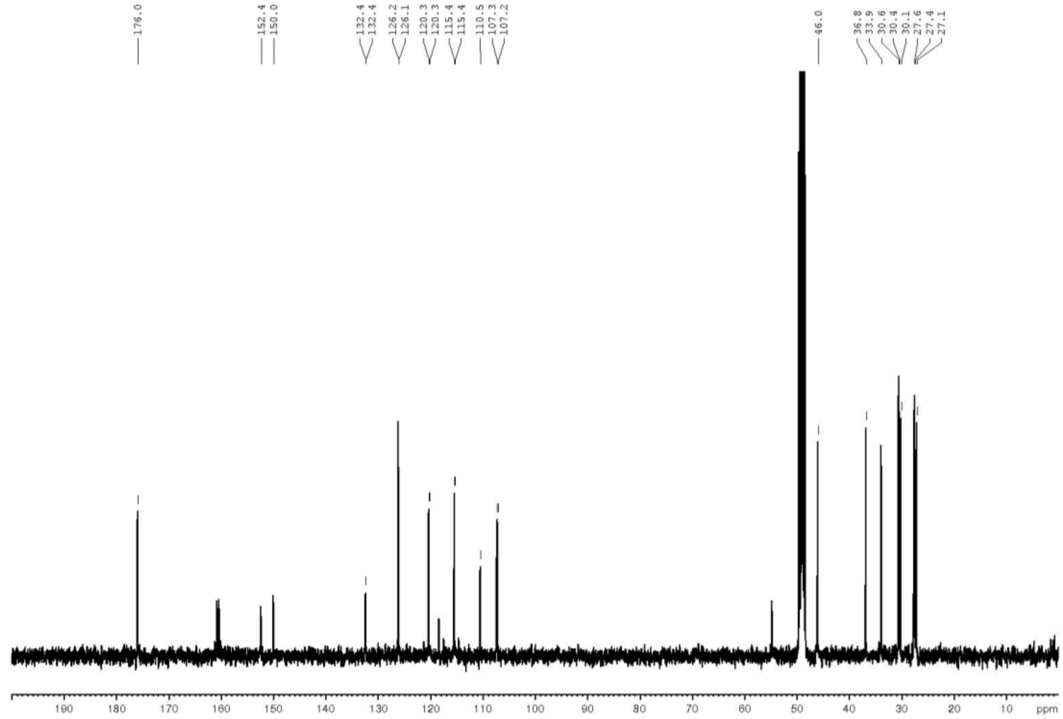

Figure S27 ^{1}H (CD_3OD, 400 MHz) and ^{13}C (CD_3OD, 100 MHz) NMR spectra for **18f**

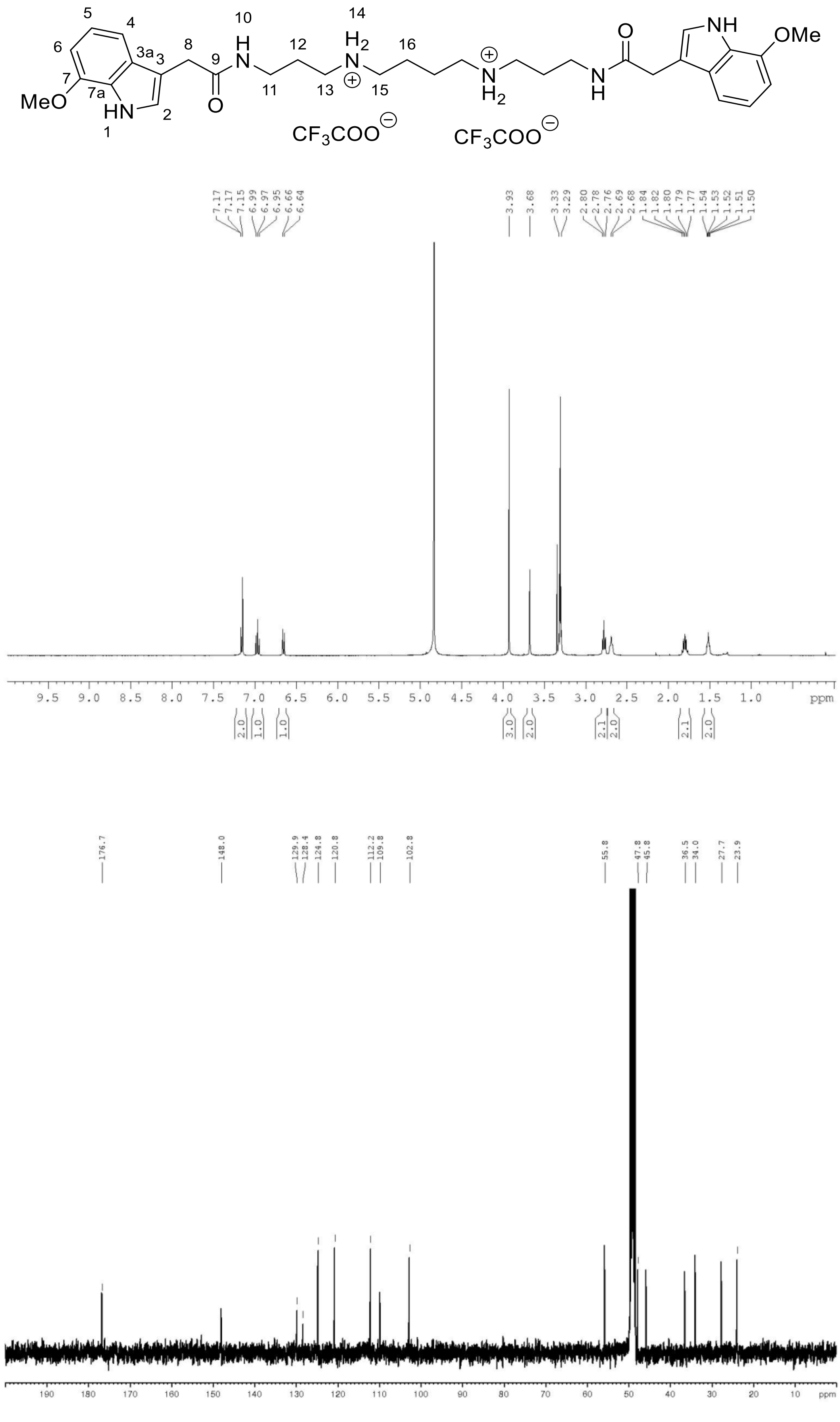

Figure S28 ^{1}H (CD_3OD, 400 MHz) and ^{13}C (CD_3OD, 100 MHz) NMR spectra for **19a**

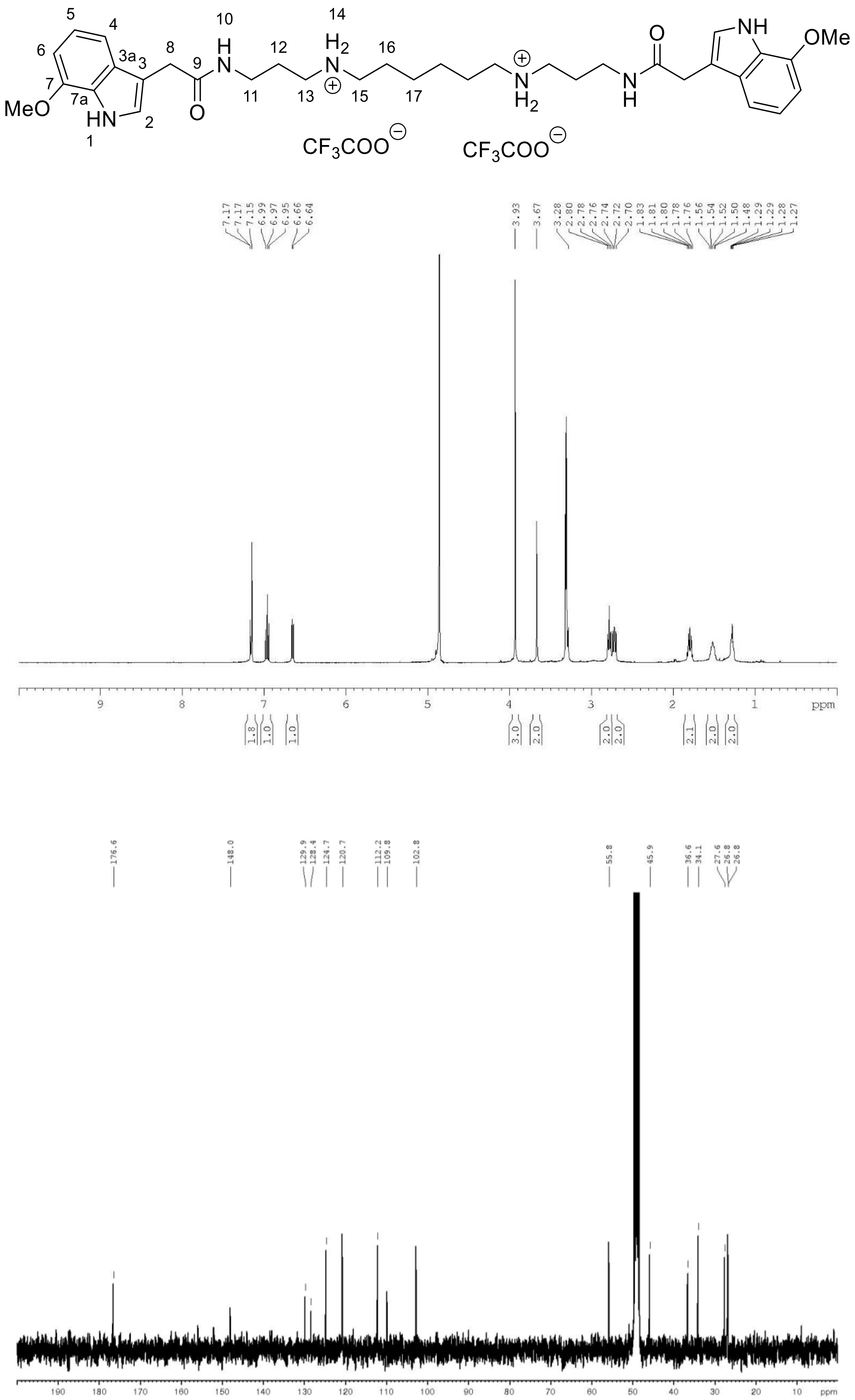

Figure S29 ^{1}H (CD_3OD, 400 MHz) and ^{13}C (CD_3OD, 100 MHz) NMR spectra for **19b**

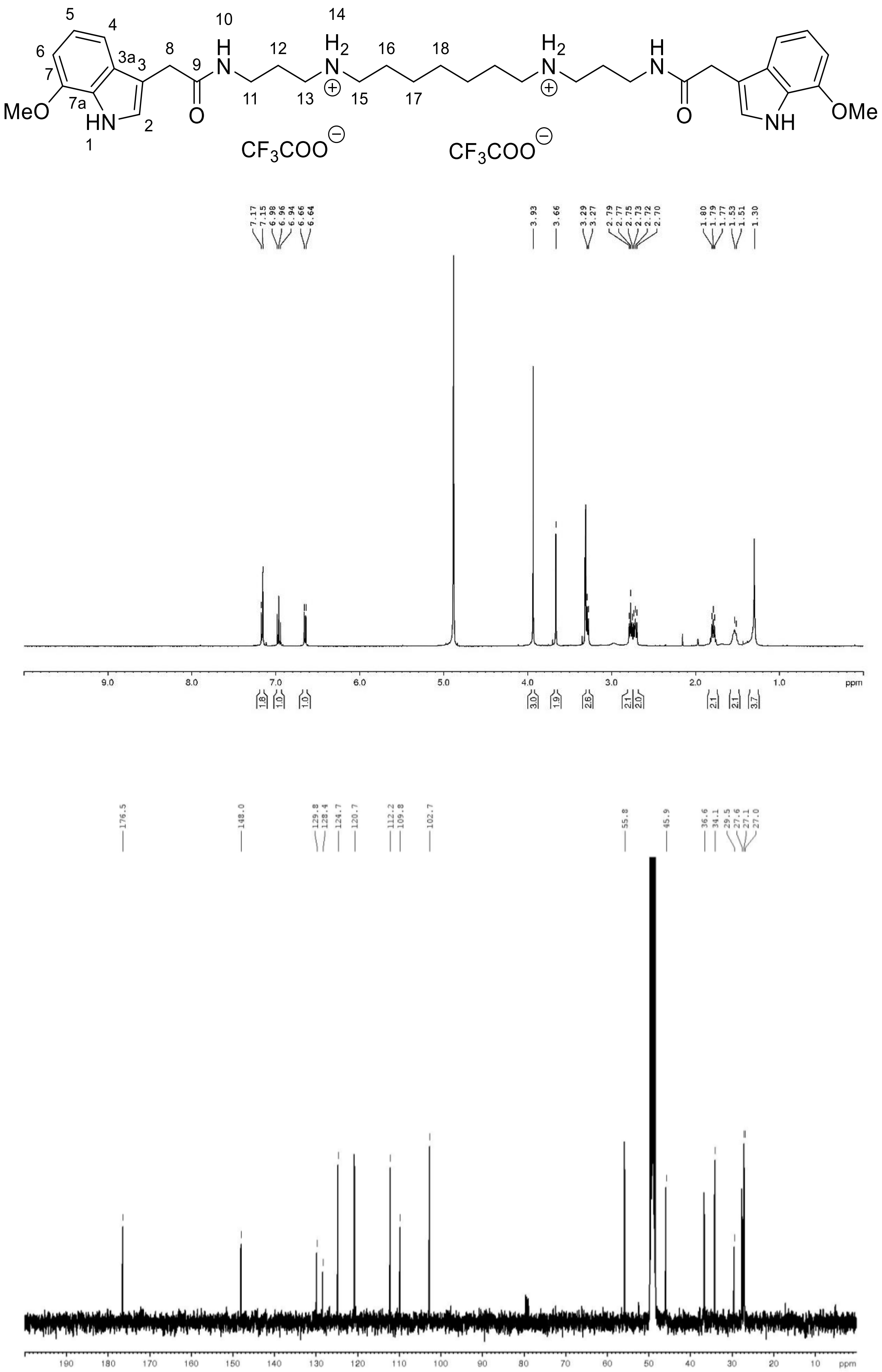

Figure S30 ^{1}H (CD_3OD, 400 MHz) and ^{13}C (CD_3OD, 100 MHz) NMR spectra for **19c**

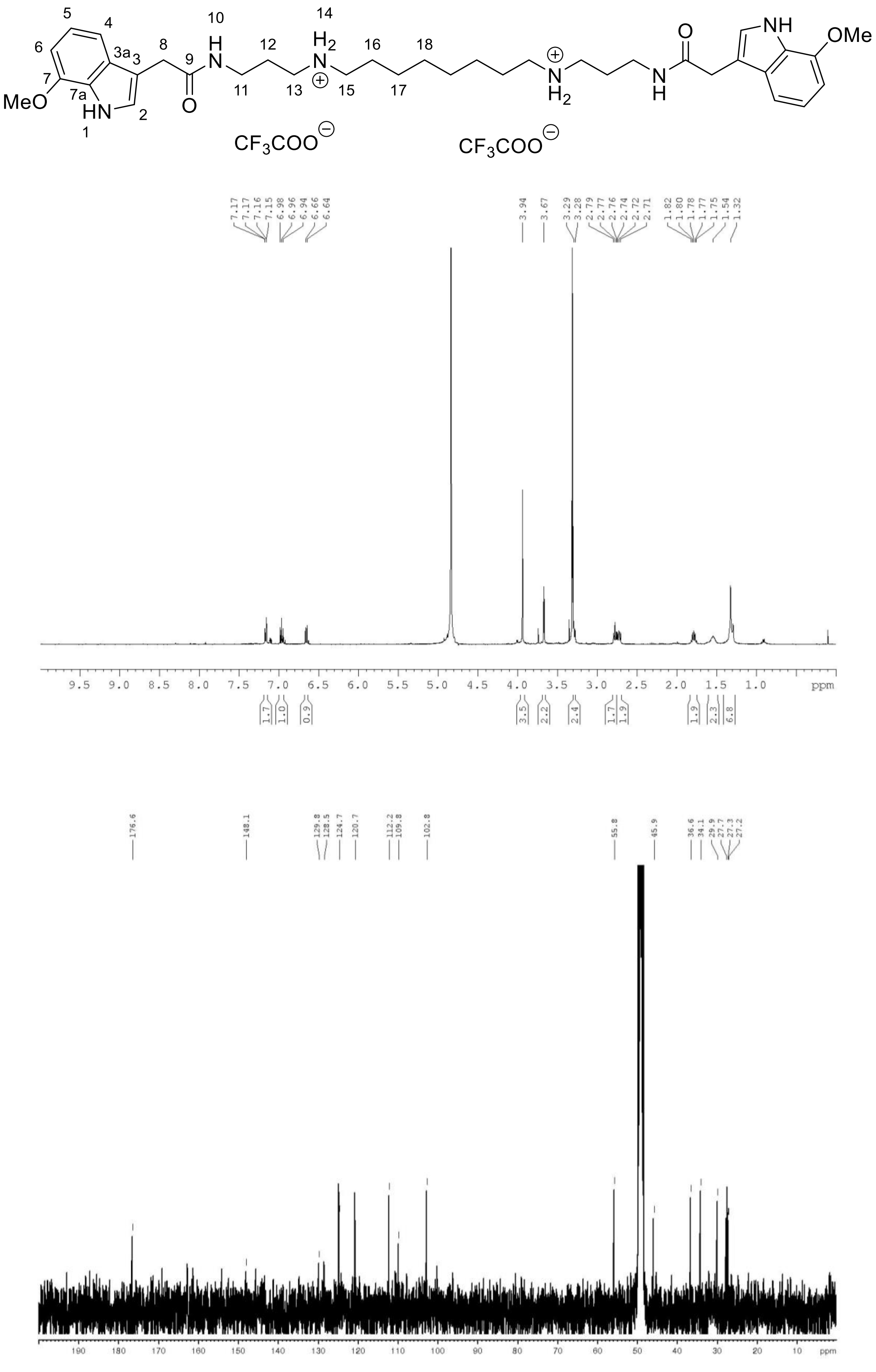

Figure S31 ^{1}H (CD_3OD, 400 MHz) and ^{13}C (CD_3OD, 100 MHz) NMR spectra for **19d**

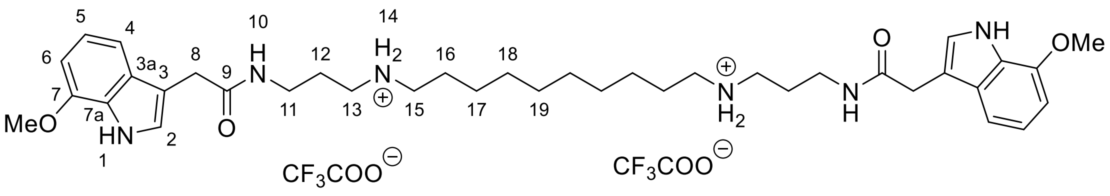

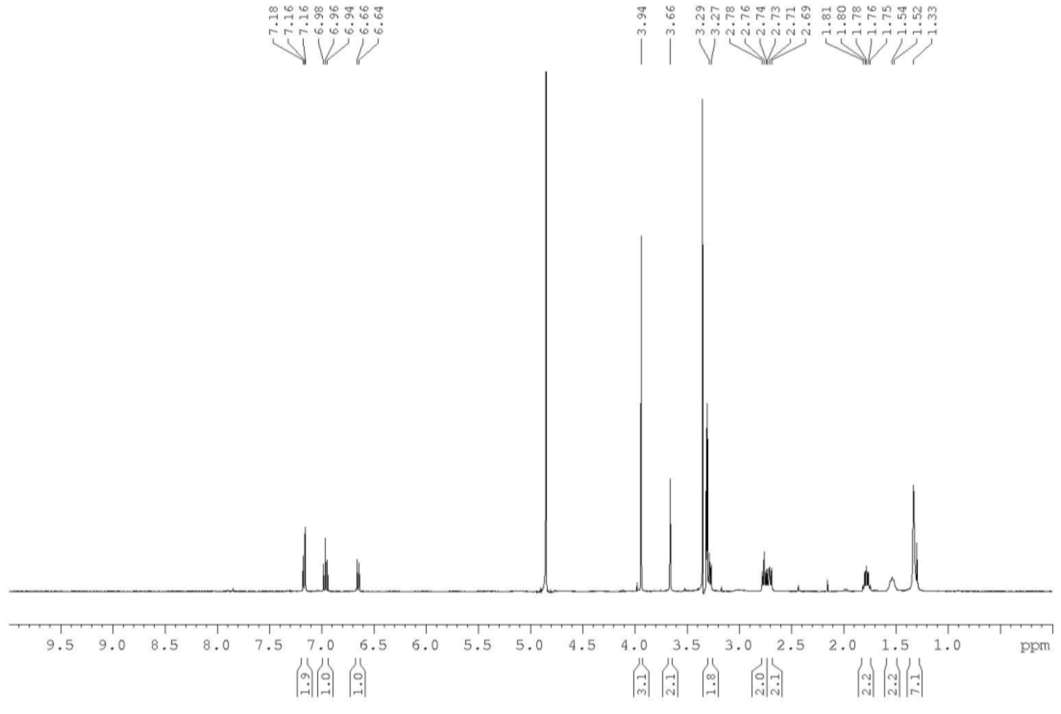

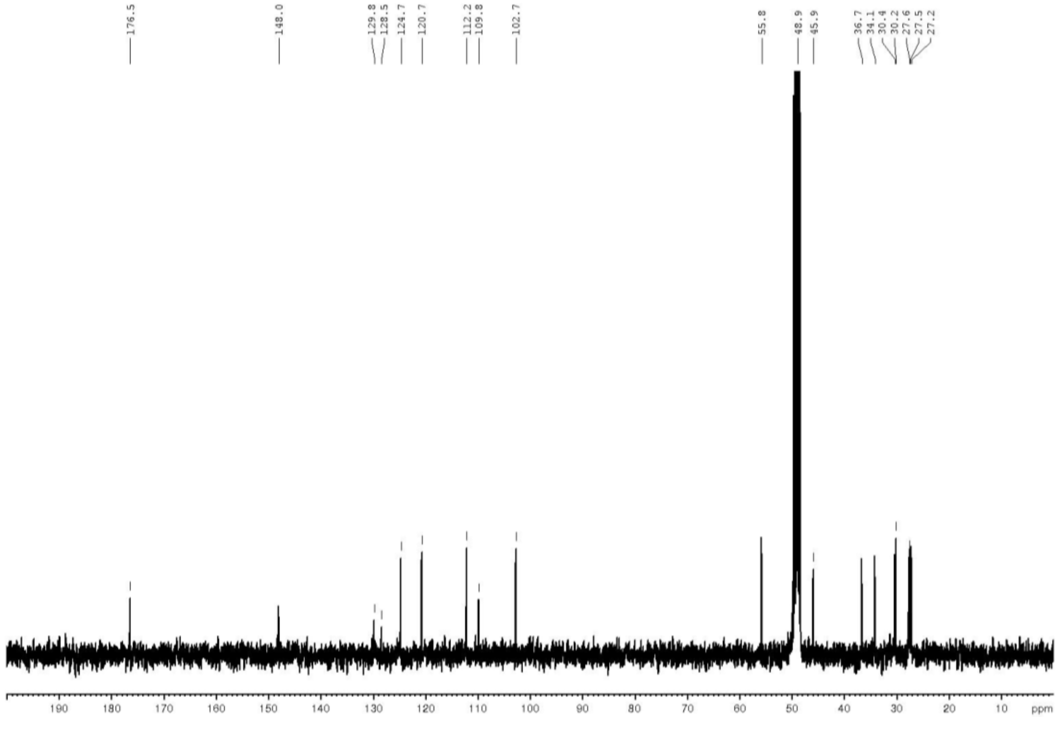

Figure S32 ^{1}H (CD_3OD, 400 MHz) and ^{13}C (CD_3OD, 100 MHz) NMR spectra for **19e**

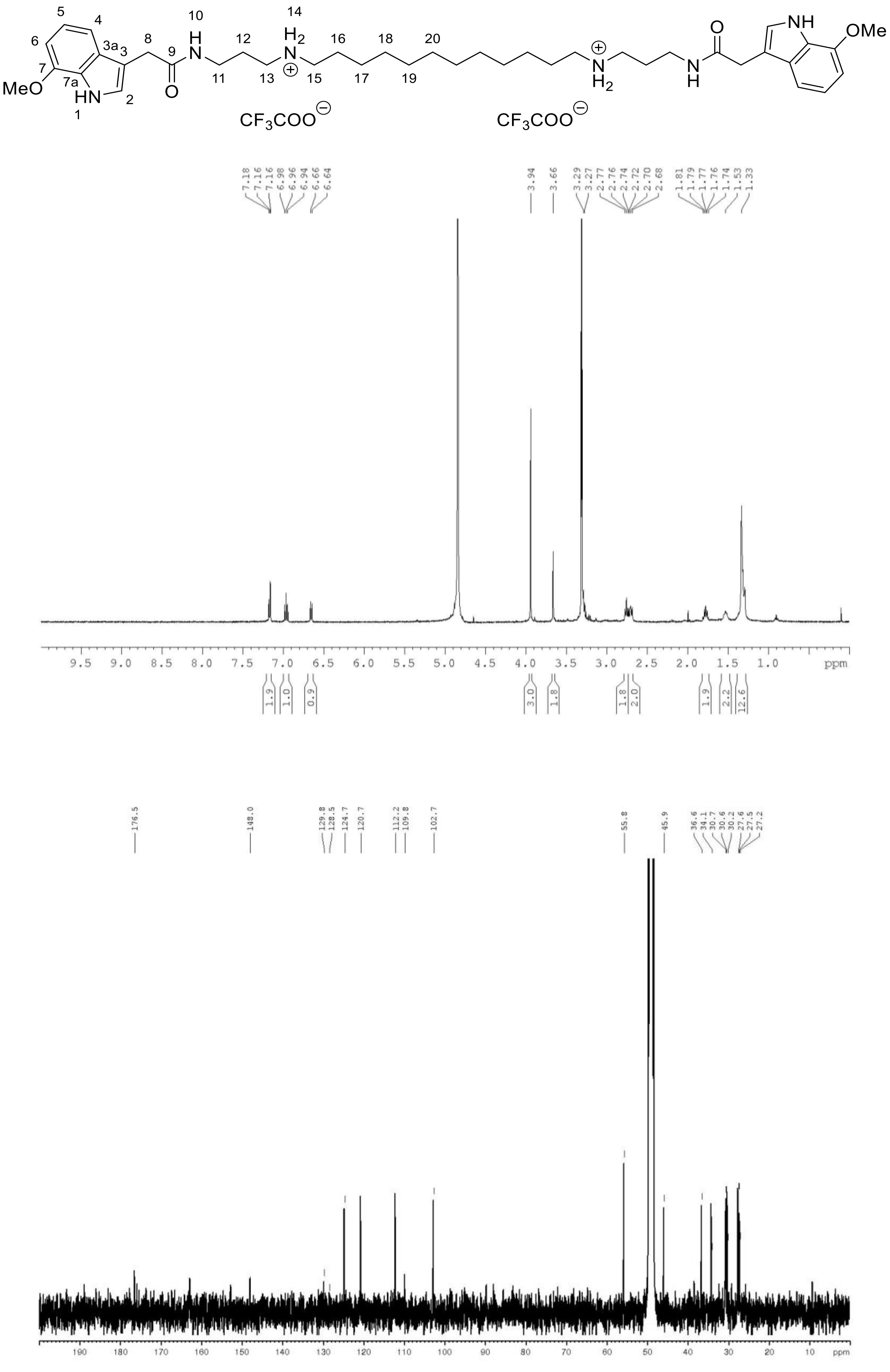

Figure S33 ^{1}H (CD_3OD, 400 MHz) and ^{13}C (CD_3OD, 100 MHz) NMR spectra for **19f**

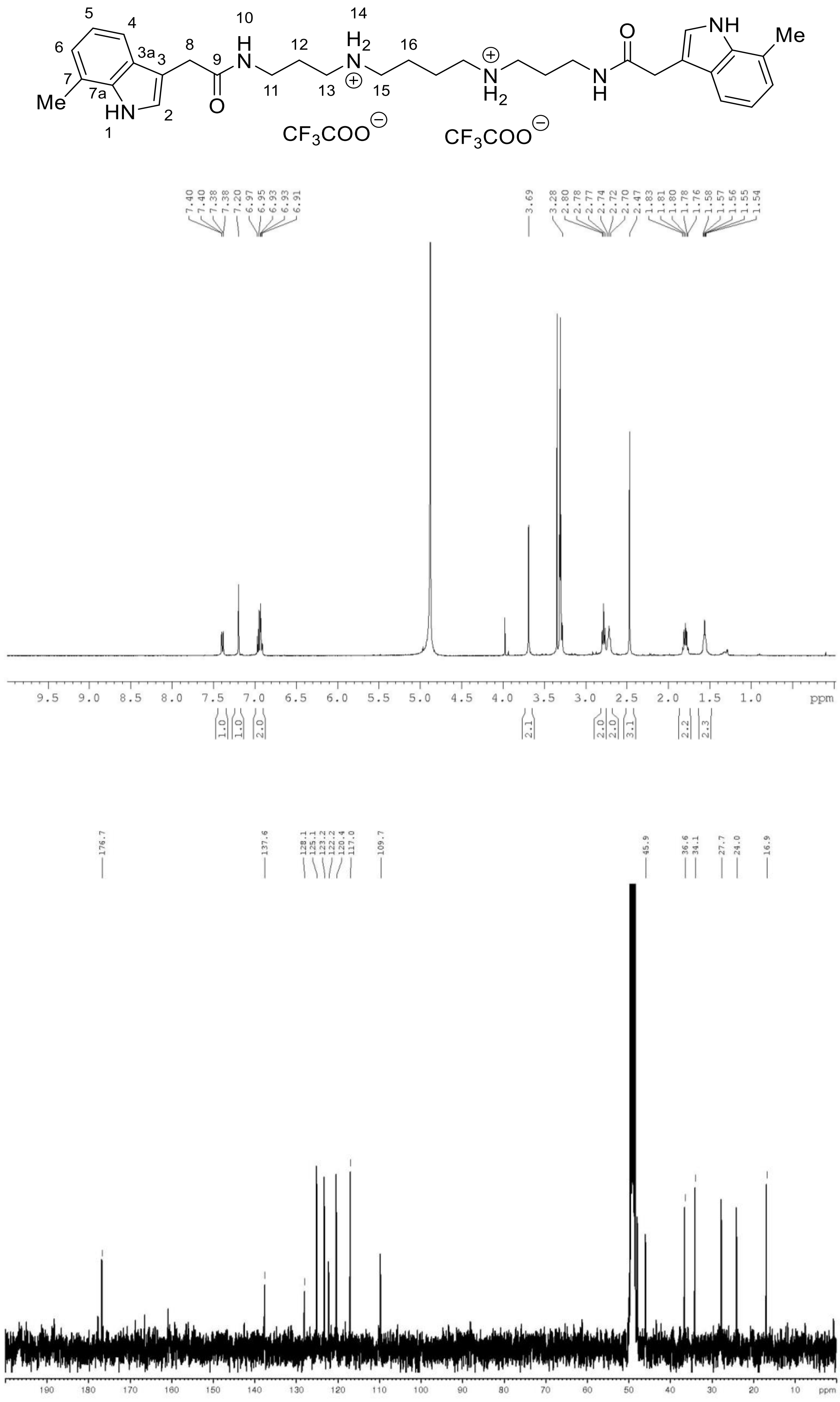

Figure S34 ^{1}H (CD_3OD, 400 MHz) and ^{13}C (CD_3OD, 100 MHz) NMR spectra for **20a**

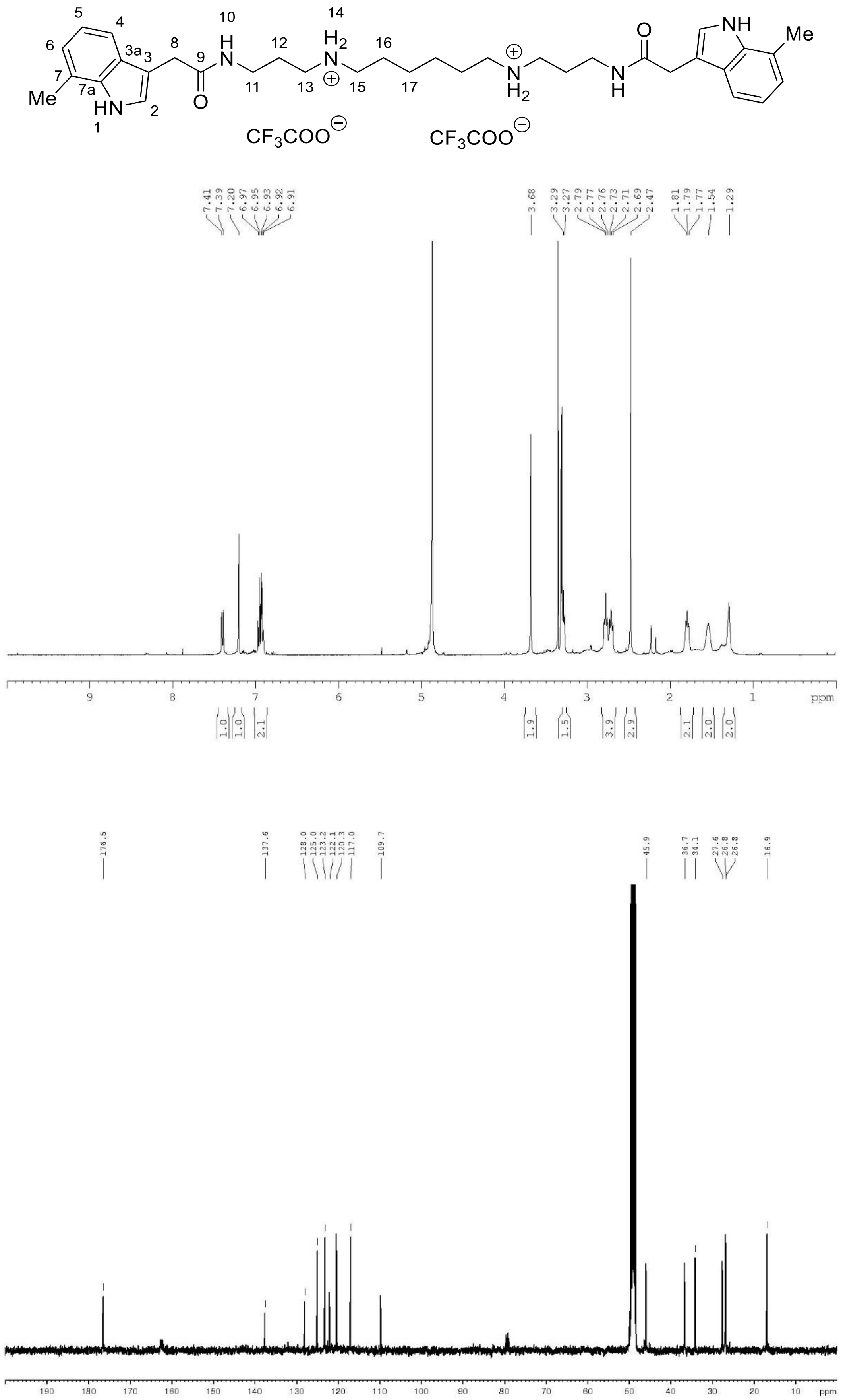

Figure S35 ^{1}H (CD_3OD, 400 MHz) and ^{13}C (CD_3OD, 100 MHz) NMR spectra for **20b**

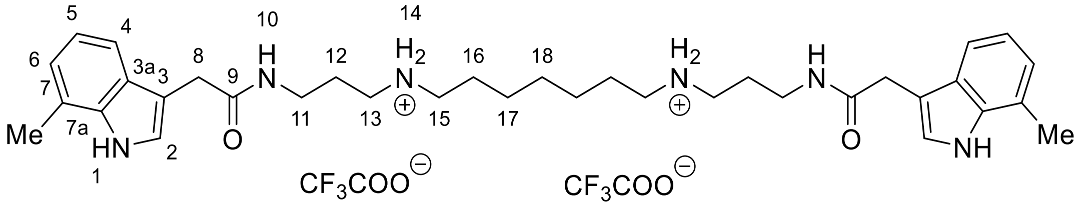

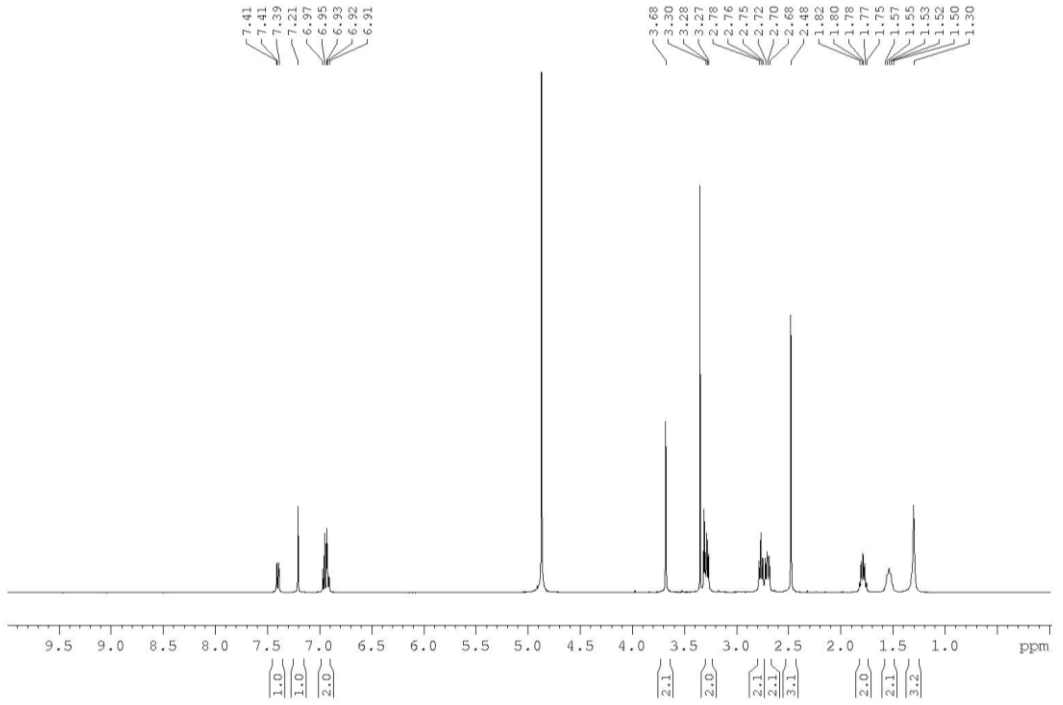

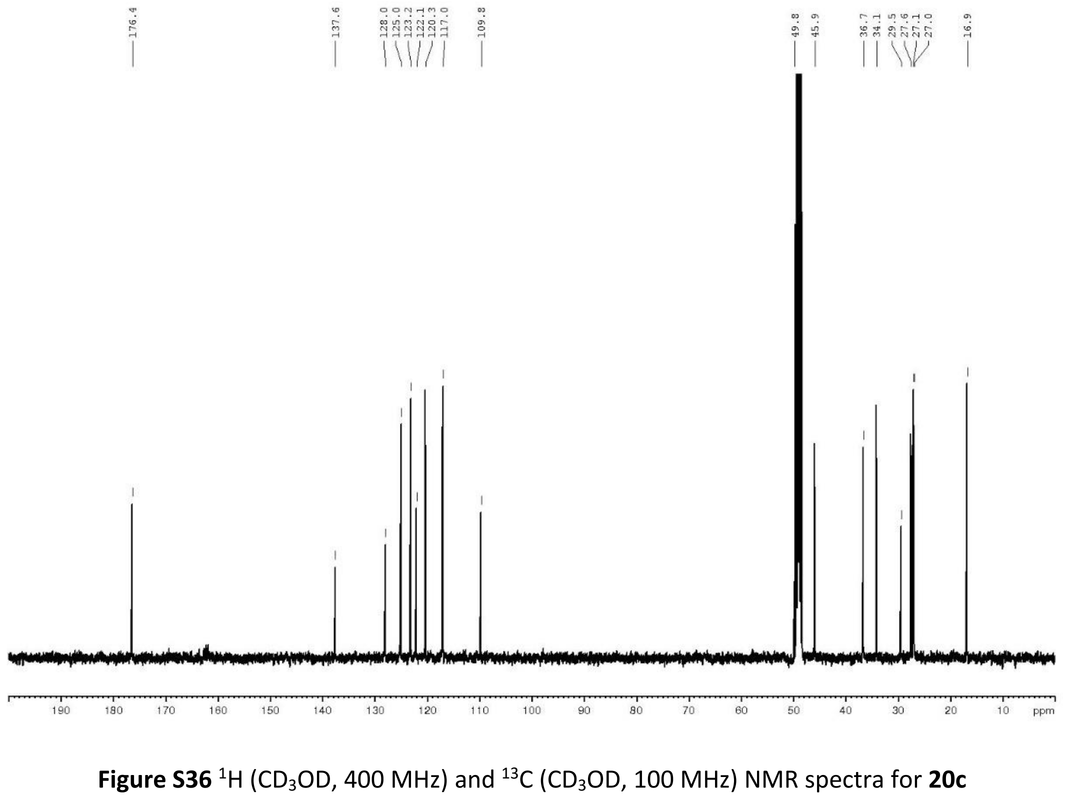

Figure S36 ^{1}H (CD_3OD, 400 MHz) and ^{13}C (CD_3OD, 100 MHz) NMR spectra for **20c**

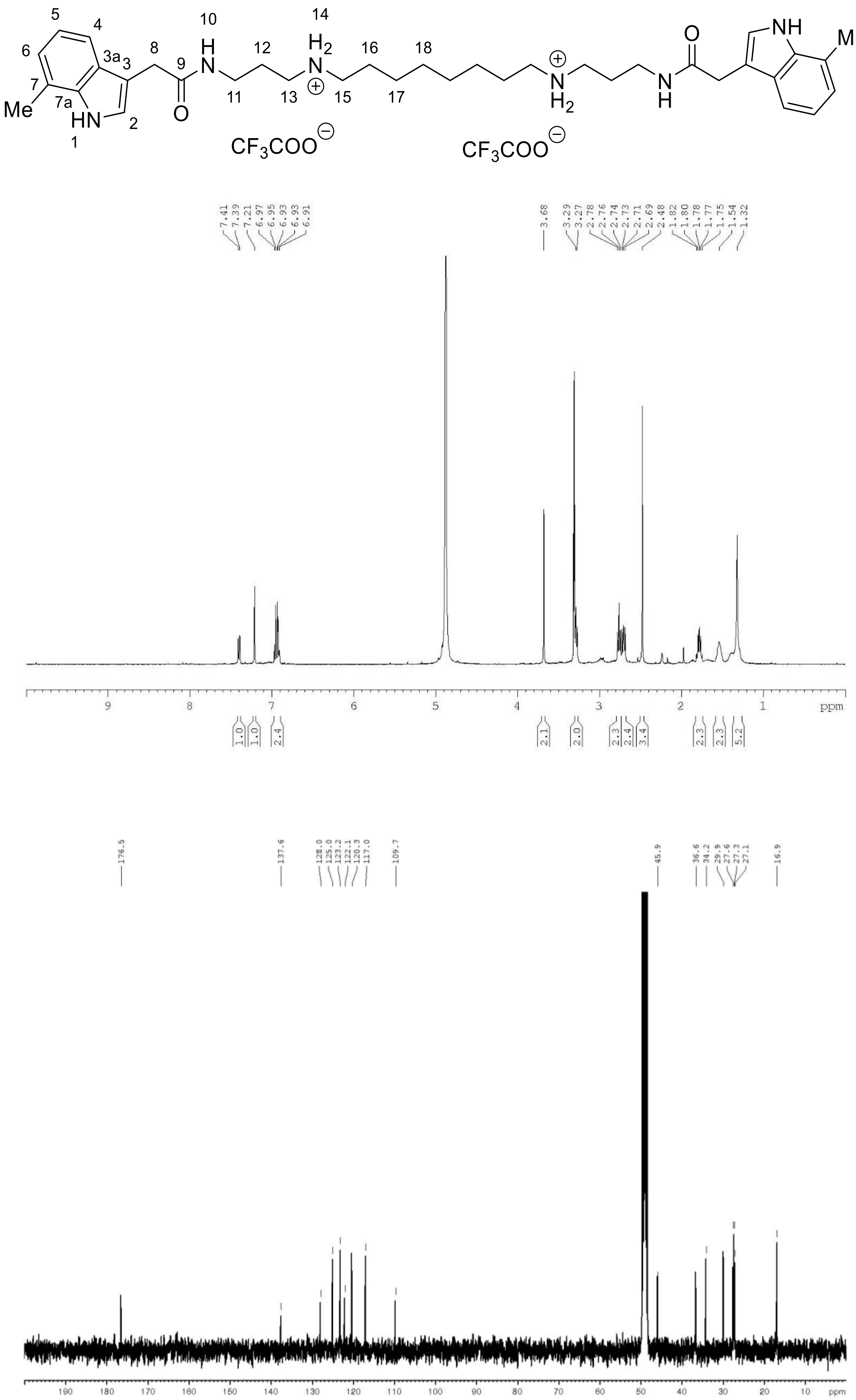

Figure S37 ^{1}H (CD_3OD, 400 MHz) and ^{13}C (CD_3OD, 100 MHz) NMR spectra for **20d**

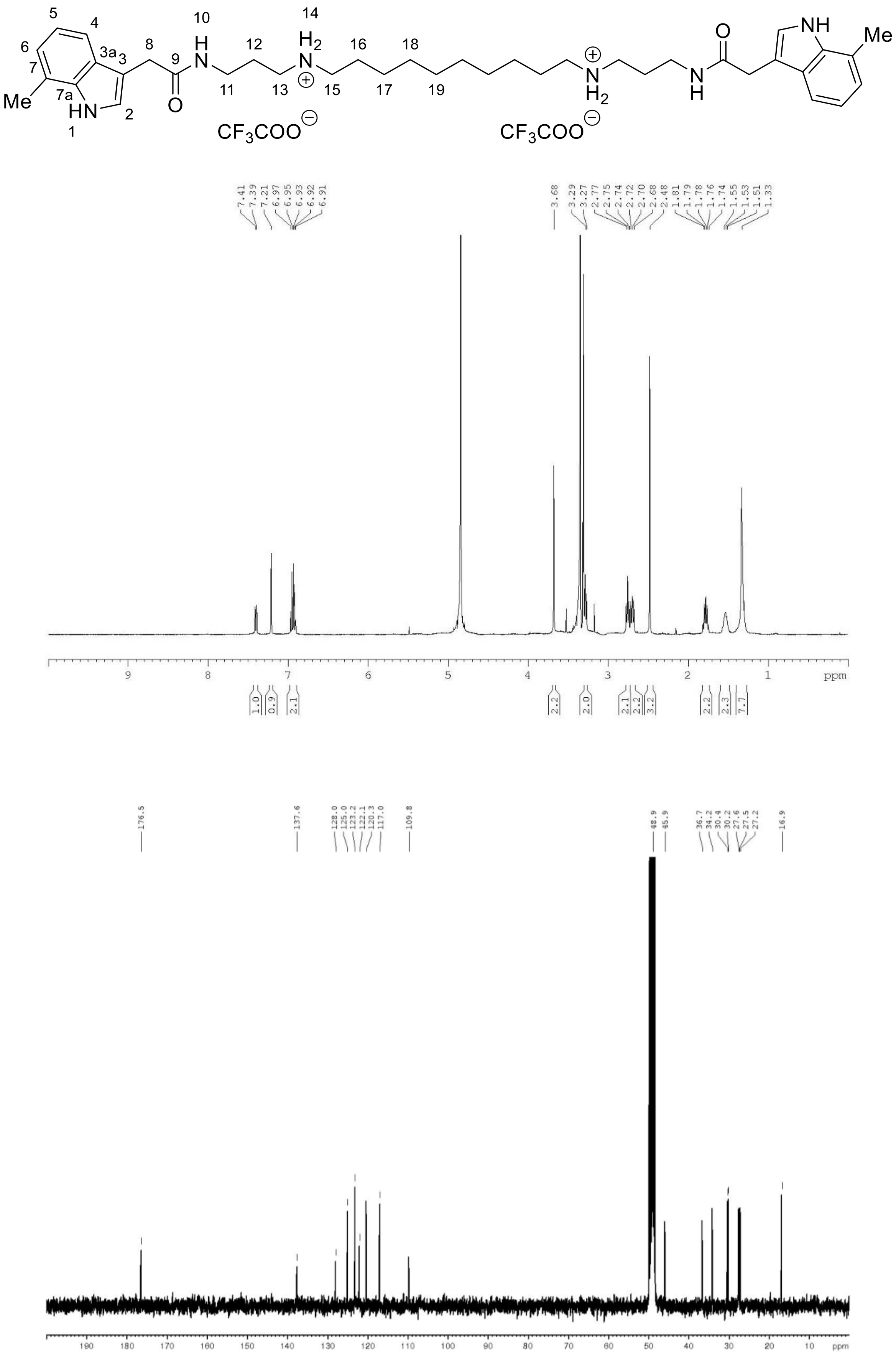

Figure S38 ^{1}H (CD_3OD, 400 MHz) and ^{13}C (CD_3OD, 100 MHz) NMR spectra for **20e**

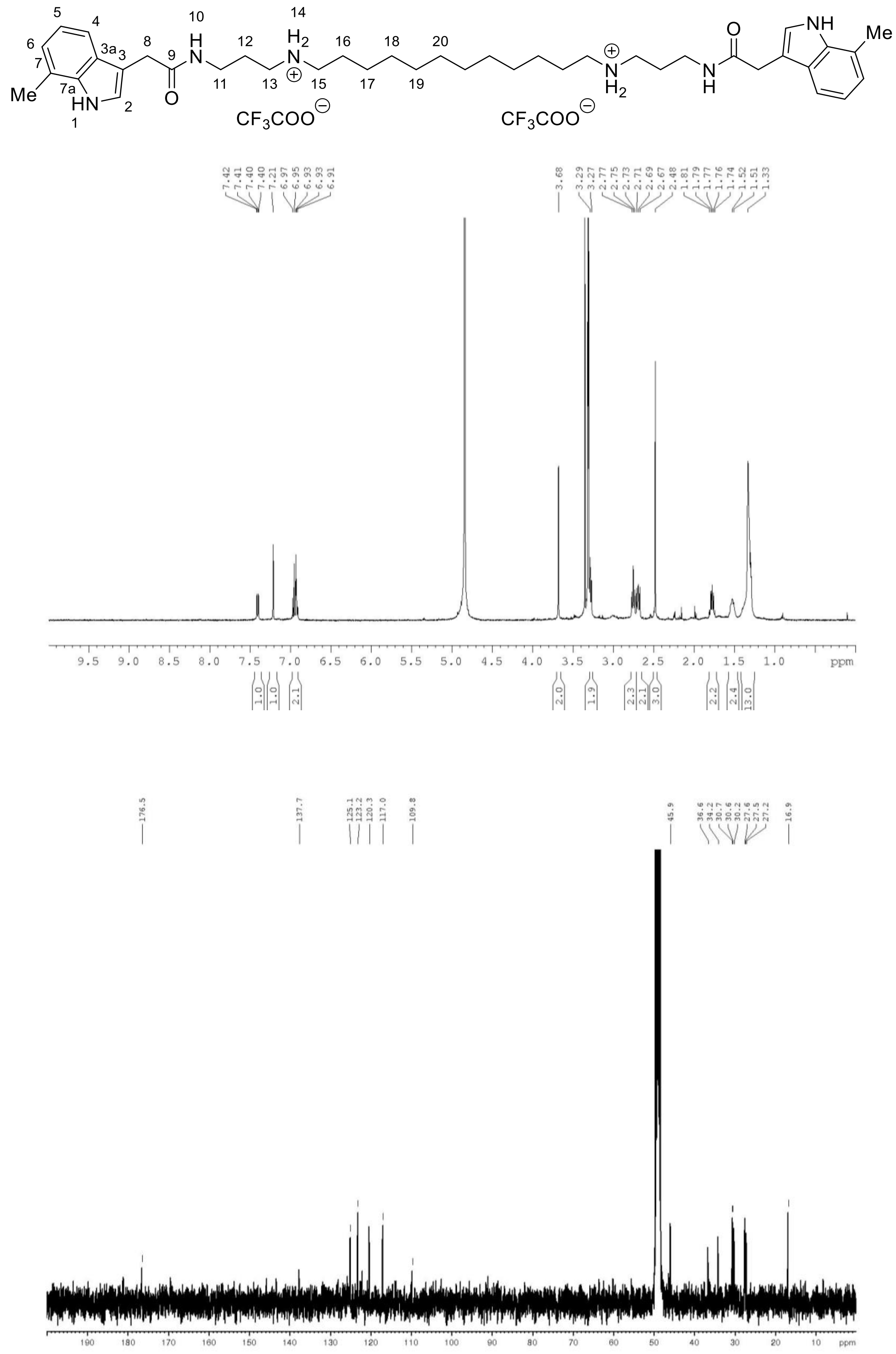

Figure S39 ^{1}H (CD_3OD, 400 MHz) and ^{13}C (CD_3OD, 100 MHz) NMR spectra for **20f**
